# Trilineage Sequencing Reveals Complex TCRβ Transcriptomes in Neutrophils and Monocytes Alongside T Cells

**DOI:** 10.1016/j.gpb.2019.02.004

**Published:** 2021-03-02

**Authors:** Tina Fuchs, Kerstin Puellmann, Chunlin Wang, Jian Han, Alexander W. Beham, Michael Neumaier, Wolfgang E. Kaminski

**Affiliations:** 1 Institute for Clinical Chemistry, University of Heidelberg Medical Faculty Mannheim, D-68167 Mannheim, Germany; 2 Aesculabor Hamburg, D-22769 Hamburg, Germany; 3 iRepertoire Inc., Huntsville, AL 35806, USA; 4 HudsonAlpha Institute for Biotechnology, Huntsville, AL 35806, USA; 5 Department of Surgery, Hospital of Siegen, D-57076 Siegen, Germany; 6 Ingenium Digital Diagnostics, D-87662 Kaltental, Germany

**Keywords:** Monocyte, Neutrophil, T cell, TCRβ transcriptome, Next-generation sequencing

## Abstract

Recent findings indicate the presence of **T cell** receptor (TCR)-based combinatorial immune receptors beyond T cells in **neutrophils** and **monocytes**/macrophages. In this study, using a semiquantitative trilineage immune repertoire sequencing approach as well as under rigorous bioinformatic conditions, we identify highly complex **TCRβ transcriptomes** in human circulating monocytes and neutrophils that separately encode repertoire diversities one and two orders of magnitude smaller than that of T cells. Intraindividual transcriptomic analyses reveal that neutrophils, monocytes, and T cells express distinct TCRβ repertoires with less than 0.1% overall trilineage repertoire sharing. Interindividual comparison shows that in all three leukocyte lineages, the vast majority of the expressed TCRβ variants are private. We also find that differentiation of monocytes into macrophages induces dramatic individual-specific repertoire shifts, revealing a surprising degree of immune repertoire plasticity in the monocyte lineage. These results uncover the remarkable complexity of the two phagocyte-based flexible immune systems which until now has been hidden in the shadow of T cells.

## Introduction

Adaptive immunity in higher vertebrates is built on two recombinatorial immune receptor systems — immunoglobulins and T cell receptor (TCR). The current immunological understanding is that these molecular machineries of variable immune recognition are strictly confined to the lymphoid lineage [1], [2]. Recent findings of variable immune receptor expression in myeloid immune cells now query this longstanding dogma. In 2006, Puellmann et al. [3], [4] demonstrated that subpopulations of neutrophil granulocytes from healthy humans and mice constitutively express TCRs that are based of TCR α- and β-chain (TCRαβ). In humans, the neutrophil TCRαβ is expressed across the entire life-span and has been shown to undergo immunosenescence [5]. Next to the TCRαβ, granulocytes also express the alternative variant of TCR which consists of the γ and δ antigen binding subunits (referred to as TCRγδ) [6], [7]. These unexpected findings demonstrate that neutrophil granulocytes possess the same flexible antigen recognition machinery as T cells. Moreover, recent studies provide conclusive evidence that small subpopulations of monocytes, the second major phagocyte population in the circulation next to neutrophils, also constitutively express TCRαβ variable immune receptors [8], [9]. Importantly, both the TCRαβ in neutrophils and in macrophages have been implicated by us and others in disease [10], [11], [12], [13], [14]. Of note, most recent studies from our laboratory and others provide evidence for the expression of the second variable immune receptor based on immunoglobulin heavy and light chain genes by cells of the myeloid lineage [15], [16], [17].

Proof-of-concept experiments using spectratyping and small-scale standard Sanger sequencing of the hypervariable TCR complementary determining region 3 (CDR3) regions have revealed diverse and individual-specific TCR repertoires in neutrophils and monocytes/macrophages, and thus clearly demonstrate that the TCRαβ expressed by both myeloid phagocyte lineages are combinatorial immune receptors [3], [5], [8]. Based on this, we have put forward the hypothesis that in higher vertebrates variable immune recognition may function on a wider cellular basis than currently thought [18], [19].

CDR3 spectratyping and small-scale sequencing, however, represent biased experimental systems with limited sensitivity that are not suited to assess the true and detailed repertoire diversity of the recently identified extralymphocytic combinatorial TCRs in neutrophils and monocytes/macrophages. With the recent advent of deep sequencing which allows detection of minute quantities of mRNA copies and simultaneous identification of millions of sequences, a technological platform for global analysis of entire transcriptomes at an unprecedented level of resolution has become available [20], [21]. Several methods for immune repertoire profiling by deep sequencing are available [22] and have been widely used in different kinds of studies [23], [24], [25], [26].

Here, we performed a global comparison of TCRβ transcriptomes in neutrophils, monocytes/macrophages, and T cells by cross-sectional deep sequencing in a cohort of healthy individuals. The trilineage transcriptome sequencing was conducted using a method specifically optimized for the analysis of the hypervariable CDR3 regions in recombinatorial TCRs. This approach combined amplicon rescued multiplex (ARM)-PCR which was developed by our laboratory [27] with high-throughput sequencing. ARM-PCR is a multiplex RT-PCR technique that minimizes PCR bias and allows the detection of single transcripts and semiquantitative analysis of transcript frequencies. Moreover, data analysis was conducted using a newly developed computational strategy that rigorously eliminates high-throughput sequencing artifacts [26], [28]. The SMART bioinformatic approach uses a cascade of filters, including sequencing error filter, mosaic sequencing filter, PCR amplification performance filter, reference sequence filter, and frequency threshold filter, to eliminate artifactual sequences at every quality control checkpoints (unpublished data). The results presented here provide global interlineage, interindividual, and intralineage comparisons of the TCRβ transcriptomes expressed by neutrophils, monocytes/macrophages, and T cells under high-stringency bioinformatic conditions.

## Results

### Circulating neutrophils and monocytes express distinct and highly complex TCRβ transcriptomes

To gain a detailed global overview of the range of the TCRβ diversity expressed by neutrophils, monocytes, and T cells in the circulation and to address the question of interindividual variation, we conducted ARM-PCR-based high-throughput TCRβ transcriptome sequencing in a cohort of 5 randomly selected Caucasian individuals (2 males and 3 females, age range: 26–48 years). None of the donors had known illnesses at the time of the blood draw or presented with clinical or laboratory signs of inflammatory disease. We focussed on the analysis of the TCRβ transcriptomes, because the TCRβ CDR3 sequences code for the antigen-binding loop of the mature TCRαβ complex and thus represent an individual’s molecular armamentarium for specific target recognition in host defense.

Highly pure CD15^+^ neutrophils, CD14^+^ monocytes, and CD3^+^ T lymphocytes were separately collected from a single blood draw of each individual using density gradient separation followed by lineage-specific Magnetic Cell Sorting (MACS) purification. The average cell number (mean ± SD) of the purified leukocyte subpopulations was 8.6 × 10^6^ ± 2.5 × 10^6^ (n = 15) and cell purities were > 99.1% as assessed by flow cytometry with the exception of one monocyte preparation which contained only 96% CD14^+^ cells (donor III) (Figure S1). RT-PCR profiling of lineage-specific marker genes demonstrated that no noticeable cross-contamination had occurred between the three leukocyte lineages (Figure S2). In addition, to explore the intralineage dynamics of the TCRβ transcriptome in the monocyte lineage, aliquots of CD14^+^ monocytes from three randomly selected members of the cohort were differentiated *in vitro* into proinflammatory M1 macrophages [29] in the presence of the cytokine IFNγ.

To correct for sequencing artifacts under high-stringency conditions, all sequence reads were subjected to the SMART filtering algorithm (unpublished data). This resulted in approximately 41.74 million effective sequence reads, of which about 41.30 million could be assigned to TCRβ CDR3 intervals (Table S1). Subsequently, all sequence reads were filtered with the SMART frequency threshold set to > 1. This strategy, in which all single-copy TCRβ variants were *a priori* discarded, resulted in a total of 1,431,472 unique TCRβ CDR3 variants from the three peripheral blood leukocyte subpopulations of the five donors and the monocyte-derived M1 macrophages. A TCRβ CDR3 sequence is considered unique if it represents a nonredundant series of amino acids, which is in a stop-codon-free reading frame and contains both translated conserved V_β_ and J_β_ motifs. Of note, conventional bioinformatic analysis yielded a 30% higher total number of unique TCRβ CDR3 sequences (n = 2,121,216), which documents that the SMART filter eliminates significant portions of next-generation sequencing (NGS) errors under high-stringency conditions (Figure S3; Table S1).

Altogether, we identified 598 V_β_-J_β_ combinations among the 1,431,472 unique TCRβ CDR3 nucleotide sequences (Figure S4), which accounted for about 95.8% of the 624 potential V_β_-J_β_ combinations predicted to yield functional rearrangements as cataloged in the ImMunoGeneTics (IMGT) database [30]. Among the 15 samples of freshly collected leukocyte subpopulations, we found no significant differences in TCR V_β_ or J_β_ usage and trimming of nucleotides at the V_β_ and J_β_ coding ends in CDR3 intervals between neutrophils, monocytes, and T cells (Figures S5 and S6). Interlineage comparison of CDR3 length distributions, however, showed that the neutrophils from the majority of the individuals exhibited non-Gaussian patterns (Figure S7), which is in agreement with previous observations relying on CDR3 length spectratyping [3], [5]. Consistent with this, analysis of the addition of nontemplated nucleotides showed non-Gaussian patterns in neutrophils, whereas Gaussian profiles were observed in most of the monocyte and T cell populations (Figure S8). This suggests discrete differences in the V(D)J recombination process between neutrophils and mononuclear cells.

**Figure 1:**
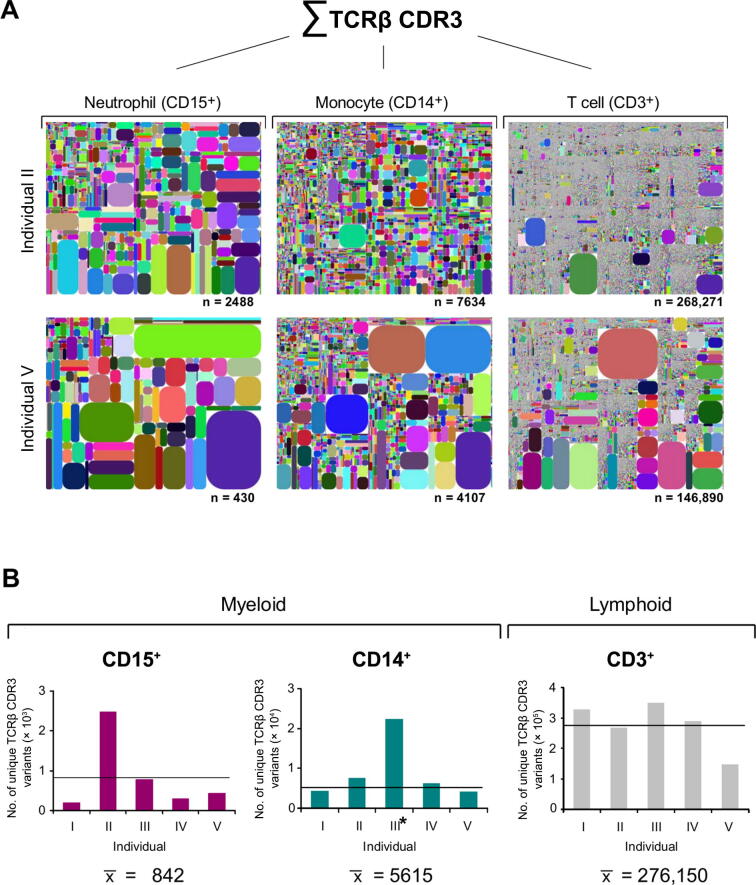
**Trilineage transcriptome sequencing uncovers highly diverse TCRβ repertoires in neutrophils and monocytes** **A.** The diversity plots visualizing the relative abundance of the TCRβ CDR3 transcript variants expressed by human circulating CD15^+^ neutrophils, CD14^+^ monocytes, and CD3^+^ T cells. Two healthy individuals (II and V) are representatively shown whose leukocyte subpopulations were purified from a single blood draw. Each spot in a plot represents a rearranged TCRβ transcript that encodes a unique TCRβ CDR3 sequence. It is defined by a unique color and its area is proportional to the relative transcript frequency. A TCRβ CDR3 sequence is considered unique if it represents a nonredundant fragment of amino acids, which is in a stop-codon-free reading frame and contains both translated conserved V_β_ and J_β_ motifs. The position of each spot within the plot area is defined according to V_β_ usage (x-axis: V_β1_→V_βi_) and J_β_ usage (y-axis: J_β1_→J_βi_). Large spots reflect biases of the underlying immune repertoire. Each plot has a distinct color code. Total number of identified nonredundant TCRβ CDR3 variants is indicated under each diversity tree plot. **B.** Synopsis of unique TCRβ CDR3 variants expressed by circulating myeloid phagocytes (neutrophils and monocytes) and T lymphocytes, respectively, from five healthy individuals (I–V). The average number of the expressed TCRβ CDR3 variants is shown for each lineage at the bottom and is represented by a horizontal line in each column diagrams The CD14^+^ monocyte sample from donor III (labeled by an asterisk) was excluded from the statistical analysis because it contained significant amounts of CD3^+^ cells (Figure S1). CDR3, complementarity determining region 3.

To comprehend and compare the entire diversity of immune repertoire transcriptomes, we developed a software algorithm that allows to visualize repertoire diversity, V-J usage, and the frequency of individual TCRβ CDR3 transcript variants. Using this “diversity tree plot” analysis, trilineage comparison of the TCRβ transcriptomes revealed a unique diversity pattern for each leukocyte lineage in each individual tested (Figure 1**A**, Figure S9). We noted an unexpectedly high repertoire diversity in both the neutrophil and monocyte fractions from all individuals. The average numbers (mean ± SD) of the expressed distinct TCRβ CDR3 variants were 842 ± 845 for neutrophils (CD15^+^, n = 5), 5615 ± 1428 for monocytes (CD14^+^, n = 4), and 276,150 ± 70,572 for T cells (CD3^+^, n = 5), respectively (Figure 1B). The repertoire diversities of unique TCRβ CDR3 sequences ranged from 207 to 2488 for neutrophils (CD15^+^), from 4107 and 7634 for monocytes (CD14^+^, n = 4), and from 146,890 to 348,533 for T cells (CD3^+^). These results confirm that the TCRβ repertoire diversity expressed by T cells in the circulation, which has as yet only been assessed in single individuals in proof-of-concept studies [27], [31], [32], [33], amounts on average to ∼ 3 × 10^5^ variants. More importantly, the average TCRβ repertoire sizes of monocytes and neutrophils and are in the range of 1 × 10^4^ and 1 × 10^3^, and thus differ from T cells by one and two orders of magnitude, respectively.

### The vast majority of the TCRβ variants expressed by neutrophils, monocytes, and T cells are lineage-specific

**Figure 2:**
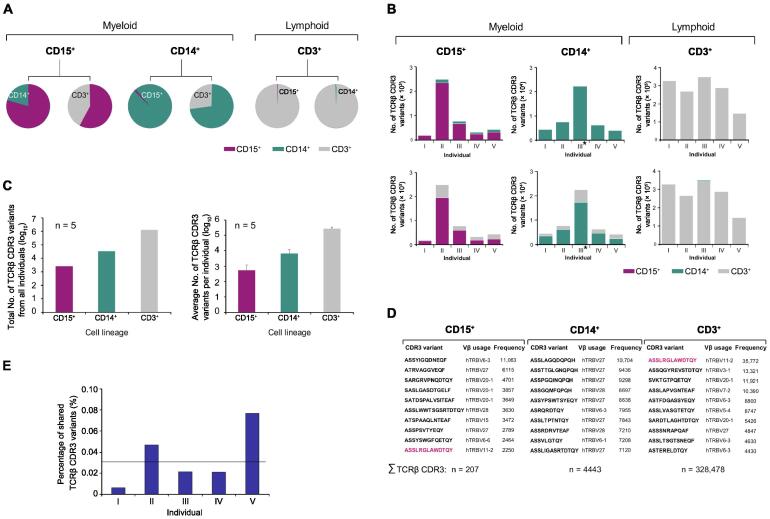
**TCRβ repertoire transcriptomes of neutrophils, monocytes, and T cells are largely lineage-specific** **A.** Pairwise interlineage comparisons of the TCRβ repertoire transcriptomes between human circulating neutrophils (CD15^+^, purple), monocytes (CD14^+^, green), and T cells (CD3^+^, grey), representatively shown for individual IV. For each lineage, the relative proportions of unique TCRβ CDR3 variants that are also expressed by the respective other lineages are shown in the circle graphs. The pairwise interlineage comparisons of the remaining donors are shown in Figure S10. The numbers of the respective shared CDR3 sequences are listed in Table S2. **B.** Interlineage sharing of expressed TCRβ CDR3 variants between neutrophils, monocytes, and T cells. The numbers of shared CDR3 variants are assessed by pairwise comparisons and shown for each healthy individual (I–V) and each leukocyte lineage (CD15^+^, CD14^+^, and CD3^+^). **C.** Total number of exclusive TCRβ CDR3 variants from individuals I–V (left) and average number of exclusive TCRβ CDR3 variants per individual (right) expressed by the neutrophil, monocyte, and T cell lineages, respectively. A CDR3 variant is defined as exclusive if it is expressed by only one individual and by one leukocyte lineage. **D.** Synopsis of the 10 most frequently expressed TCRβ CDR3 variants in each lineage, representatively shown for individual I. Transcript frequencies and the used V_β_ chains are indicated. The total numbers of unique TCRβ CDR3 variants that were identified in the CD15^+^, CD14^+^, and CD3^+^ cells from this individual are shown at the bottom (∑ TCRβ CDR3). A single CDR3 variant that is shared between the CD15^+^ and CD3^+^ lineages is highlighted in purple. **E.** The portions of individual subfractions of the TCRβ transcriptomes displaying common expression in neutrophils, monocytes, and T cells (trilineage expression). The mean value for individuals I–V is indicated by a horizontal line.

We next investigated whether and to which degree the TCRβ transcriptomes in neutrophils, monocytes, and T cells share individual TCRβ variants. To identify shared TCRβ CDR3 sequences, we performed pairwise bioinformatic comparisons of the transcriptomes in all three leukocyte lineages. In all comparisons, the average percentage of TCRβ CDR3 variants that were unique to a lineage was 87.2% (Figure 2A and B, Figure S10; Table S2). This demonstrates that in healthy individuals the vast majority of the TCRβ CDR3 variants in the circulation are expressed in a lineage-specific fashion. In addition, we noted that in each individual both neutrophils and monocytes displayed a higher degree of repertoire sharing with T cells (31.6 ± 10.3 and 26.9 ± 7.1, respectively) than that shared with each other (8.6 ± 9.1, *P* < 0.01).

We then determined the portion of TCRβ CDR3 variants that are solely expressed by one lineage for all five individuals. For this, the TCRβ transcriptomes were corrected for interlineage and interindividual CDR3 sequence redundancies. The total numbers of CDR3 variants exclusive to each lineage were 2653 for neutrophils (CD15^+^), 33,275 for monocytes (CD14^+^) and 1.28 × 10^6^ for T cells (CD3^+^), respectively (Figure 2C, left). The average numbers of exclusive variants per individual were 531 ± 653 for neutrophils (CD15^+^), 6660 ± 5395 for monocytes (CD14^+^), and 273,711 ± 69,992 for T cells (CD3^+^), respectively (Figure 2C, right).

Theoretically, interlineage repertoire sharing can be attributed to genuine expression of the same CDR3 variants by two lineages and/or result from interlineage cross-contamination during cell preparation. To address this question, we conducted cell titration experiments in which either CD15^+^ cells or CD3^+^ cells were mixed with known numbers of CD3^+^ input cells. The quantitative effect of CD3^+^ input cells on the neutrophil TCRβ transcriptome was detectable when the proportion of CD3^+^ cells was 1% and it increased proportionally with growing numbers of CD3^+^ input cells in the cell mix (Figure S11). These cell mixing experiments clearly documented that our ARM-PCR TCRβ transcriptome sequencing approach is a semiquantitative technique which maintains quantitative proportions. This notion was further substantiated by serial template dilution experiments in murine T cells (Figure S12). Importantly, we found no correlation between cell purity and the percentage of TCRβ CDR3 variants that were shared by any two leukocyte lineages (Figure S13; Table S3). Given that ARM-PCR-based transcriptome sequencing is a semiquantitative technique, one would expect that the ranking order of the most frequent CDR3 variants expressed by contaminating cells is proportionally translated into a cell mix as observed in our mixing experiments (Figure S14). However, pairwise analysis of the 50 most frequently expressed CDR3 variants in each of the three lineages did not reflect cross-contamination effects (Figure 2D, Figure S15A–E). This is further supported by the interlineage differential use of the constant chain which we observed for shared CDR3 sequences (Figure S15F). These results show that the CDR3 variants shared between neutrophils, monocytes, and T cells reflect authentic independent co-expression rather than interlineage cross-contamination artifacts, although minor cross-contamination effects cannot be entirely ruled out.

The high degree of lineage-specific TCRβ repertoire expression we observed suggests that only a limited fraction of the TCRβ CDR3 sequences is simultaneously expressed by all three lineages in a given individual. In fact, computational analysis revealed that a minor fraction ranging from 0.006% to 0.08% of each individual’s combined trilineage TCRβ repertoire pool displayed simultaneous expression in neutrophils, monocytes, and T cells (Figure 2E; Table S4A). On average, a 0.03% fraction of CDR3 variants showed trilineage expression. These findings identified a common pool of TCRβ variants in neutrophils, monocytes, and T cells that were directed against identical antigens indicative of a concerted host defense. Interindividual comparison of these common trilineage TCRβ repertoires revealed that they differed considerably between each donor and thus demonstrated that the concerted TCRβ responses are individual-specific (Table S4B).

### Extensive interindividual variation of the trilineage TCRβ repertoire pools

**Figure 3:**
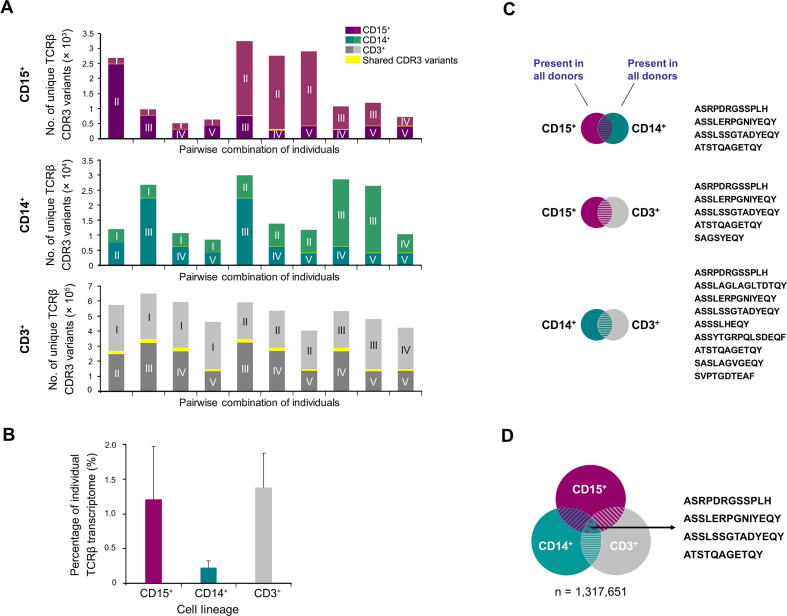
**Extensive interindividual variation of the neutrophil, monocyte, and T cell TCRβ repertoire pools** **A.** Pairwise comparisons of TCRβ transcriptomes between the five individuals within each leukocyte lineage. The fraction of shared TCRβ variants is indicated in yellow. **B.** Average percentage of “public” TCRβ variants expressed by each leukocyte lineage. “Public” TCRβ variants refer to CDR3 sequences that are expressed by all individuals within a lineage. The average percentage for each leukocyte lineage represent the mean ± SD of ratios of each individual’s “public” variants/all variants ratios (n = 5). **C.** Pairwise interlineage comparisons of “public” TCRβ variants between neutrophils, monocytes, and T cells. Detailed sequences of “public” CDR3 variants shared between the indicated leukocyte lineages are shown on the right. **D.** Identification of four trilineage “public” TCRβ variants by global analysis of all trilineage TCRβ transcriptomes.

We then investigated the extent of interindividual variation of the TCRβ repertoires that were expressed by each of the neutrophil, monocyte, and T cell lineages in all individuals. For this, we identified the expressed TCRβ CDR3 variants that were exclusive to a single individual (“private” TCRβ repertoire pool) and the fractions that were shared between individuals. Pairwise comparison of the five TCRβ transcriptomes in each leukocyte subpopulation showed that the vast majority of the expressed CDR3 variants (87.5%–99.7%) were private (Figure 3**A**). These results uncovered a surprising level of variation between the individually expressed TCRβ repertoire pools in all three leukocyte populations. We noted that the average portion of “public” TCRβ sequences, *i.e.*, CDR3 variants that are shared by all individuals, was only 0.2% ± 0.1% in monocytes. In contrast, neutrophils (1.2% ± 0.8%) and T cells (1.4% ± 0.5%) had 6-to 7-fold larger fractions of shared TCRβ CDR3 variants (Figure 3B). In total, we identified 0, 4, and 3447 “public” TCRβ CDR3 sequences exclusive to neutrophil, monocyte, and T cell lineages, respectively (Table S5). Pairwise comparison of “public” TCRβ variants between distinct lineages resulted in the identification of five TCRβ CDR3 variants that were commonly expressed by the neutrophils and T cells from all individuals (Figure 3C). We uncovered nine CDR3 variants that were commonly expressed by the monocytes and T cells from all individuals, and four TCRβ clonotypes displayed neutrophil–monocyte bilineage expression. Overall, we found that only four out of the total number of 1,317,651 exclusive TCRβ variants displayed trilineage expression in all five donors (Figure 3D). These findings uncover the extensive interindividual variation of the TCRβ repertoire pools expressed by neutrophils, monocytes, and T cells, and demonstrate that they predominantly encode private immune repertoires.

### Substantial TCRβ transcriptome changes during monocyte-to-macrophage differentiation

**Figure 4:**
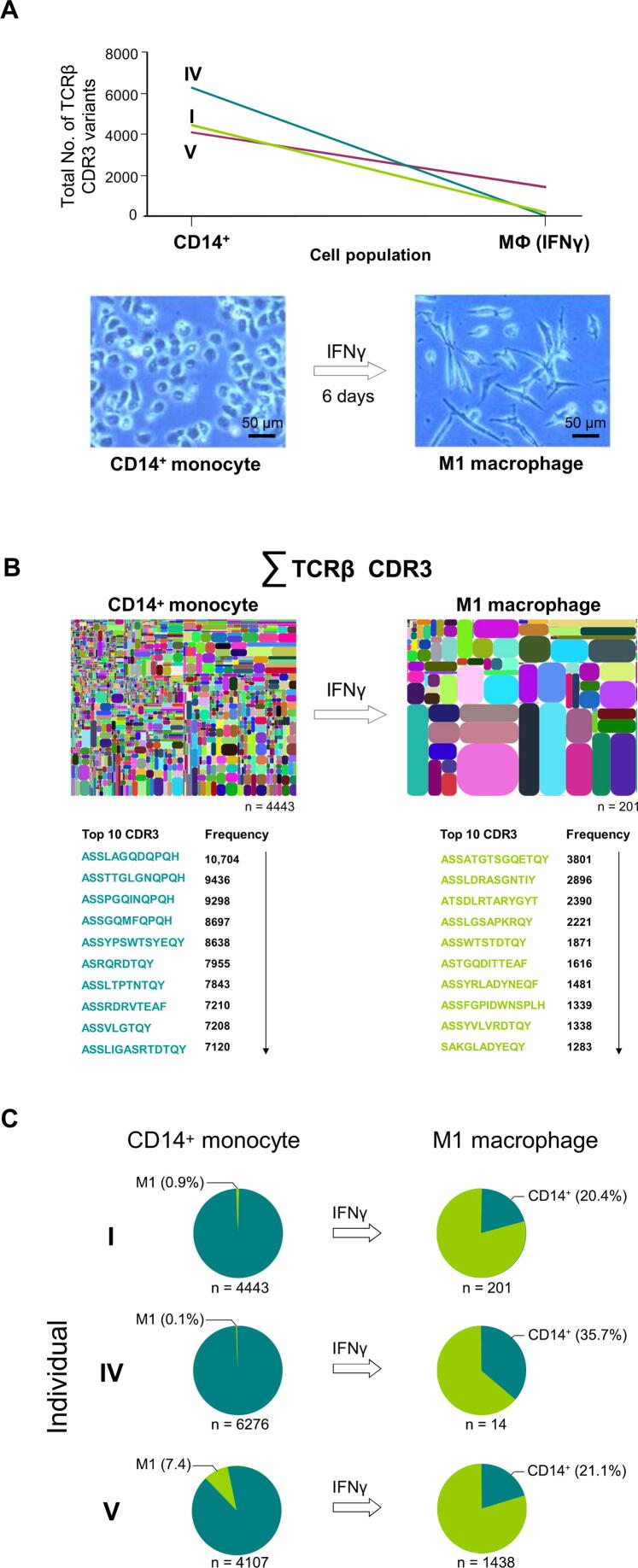
**Large-scale reprogramming of the TCRβ transcriptome of monocytes during differentiation into macrophages** **A.** Comparison of the complete TCRβ transcriptomes of circulating CD14^+^ monocytes and IFNγ-polarized M1 macrophages in three healthy individuals (I, IV, and V). Total numbers of distinct TCRβ CDR3 variants that are expressed before and after monocyte-to-macrophage differentiation are shown. CD14^+^ monocytes were purified from peripheral blood and an aliquot was differentiated into M1 polarized macrophages in the presence of IFNγ for 6 days. Light microscopic images of the cells are representatively shown for one individual. Scale bar, 50 µm. **B.** The diversity plots showing a global view of the extensive repertoire changes during monocyte differentiation into proinflammatory M1 macrophages (representatively shown for individual I). Total numbers of the identified unique TCRβ CDR3 variants are indicated under the plots. The 10 most frequently expressed CDR3 variants in each cell population (bottom) are shown in detail. **C.** Global comparison of the TCRβ transcriptomes of CD14^+^ monocytes (dark green) and M1 macrophages (light green) in the three healthy individuals (I, IV, and V). The percentage of shared CDR3 variants is indicated for each TCRβ transcriptome. The total number of CDR3 sequences exclusively expressed by each population is shown at the bottom of each cake chart.

Next, we sought to explore the intralineage dynamics of the TCRβ transcriptome in myeloid phagocytes. Of particular interest, in this context, is the critical process of monocyte-to-macrophage differentiation by which short-lived monocytes turn into long-lived macrophages and the question whether this process has an impact on TCR repertoire diversity. Small-scale spectratyping experiments have suggested that TCR repertoire changes occur during this process [8]; however, this question has not been addressed systematically and in detail. To obtain a global understanding of TCR repertoire changes during monocyte-to-macrophage differentiation, we analyzed the TCRβ transcriptomes in the monocyte lineage before and after they had undergone *in vitro* differentiation into macrophages. Aliquots from CD14^+^ monocytes from three randomly selected donors (I, IV, and V) were differentiated into proinflammatory M1 macrophages for 6 days (Figure 4**A**). Cell viability of all cultured macrophages was > 95% (data not shown), and total cell counts were similar to those of monocytes (Table S1). We consistently noted a dramatic decline in the total numbers of the expressed TCRβ variants in the M1 macrophage populations from all individuals relative to monocytes (donor I: 4443 → 201; donor IV: 6276 → 14; donor V: 4107 → 1438) (Figure 4A, Figure S16). Thus, *in vitro* differentiation of monocytes into M1 macrophages is sufficient to induce dynamic and individual-specific changes in the entire TCRβ transcriptome. Surprisingly, pairwise comparison of the 10 most frequently expressed TCRβ variants in each individual revealed that the vast majority of these sequences completely differed between the monocyte and the M1 macrophage populations (Figure 4B, Figure S17). This suggests that extensive repertoire changes have occurred during monocyte differentiation into proinflammatory macrophages. Consistent with this, global comparison of the TCRβ transcriptomes showed that 64%–80% of the M1 macrophage repertoires were not expressed by monocytes (Figure 4C; Table S6). These findings strongly suggest that monocytes undergo large-scale reprogramming of their TCRβ transcriptomes during differentiation into M1 macrophages, resulting in the expression of largely distinct TCRβ immune repertoires.

## Discussion

In this study, we report a direct and global comparison of the TCRβ transcriptomes expressed by neutrophils, monocytes, and T cells in human circulation using a semiquantitative large-scale sequencing approach optimized for analyzing immune repertoires. Our interlineage, interindividual, and intralineage TCRβ immune repertoire analyses offer global insights into the as yet unknown complexity and dynamics of the two recently discovered myeloid recombinatorial immune systems at an unprecedented level of resolution.

The first major finding of this study is that the TCRβ transcriptomes of peripheral blood neutrophils and monocytes from healthy individuals encode TCR-like immune receptors of an unexpectedly high repertoire diversity. We find that the circulating pool of neutrophils of healthy individuals expresses on average approximately 1 × 10^3^ TCRβ clonotype variants. This is a 10-fold higher repertoire breadth than suggested by previous experiments using conventional CDR3 spectratyping and standard Sanger sequencing, which predict individual-specific repertoire diversities in the range of 50–100 TCRβ variants [3], [5]. The breadth of an individual’s monocyte TCRβ repertoire is significantly higher with maximum diversities of > 1 × 10^4^ variants. TCRβ repertoire sequencing in our cohort of healthy young individuals reveals that the average TCRβ transcriptome of CD3^+^ cells in the circulation comprises ∼ 260,000 unique TCRβ transcript variants. This is in agreement with recent large-scale sequencing studies, which report expression of ∼ 350,000 distinct TCRβ sequences in purified T cells and ∼ 490,000 TCRβ variants in peripheral blood mononuclear cells (PBMC), respectively, from single individuals under less stringent data filtering conditions [27], [31]. Of note, using our SMART filter (unpublished data) has only a minor quantitative effect on the TCRβ repertoire diversities of all three lineages. In contrast, the rigorous elimination of all single-copy CDR3 variants results in a 50% reduction of repertoire diversity in neutrophils and 30% lower complexities of TCRβ transcriptomes in monocytes and T cells (Figure S3). It is thus possible that our highly stringent data analysis underestimates the true biological diversities of the trilineage TCRβ transcriptomes.

Together, our trilineage analysis indicates that the TCRβ transcriptomes expressed by human monocytes and neutrophils encode highly complex immune receptor systems, whose repertoire diversities are one and two orders of magnitude smaller than that of T cells, respectively. Considering that the leukocyte response in inflammation is typically characterized by the initial recruitment of neutrophils to sites of injury or infection which is followed by a second wave of monocytes [34], [35], [36], this result reveals an inverse correlation between the TCRβ repertoire size and the temporal dynamics of immune cells. Consistent with this, T cells, which are known to exhibit relatively slow temporal dynamics [37], maintain the highest repertoire diversity. A practical implication of our results is that future TCR large-scale sequencing efforts designed to study TCR repertoire diversity in T cells from whole blood or PBMC need to take into account neutrophils and monocytes as potential sources of TCRβ repertoire expression.

The second important finding of this study is that the vast majority of the TCRβ repertoires expressed by an individual’s neutrophils, monocytes, or T cells are lineage-specific. This clearly demonstrates that the three TCR-based combinatorial immune systems target distinct antigen pools, revealing a high degree of operative independence between each receptor system. On the other hand, we observe that subfractions of each individual’s TCRβ transcripts consistently display bilineage or trilineage repertoire sharing. The identification of these shared TCRβ immune receptor variants indicates that neutrophils, monocytes, and T cells are capable of targeting the same antigens and thus may cooperate in concerted immune responses against common targets. Intriguingly, we find that in each individual, neutrophils and monocytes have more TCRβ repertoires in common with T cells than they shared with each other. This suggests a closer cooperation between these myeloid phagocytes and T cells than between neutrophils and monocytes in the setting of specific host defense. It will be challenging to determine whether this reflects a temporal hierarchy in the targeting of common antigens between the fast-acting phagocytic system and the slower-acting T lymphoid system. Theoretically, the TCR repertoires in neutrophils and monocytes could be generated by contaminating T cells in the cell preparations. In our hands, we find no evidence for contamination issues, which was also confirmed by several control experiments.

Third, we find that in all three distinct leukocyte populations, more than 87% of the individually expressed TCRβ repertoire pools are private, *i.e.*, unique to a given individual. Thus, although neutrophils, monocytes, and T cells differ considerably in the size and composition of their TCRβ repertoire pools, they display an equally high degree of individual-specific TCRβ repertoire expression. This suggests that the three flexible TCR-based receptor systems possess a similar individual immune plasticity.

Finally, analysis of the intralineage dynamics of the TCRβ transcriptome in the monocyte lineage reveals large-scale reprogramming of the monocyte repertoire pool during IFNγ-induced monocyte differentiation into proinflammatory macrophages. This observation identifies IFNγ as a potent modulator of the monocyte TCRβ transcriptome. Although it is currently unknown whether this is also the case under *in vivo* conditions, our results uncover an enormous repertoire plasticity of the monocyte/macrophage recombinatorial immune receptor system. Also, it is unclear whether the dramatic repertoire changes that occur during monocyte-to-M1 macrophage differentiation represent a regulatory response merely at the transcription level or at the level of rearrangement. The latter seems not unrealistic in light of the fact that monocytes express the RAG1/RAG2 recombinase complex and the terminal deoxynucleotide transferase, which are integral components of the V(D)J recombination machinery [7]. It will be challenging to address this fascinating possibility in more detail.

Of note, new findings demonstrate the expression of variable immunoglobulins in monocytes and macrophages [15], [16], [17]. With this in mind it will be interesting to analyze the complete immunological armamentarium of myeloid and lymphoid cells in detail.

Taken together, the detailed molecular snapshot of the steady-state TCRβ immune repertoires expressed by human neutrophils, monocytes, and T cells in the circulation uncovers a scale of complexity of the neutrophil and monocyte/macrophage TCRβ transcriptomes that surpasses previous estimates by one or two orders of magnitude.

Our findings define the molecular basis for exploring a new dimension in immunology — the interplay between the TCR-based combinatorial host defense machineries of neutrophils, monocytes/macrophages, and T lymphocytes.

## Materials and methods

### Study cohorts

The cohort of healthy individuals (n = 5) was recruited by random selection from the group of 20–50-year-old male and female members of the staff of the University Hospital Mannheim. Exclusion criteria were: clinical or laboratory signs of acute infection and inflammation, primary hematologic disorders, immunosuppressive therapy, or a history of acquired or congenital immunodeficiency disorders.

### Isolation of leukocyte subpopulations and culture of monocytes/macrophages

Highly pure human neutrophils, monocytes, and T lymphocytes were isolated by ficoll gradient separation followed by CD15^+^, CD14^+^, and CD3^+^ MACS (Miltenyi Biotec), respectively, as previously reported [5], [8]. For each individual, all three leukocyte lineages were purified from a single blood draw. The culture of CD14^+^ monocytes was carried out for 6 days in X-VIVO 10 serum-free and endotoxin-free medium (Cambrex) at a concentration of 5 × 10^5^ cells/ml in the presence of IFNγ (1000 U/ml) (PeproTech) [8]. After 24-h culture, cells were washed and medium was changed. Purity of isolated cell preparations was determined by flow cytometry. Immediately after isolation, all cell populations were resuspended in RNAprotect reagent (Qiagen).

### ARM-PCr

ARM-PCR is a next-generation application of the semiquantitative Templex PCR method [38]. At the exponential phase of the PCR amplification, both methods use universal primers to minimize target sequence-specific PCR amplification biases. From each leukocyte lineage, total RNA was transcribed into cDNA using the One-Step RT-PCR Kit (Qiagen), and the TCRβ transcriptomes were amplified by ARM-PCR utilizing a set of nested TCRβ gene-specific primers as previously reported [27]. These included two forward and two reverse nested primers to simultaneously amplify each possible combination of V and J segments (Forward-out, Fo; Forward-in, Fi; Reverse-out, Ro; Reverse-in, Ri). To all internal primers (Fi and Ri), a pair of common sequence tags was linked. After incorporation of the tag sequences into the PCR products during the first amplification cycles, the exponential amplification phase was conducted using a pair of communal primers that were specific for the tag sequences [27].^.^ In the first amplification round (15 cycles), only TCRβ gene sequence-specific nested primers were used, and then the first-round PCR products were subjected to the second amplification round (40 cycles) using the Multiplex PCR Kit (Qiagen).

### Assessment of ARM-PCR performance

For reliable characterization of immune repertoire diversity, two important criteria need to be fulfilled by the ARM-PCR amplification technique: 1) high reproducibility (*i.e.*, the variability of products that are amplified from identical samples is minimized), and 2) semiquantitation (*i.e.*, relative quantitative proportions of the input sequences are maintained). To assess the test–retest reliability of ARM-PCR, two identical aliquots of the same input sample were subjected to ARM-PCR reactions using V/J primers specific for all human TCR chains, and the products were subsequently sequenced in one and the same sequencing run. We found that the resulting correlation coefficients between both input aliquots were 0.99 for distinct V-J combinations and 0.93 for the 10.000 most abundant CDR3 sequences tested (Figure S18). These high correlation coefficients revealed the high test–retest reliability for the ARM-PCR amplification procedure. To authenticate that ARM-PCR is a semiquantitative method that maintains relative quantitative proportions, serial dilutions of defined TCR cDNA templates were amplified by ARM-PCR and then subjected to high-throughput sequencing; the copy numbers of the specific TCRβ CDR3 variants were determined (Figure S12). The defined TCRβ transcripts were obtained from T cells from the mouse TCR transgenic lines OT-II.Rag1^−/−^ [39] and CBir1.Rag1^−/−^ [40], respectively, and mixed with T cells from the wild-type mice (C57BL/6).

### High-throughput sequencing

High-throughput sequencing of the TCRβ CDR3 regions was performed on a HiSeq2000 DNA sequencer (Illumina) using the standard Illumina sequencing protocols.

### Alignment of TCRβ immune repertoire sequences

Paired-end sequence reads were joined together through overlapping alignment with a modified Needleman-Wunsch algorithm [41], [42]. Subsequently, the merged sequences were mapped to germline V, D, and J reference sequences according to the method we previously reported [27] with the exception that the BLASTn program was used as mapping program with default parameters. Next, the boundaries of the TCRβ CDR3 regions were identified by mapping the corresponding positions from mapped germline reference sequences to joined reads. A TCRβ CDR3 region was defined as the segment that contains all amino acids flanked by the conserved amino acid sequences Y[YFLI]C (3′ end of a V gene segment) and [FW]GXGT (J segment; where X represents any of the 20 amino acids).

### Data filtering using the SMART strategy

The newly developed five-step SMART filtering strategy was used for data analysis (unpublished data). This advanced computational approach utilizes a cascade of filtering algorithms that eliminate artifactual sequences at five distinct quality control checkpoints: 1) sequencing error filter, 2) mosaic sequencing filter, 3) PCR amplification performance filter, 4) reference sequence filter, and 5) frequency threshold filter; it can detect > 99% of erroneous sequences in the immune repertoire. The SMART strategy was employed on all mapped CDR3 reads and the frequency threshold filter was set to > 1. Consequently, all single-copy TCRβ CDR3 sequence variants were discarded. For optimal visualization of the complex TCRβ transcriptome data, novel software applications were developed which included the Tree Map, 3D-Map, and V-J usage plots.

## Ethical statement

Written informed consents were obtained from all individuals enrolled in the study. This study was approved by the ethics committee of the Faculty of Medicine, Mannheim, University of Heidelberg, Germany (Permit No. 2007-254N-MA).

## Data availability

The raw sequencing data have been deposited in the NCBI Sequence Read Archive (SRA: SRA060019), and are publicly accessible at https://www.ncbi.nlm.nih.gov/sra.

## CRediT authorship contribution statement

**Tina Fuchs:** Conceptualization, Investigation, Resources, Writing – review & editing. **Kerstin Puellmann:** Conceptualization, Validation, Data curation. **Chunlin Wang:** Methodology, Software, Validation, Formal analysis. **Jian Han:** Methodology, Project administration. **Alexander W. Beham:** Conceptualization, Methodology. **Michael Neumaier:** Supervision, Project administration, Funding acquisition. **Wolfgang E. Kaminski:** Conceptualization, Data curation, Writing – original draft.

## Supplementary material

Supplementary Figure S1 **Individual cell purities of human peripheral blood CD15+ neutrophils and CD14+ monocytes** Flow cytometry demonstrates individual cell purities of human peripheral blood CD15^+^ neutrophils and CD14^+^ monocytes isolated from donors I–V. The respective percentage of CD3^+^ panT cells are indicated.

Supplementary Figure S2 **RT-PCR expression profiling of the TCRβ chain and T lymphoid markers in purified CD15**^+^**neutrophils, CD14**^+^**monocytes, IFNγ macrophages and CD3**^+^**T cells** The human peripheral blood CD15^+^ neutrophils, CD14^+^ monocytes and CD3^+^ T cells isolated from donors I–V constitutively express the genes for the TCRβ constant chain. Purity of the neutrophil and monocyte samples is demonstrated by the absence of the T cell marker CD2 and CD8, respectively. In addition, the RT-PCR results of IFNγ polarized M1 macrophages are shown for one representative individual (donor IV). Peripheral blood monocytes were isolated by CD14-MACS and differentiation into Th1 (IFNγ) polarized MΦ was induced for 6 days. GAPDH is shown as reference. GAPDH: Glyceraldehyde 3-phosphate dehydrogenase.

Supplementary Figure S3 **High-stringency data analysis of the trilineage TCRβ transcriptomes using the five-step SMART filtering strategy** High-stringency data analysis of the TCRβ transcriptomes in human peripheral blood neutrophils, monocytes and T cells using the five-step SMART filtering strategy. The SMART filter eliminates sequencing artifacts at the following quality control checkpoints: sequencing errors, mosaic formation, PCR amplification errors, reference sequence errors and frequency threshold. Note the significant reduction of erroneous TCRβ CDR3 reads. Unfiltered sequence reads and paired-end filtering (conventional data filter) are shown for comparison. The frequency threshold filter was set >1 which excludes single copy CDR3 transcripts.

Supplementary Figure S4 **V_β_/J_β_ gene usage (3D-Maps) of the TCRβ repertoires expressed by human neutrophils, monocytes and T cells** V_β_/J_β_ gene usage of the TCRβ CDR3 repertoires expressed by human neutrophils (CD15), monocytes (CD14) and T cells (CD3). 3D-Maps in which each expressed V_β_x/J_β_y combination is represented by a bar whose height corresponds to its expression frequency are shown for individuals I–V (A–E). hTRBVx and hTRBJy denote individual human TCR variable V_β_-genes and J_β_-genes, respectively, according to the nomenclature used by the IMGT/GENE database (http://www.imgt.org/IMGTindex/IMGTgene-db.html). x-axis: V_β_ gene; y- axis: J_β_ gene; z-axis: expression frequency.

Supplementary Figure S5 **V_β_/J_β_ gene usage (2D-plots)** V_β_/J_β_ gene usage of human peripheral blood neutrophils (CD15), monocytes (CD14) and T cells (CD3), respectively. The 2D-plots show the relative usage of the V_β_ (top) and J_β_ (bottom) genes for each individual (A–E). The results are normalized to eliminate copy number biases. Note that the three leukocyte lineages do not differ in their V_β_ and J_β_ gene usage. X-axis: V_β_ gene/J_β_ gene; y-axis: percentage of used V_β_/J_β_ genes.

Supplementary Figure S6 **V_β_/J_β_ trimming** V_β_ and J_β_ nucleotide trimming of the CDR3 sequences expressed by human peripheral blood neutrophils (CD15), monocytes (CD14) and T cells (CD3). Bar graphs show the relative distribution of nucleotide trimming at the coding ends of the germline V_β_ (top) and J_β_ gene segments (bottom, individuals I–V). The results are normalized to eliminate copy number biases. X-axis: number of trimmed nucleotides [nt]; y-axis: percentage of CDR3 sequences per number of trimmed nucleotides.

Supplementary Figure S7 **Length distribution of expressed TCRβ CDR3 regions** Length distribution of the neutrophil (CD15), monocyte (CD14) and T cell (CD3) TCRβ CDR3 regions expressed by individuals I–V. Bar graphs indicate relative percentages of individual CDR3 lengths. Note that most individuals exhibit non-Gaussian length distribution patterns.

Supplementary Figure S8 **Nontemplated nucleotide addition** Relative distribution (%) of nontemplated nucleotides added to the CDR3 regions expressed by neutrophils (CD15), monocytes (CD14) and T cells (CD3). Data are corrected for copy number biases. Note the non-Gaussian patterns in neutrophils, whereas Gaussian profiles were observed in most of the monocyte and T cell populations.

Supplementary Figure S9 **Diversity tree plots of TCRβ CDR3 transcript variants expressed by circulating neutrophils, monocytes and T cells from individuals I, III and IV** Diversity tree plots visualizing the relative abundance of TCRβ CDR3 transcript variants expressed by circulating CD15^+^ neutrophils, CD14^+^ monocytes and CD3^+^ T cells. Three healthy individuals (I, III and IV) are shown whose leukocyte subpopulations were purified from a single blood draw. Each spot in a plot represents a rearranged TCRβ transcript that encodes a unique TCRβ CDR3 sequence. It is defined by a unique color and its area is proportional to the relative transcript frequency. A CDR3 sequence is considered unique if it represents a nonredundant fragment of amino acids, which is in a stop-codon-free reading frame and contains both translated conserved V_β_ and J_β_ motifs. The position of each spot within the plot area is defined according to V_β_ usage (x-axis: V_β_1→V_β_i) and J_β_ usage (y-axis: J_β_1→J_β_i). Strikingly large spots reflect the bias of the underlying immune repertoire. Each plot has a distinct color code. Total numbers of identified nonredundant TCRβ CDR3 sequence variants are indicated for each diversity tree plot. CDR3, complementarity determining region 3. *Sample contained ∼4% T cells which may (in part) account for the strikingly high number of TCRβ variants

Supplementary Figure S10 **Pairwise interlineage comparisons of the TCRβ repertoire transcriptomes identified for individuals I, II, III and V, respectively** Pairwise interlineage comparisons of the TCRβ repertoire transcriptomes of human peripheral blood neutrophils (CD15, purple), monocytes (CD14, green) and T cells (CD3, grey), respectively, shown for the healthy individuals I, II, III and V. For each lineage the relative proportions of unique TCRβ CDR3 variants that are also expressed by the respective other lineages are shown in the circle graphs.

Supplementary Figure S11 **Quantitative effect of interlineage cross-contamination on interlineage repertoire sharing** Proportional relationship between interlineage cross-contamination and interlineage repertoire sharing. To quantitate the impact of interlineage cross-contamination on our immune repertoire sequencing approach, CD15^+^ cells from two individuals [donor IV (black), donor V (blue)] were mixed with known numbers of CD3^+^ input cells (1%, 3% and 10%) and subjected to ARM-PCR based high-throughput sequencing. Shown are the resulting mean percentages (**A**) and total numbers (**B**) of shared unique TCRβ CDR3 sequences in the CD3→CD15 cell mixtures. The results of the two independent experiments reveal a linear relationship between the frequency of shared CDR3 variants and the number of contaminating cells. Note that the quantitative effect of CD3^+^ cell input cells on the neutrophil TCRβ transcriptome is detectable when the proportion of CD3^+^ input cells is ≥1%. A detailed synopsis of the 50 most frequently expressed CDR3 variants identified in this experiment is shown in Figure S14A and B.

Supplementary Figure S12 **Proportional relationship between template copy numbers and the number of sequence reads** Proportional relationship between template copy numbers and the number of sequence reads. To test whether ARM-PCR based high-throughput sequencing maintains quantitative proportions in transcriptome analyses, T cell cDNA from transgenic OT II.Rag1^−/−^ and CBir1.Rag1^−/−^ mice that express defined dominant TCRβ clonotypes was obtained. cDNAs were diluted in ten-fold steps into T cell cDNA from normal C57BL/6 mice to create four distinct cDNA pools (I–IV). TCRβ CDR3 regions of the cDNA mixes were then subjected to ARM-PCR based high-throughput sequencing. Amplicons of each pool were tagged with a unique barcode sequence. The transcript frequencies that were determined by sequencing for the TCRβ clonotype ASSLGGESQNTLY (expressed by OT-II.Rag^−/−^ mice) were 341, 26, 6, and 0, respectively (red). The value 0 is not shown in the log-scale plot. The copy numbers assessed for the CBir1.Rag^−/−^-specific TCRβ clonotype (ASTSGGLSYEQY) were 4153, 408, 41 and 6, respectively (blue). Note the consistent proportional relationship between the template copy number and the number of obtained sequence reads in both independent experiments.

Supplementary Figure S13 **No correlation between interlineage cross-contamination and interlineage CDR3 repertoire sharing** No correlation between interlineage cross-contamination and the percentage of TCRβ CDR3 variants that are shared by any two leukocyte lineages. Shown are pairwise comparisons of interlineage cross-contamination (%) and CDR3 repertoire sharing (%) for the CD15^+^, CD14^+^ and CD3^+^ populations, respectively, purified from individuals I–V.

Supplementary Figure S14 **Quantitative effect of CD3**^+^**→CD15**^+^**and CD3**^+^**→CD3**^+^**cell crosscontamination on TCRβ CDR3 repertoire profiles** Quantitative effect of CD3^+^**→**CD15^+^ (**A and B**) and CD3^+^**→**CD3^+^ (**C**) cross-contamination on TCRβ CDR3 repertoire profiles. CD3^+^**→**CD15^+^ cross-contamination. CD15^+^ cells from two individuals (donor IV, donor V) were mixed with known numbers of their own CD3^+^ cells (1%, 3% and 10%) and subjected to ARM-PCR based high-throughput sequencing. The 50 most frequently expressed CDR3 variants expressed by donor IV (**A**) and V (**B**) are listed for all samples in descending order. Numbers designate TCRβ CDR3 transcript frequencies. Red lines track the positions of representative CD3^+^ specific CDR3 sequences in the cell mixes. Note that the transcript frequencies are proportional to the amount of input CD3^+^ cells and that CD3^+^ specific CDR3 sequences are already detectable in CD15^+^ populations at a cross-contamination rate of 1%. **C**. CD3^+^**→** CD3^+^ cross-contamination. CD3^+^ cells from donor III were mixed with CD3^+^ cells from donor II at the indicated percentages. Blue shaded CDR3 sequences are only expressed by donor II, orange sequences are specifically expressed by donor III. The positions of the most frequently expressed CDR3 sequences in donor II (blue) are are shown in the frequency lists of the mixed cell populations. Arrows demonstrate that the emergence of donor II-specific CDR3 sequences in the mixed cell populations is proportional to the percentage of cross-contamination (for clarity only shown for selected CDR3 variants). The designated color codes are shown in the individual figure panels.

Supplementary Figure S15 **Interlineage discrepancies in the order of transcript frequency between shared CDR3 sequences** Synopsis of the 50 most frequently expressed TCRβ CDR3 variants in each lineage shown for donors I–V (**A–E**). Numbers designate transcript frequencies. CDR3 variants that are shared between distinct leukocyte lineages are highlighted by identical colors. Interlineage discrepancies in the order of transcript frequency between shared CDR3 sequences are highlighted by lines (for clarity shown only for a few selected CDR3 variants). The designated color codes are shown in the individual figure panels. **F**. Synopsis of 10 shared TCRβ CDR3 variants in each lineage (CD15^+^, CD14^+^, M1 macrophages and CD3^+^) from donor V. TRBC1 and TRBC2 denote the constant chain use of each cell lineage for the respective CDR3 sequence. The respective 10 sequences of donor V are designated with an asterisk (*) in Figure S15E.

Supplementary Figure S16 **V_β_/J_β_ gene usage (3D-Maps) of the TCRβ repertoires expressed by human monocytes and IFNγ-polarized M1 macrophages** V_β_/J_β_ gene usage of the TCRβ CDR3 repertoires expressed by CD14^+^ monocytes and IFNγ-polarized M1 macrophages from individuals I, IV and V, respectively. 3D-Maps as in Figure S4). hTRBVx and hTRBJy denote individual human TCR variable V_β_-genes and J_β_-genes, respectively, according to the nomenclature used by the IMGT/GENE database (http://www.imgt.org/IMGTindex/IMGTgene-db.html). x-axis: V_β_ gene; y- axis: J_β_ gene; z-axis: expression frequency.

Supplementary Figure S17 **Monocyte vs. M1-macrophage TCRβ trancriptomes (donors IV and V)** Comparison of the complete TCRβ transcriptomes of peripheral blood CD14^+^ monocytes and IFNγ-polarized M1 macrophages from individuals IV (**A**) and V (**B**) Total numbers of distinct TCRβ CDR3 sequence variants that are expressed before and after monocyte-to-macrophage differentiation are indicated. CD14^+^ monocytes were purified from peripheral blood and an aliquot was differentiated into M1 polarized macrophages in the presence of IFNγ for 6 days. The diversity plots provide a global view of the repertoire reprogramming that occurs during monocyte differentiation into proinflammatory M1 macrophages. The 10 most frequently expressed CDR3 variants in each cell population (bottom) are shown in detail. Monocyte-specific and macrophage-specific CDR3 sequences are indicated in distinct colors. Purple sequences represent shared CDR3 variants.

Supplementary Figure S18 **Test-retest reliability of ARM-PCR based TCR transcriptome sequencing** High test-retest reliability of ARM-PCR based TCR transcriptome sequencing. Two identical aliquots of the same input sample were subjected to ARM-PCR reactions using V/J primers specific for all human TCR chains and then sequenced in one and the same sequencing run. The correlation coefficients between both input samples were 0.99 for distinct V-J combinations (left) and 0.93 for the 10.000 most abundant CDR3 sequences identified (right).

Supplementary Table S1 **CDR3 sequence reads for human circulating neutrophils, monocytes, T cells and monocyte-derived M1 macrophages**

Supplementary Table S2 **Unique TCRβ CDR3 variants shared between neutrophils, monocytes and T cells in each individual**

Supplementary Table S3 **Shared CDR3 and interlineage cross-contamination**

Supplementary Table S4 **Sharing of TCRβ CDR3 sequences expressed by all three leukocyte lineages (“trilineage expression”)**

Supplementary Table S5 **TCRβ CDR3 sequences exclusive to one lineage and simultaneously shared between all donors (“lineage-specific AND public”)**

Supplementary Table S6 **Shared CDR3 sequences between CD14 monocytes and M1 macrophages**
