# Supplementary figures and images for: Trilineage Sequencing Reveals Complex TCRβ Transcriptomes in Neutrophils and Monocytes Alongside T Cells
Source: Genomics Proteomics Bioinformatics. 2021 Mar 2;19(6):926–36. doi: 10.1016/j.gpb.2019.02.004 (PMC9402791; doi:10.1016/j.gpb.2019.02.004)

A

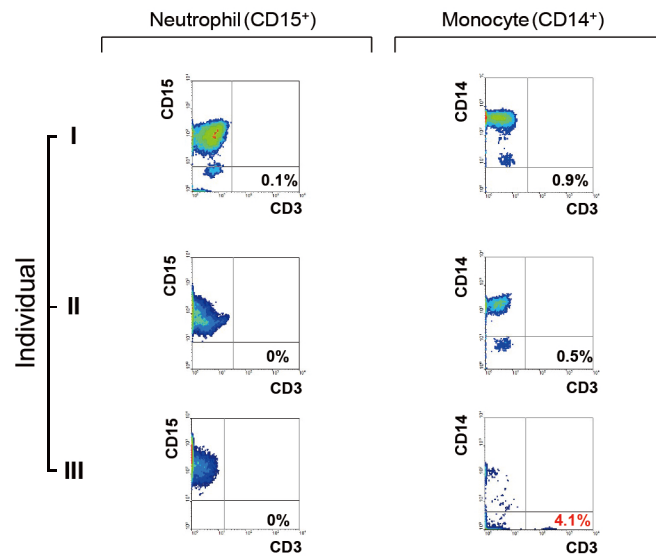

B

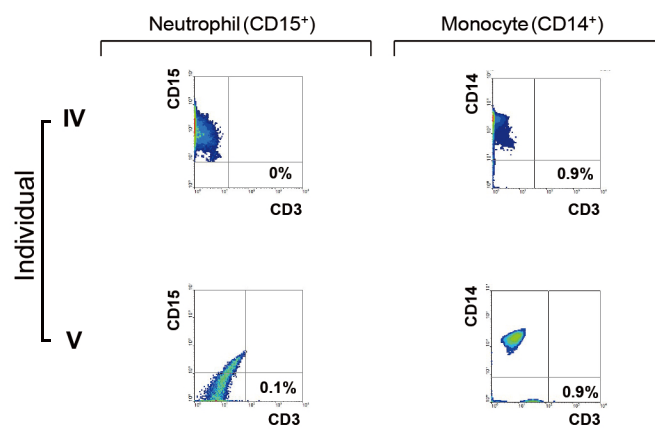

Supplement: Supplementary Figure S1 — Individual cell purities of human peripheral blood CD15+ neutrophils and CD14+ monocytes Flow cytometry demonstrates individual cell purities of human peripheral blood CD15+ neutrophils and CD14+ monocytes isolated from donors I–V. The respective percentage of CD3+ panT cells are indicated. [file mmc1.pdf]

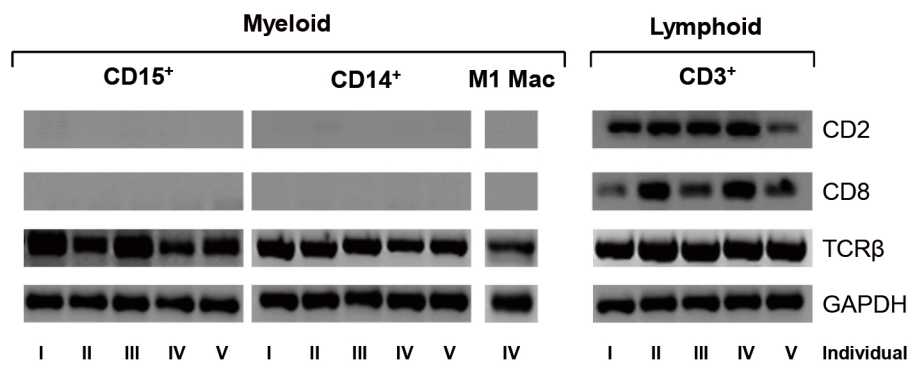

Supplement: Supplementary Figure S2 — RT-PCR expression profiling of the TCRβ chain and T lymphoid markers in purified CD15+neutrophils, CD14+monocytes, IFNγ macrophages and CD3+T cells The human peripheral blood CD15+ neutrophils, CD14+ monocytes and CD3+ T cells isolated from donors I–V constitutively express the genes for the TCRβ constant chain. Purity of the neutrophil and monocyte samples is demonstrated by the absence of the T cell marker CD2 and CD8, respectively. In addition, the RT-PCR results of IFNγ polarized M1 macrophages are shown for one representative individual (donor IV). Peripheral blood monocytes were isolated by CD14-MACS and differentiation into Th1 (IFNγ) polarized MΦ was induced for 6 days. GAPDH is shown as reference. GAPDH: Glyceraldehyde 3-phosphate dehydrogenase. [file mmc2.pdf]

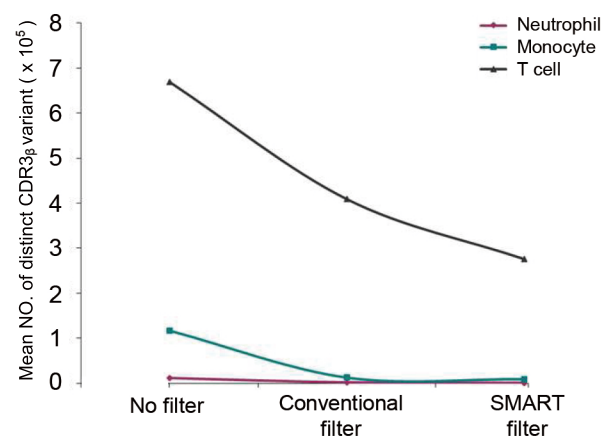

Supplement: Supplementary Figure S3 — High-stringency data analysis of the trilineage TCRβ transcriptomes using the five-step SMART filtering strategy High-stringency data analysis of the TCRβ transcriptomes in human peripheral blood neutrophils, monocytes and T cells using the five-step SMART filtering strategy. The SMART filter eliminates sequencing artifacts at the following quality control checkpoints: sequencing errors, mosaic formation, PCR amplification errors, reference sequence errors and frequency threshold. Note the significant reduction of erroneous TCRβ CDR3 reads. Unfiltered sequence reads and paired-end filtering (conventional data filter) are shown for comparison. The frequency threshold filter was set >1 which excludes single copy CDR3 transcripts. [file mmc3.pdf]

**A Individual I**

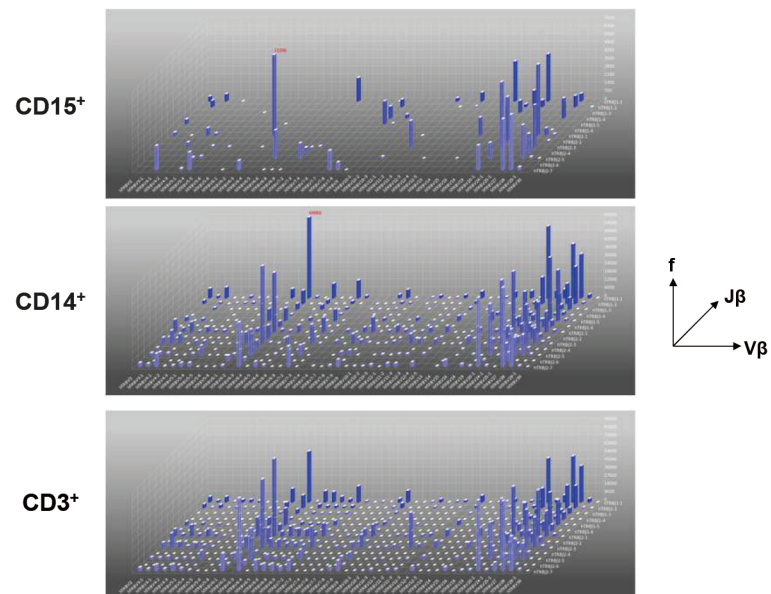

**B Individual II**

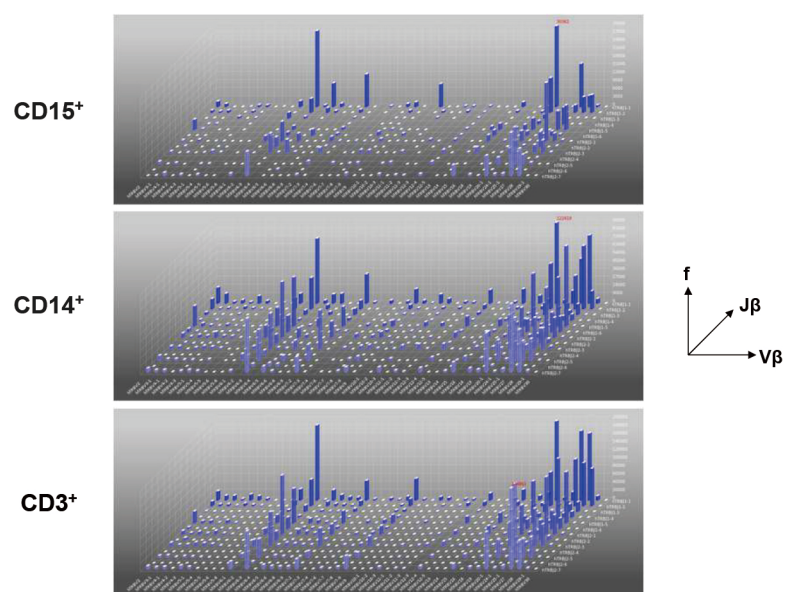

**C Individual III**

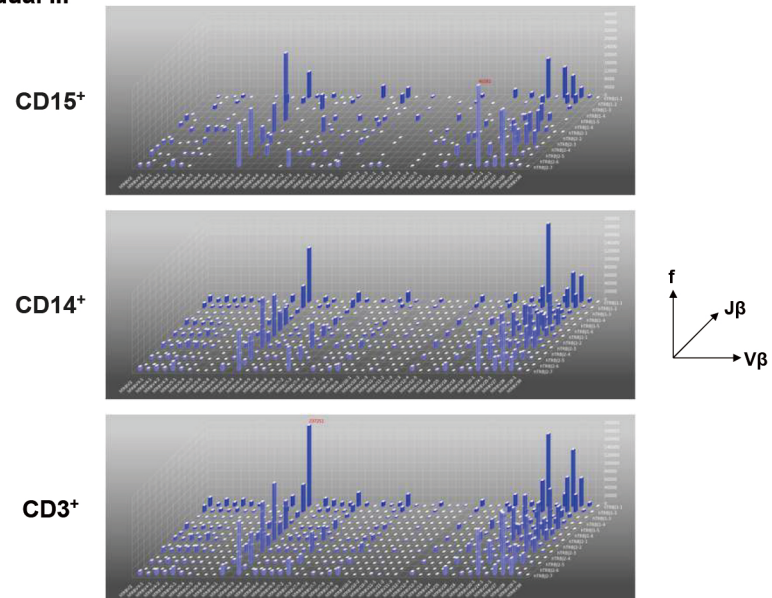

**D Individual IV**

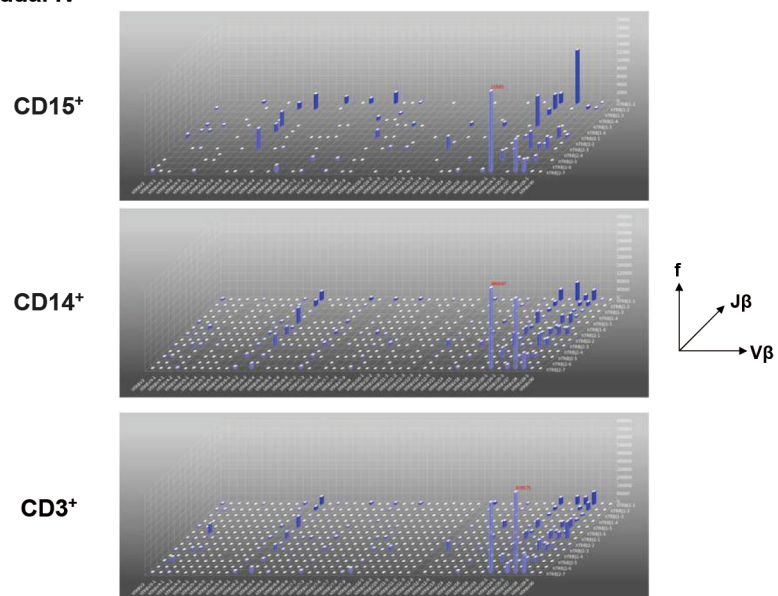

### E Individual V

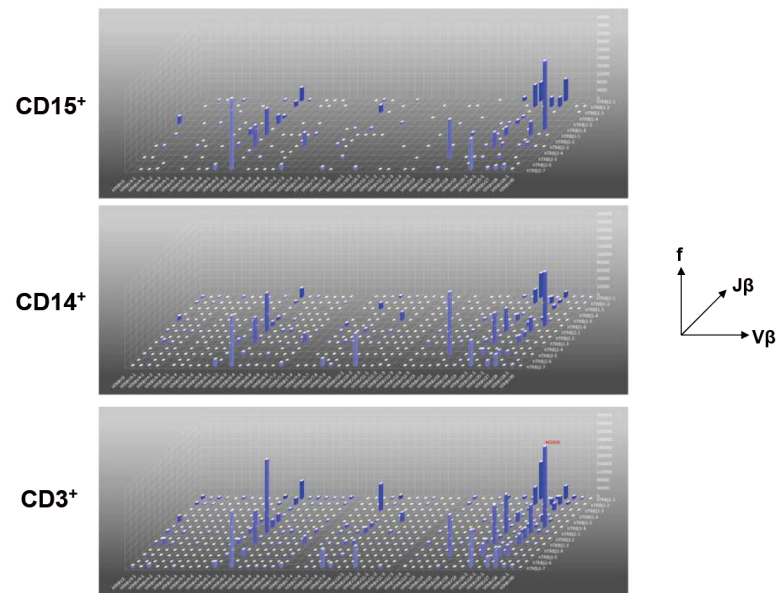

Supplement: Supplementary Figure S4 — Vβ/Jβ gene usage (3D-Maps) of the TCRβ repertoires expressed by human neutrophils, monocytes and T cells Vβ/Jβ gene usage of the TCRβ CDR3 repertoires expressed by human neutrophils (CD15), monocytes (CD14) and T cells (CD3). 3D-Maps in which each expressed Vβx/Jβy combination is represented by a bar whose height corresponds to its expression frequency are shown for individuals I–V (A–E). hTRBVx and hTRBJy denote individual human TCR variable Vβ-genes and Jβ-genes, respectively, according to the nomenclature used by the IMGT/GENE database (http://www.imgt.org/IMGTindex/IMGTgene-db.html). x-axis: Vβ gene; y- axis: Jβ gene; z-axis: expression frequency. [file mmc4.pdf]

## A Individual I

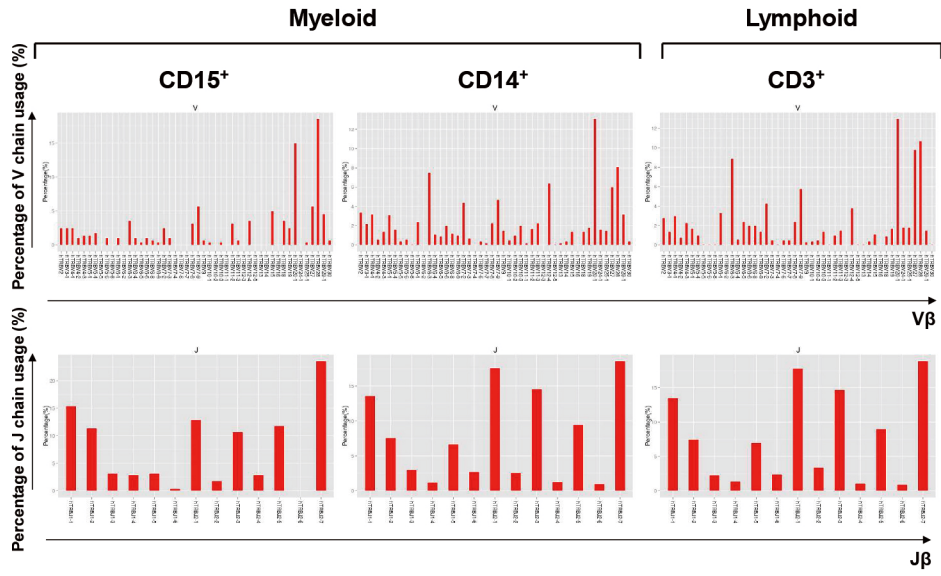

## B Individual II

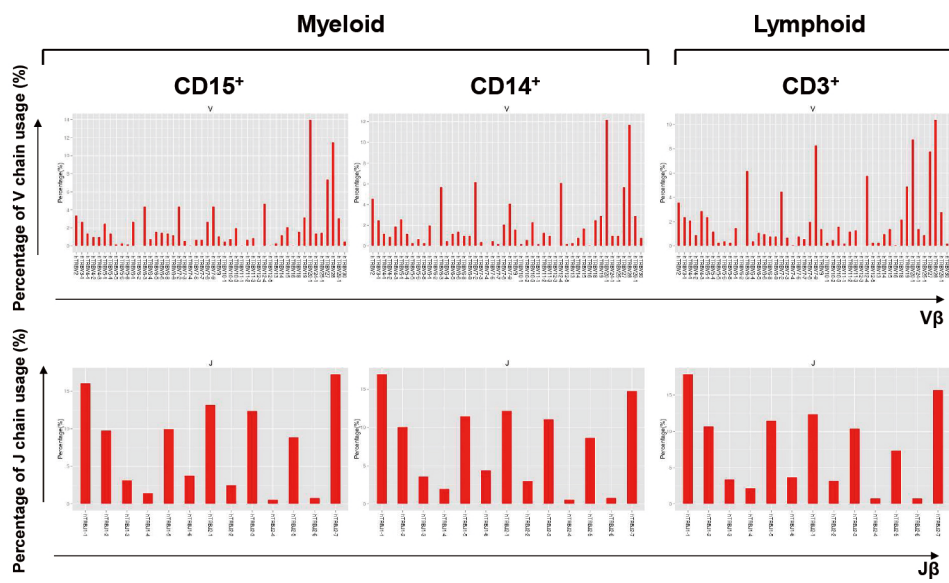

### C Individual III

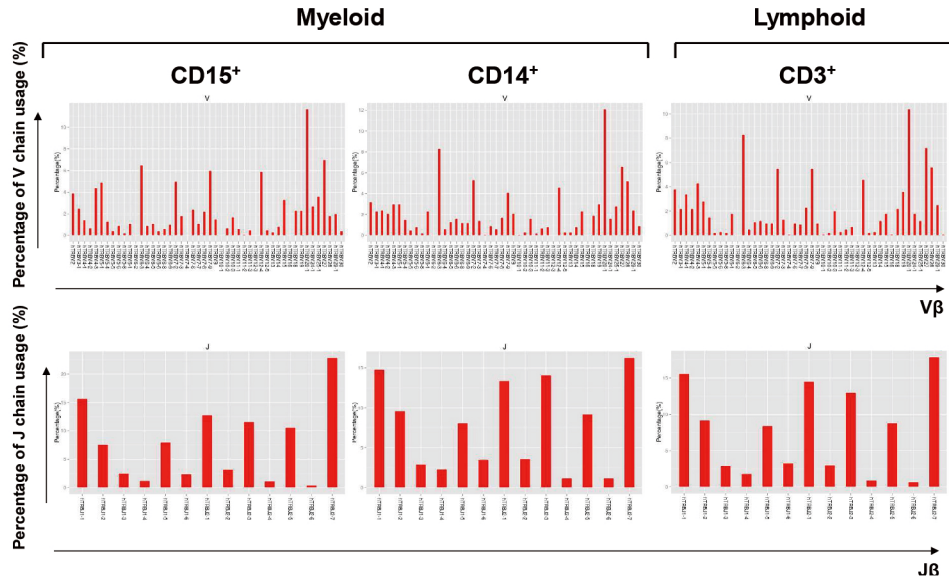

### D Individual IV

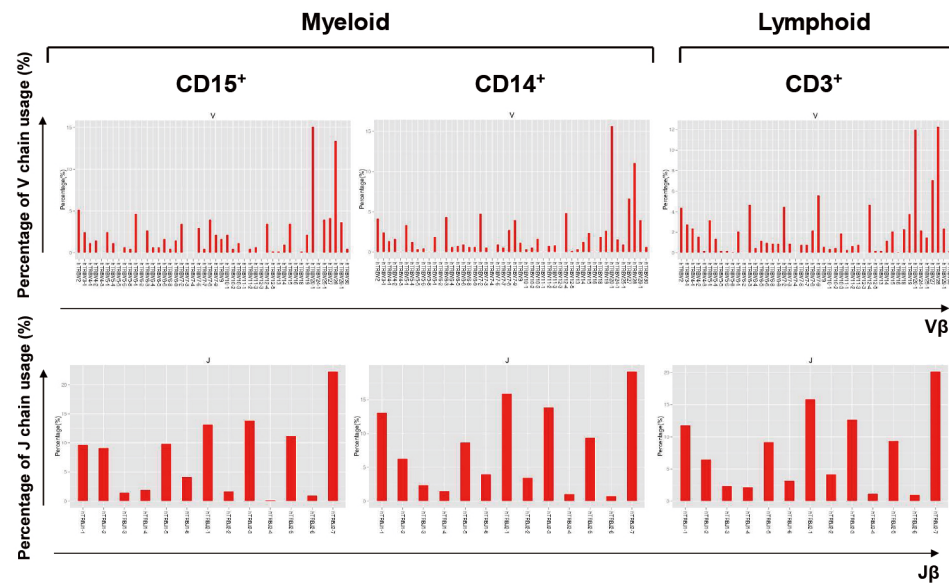

# **E Individual V**

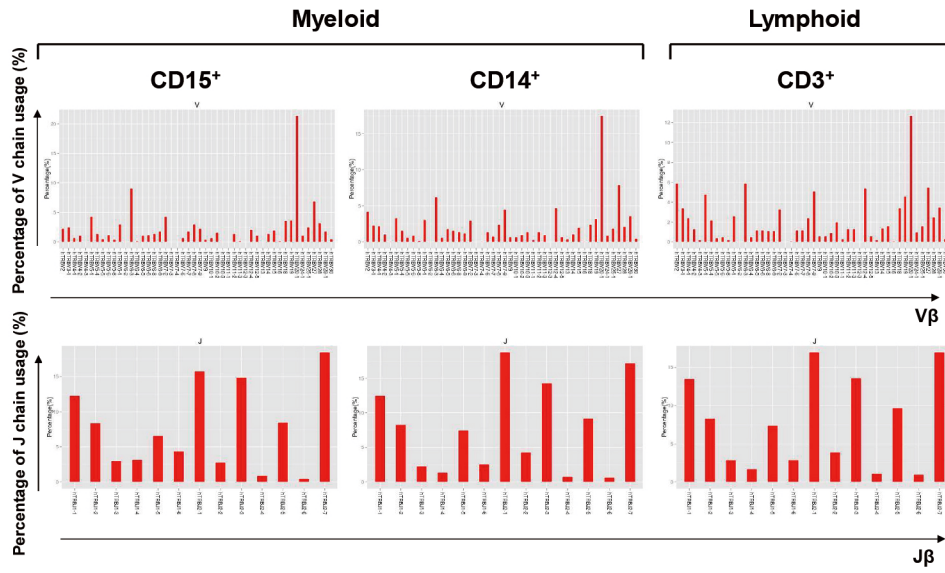

Supplement: Supplementary Figure S5 — Vβ/Jβ gene usage (2D-plots) Vβ/Jβ gene usage of human peripheral blood neutrophils (CD15), monocytes (CD14) and T cells (CD3), respectively. The 2D-plots show the relative usage of the Vβ (top) and Jβ (bottom) genes for each individual (A–E). The results are normalized to eliminate copy number biases. Note that the three leukocyte lineages do not differ in their Vβ and Jβ gene usage. X-axis: Vβ gene/Jβ gene; y-axis: percentage of used Vβ/Jβ genes. [file mmc5.pdf]

## A Individual I

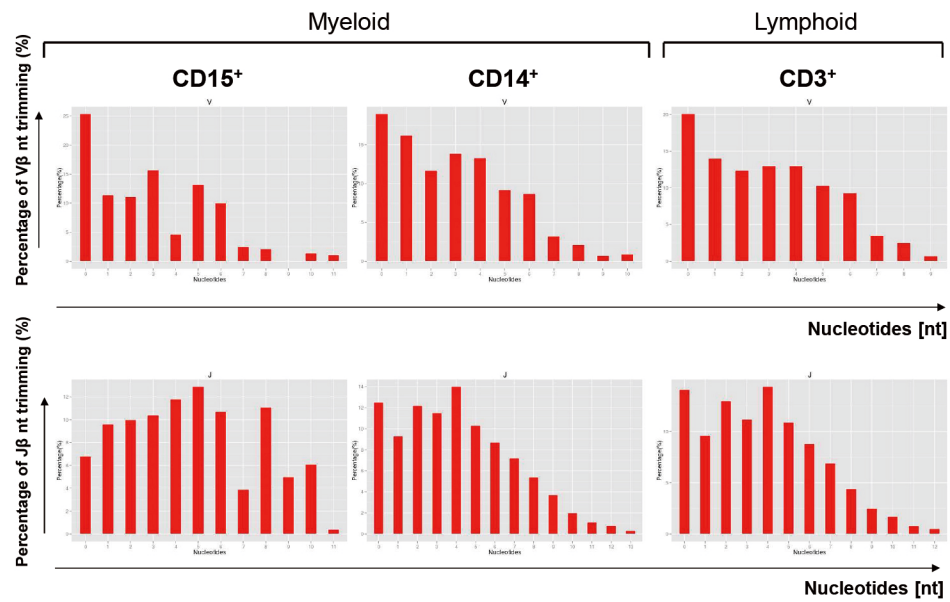

## B Individual II

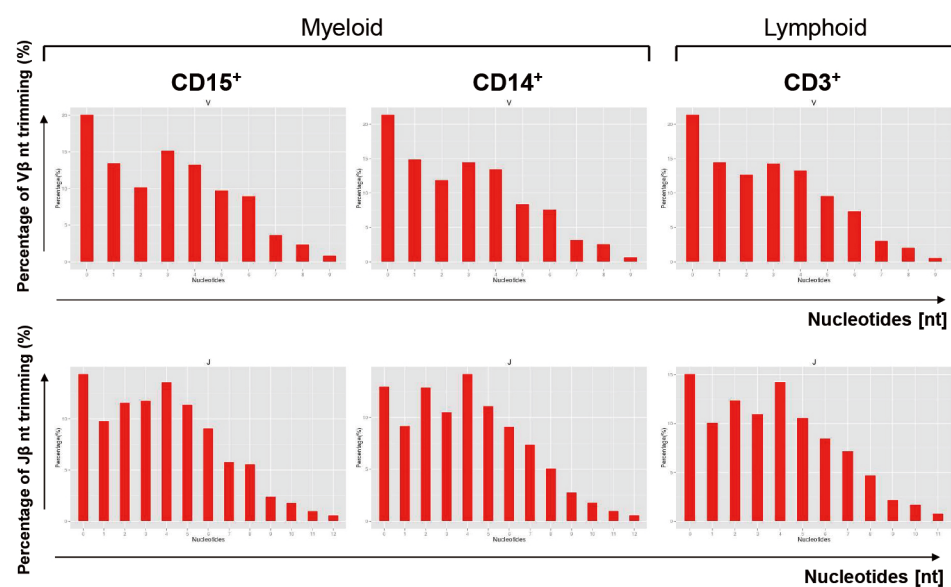

**C Individual III**

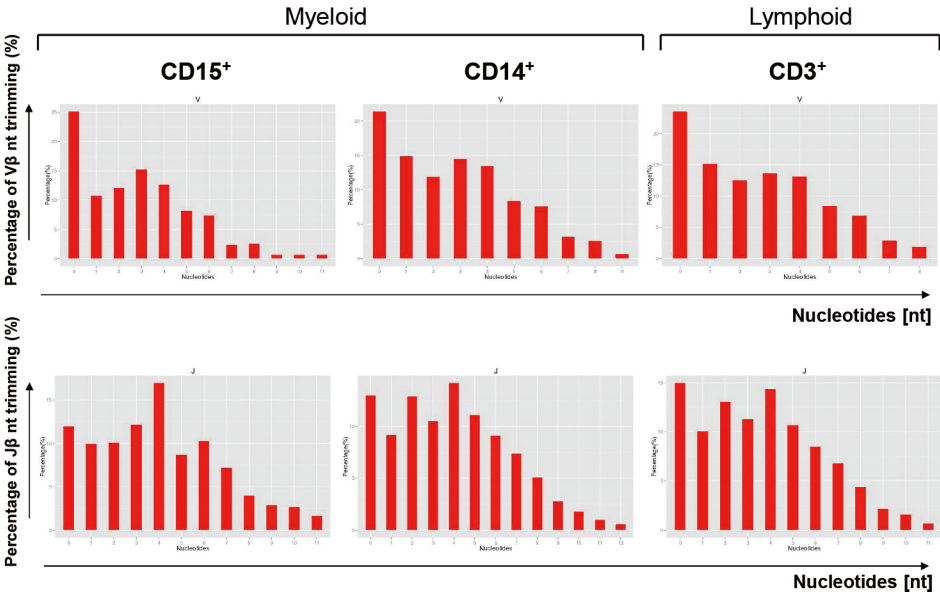

**D Individual IV**

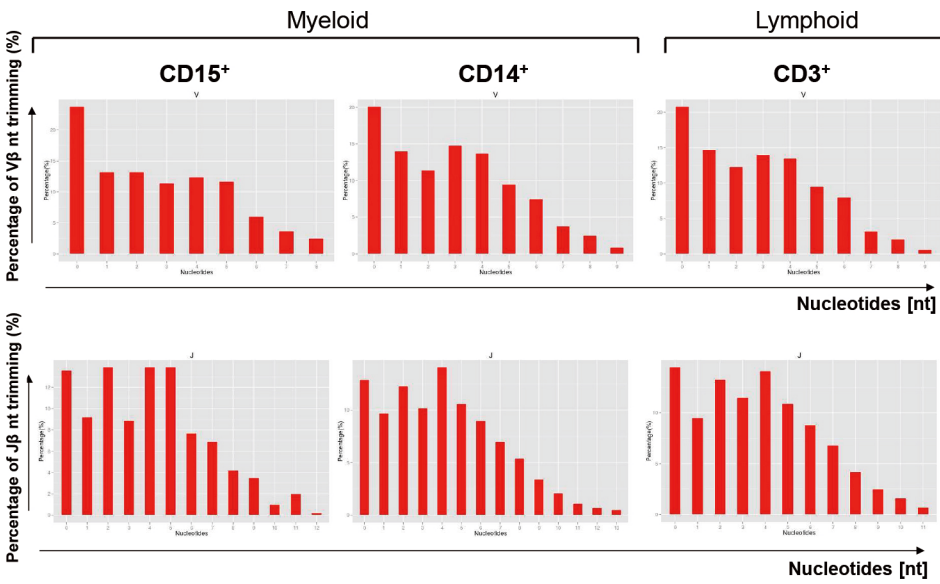

**E Individual V**

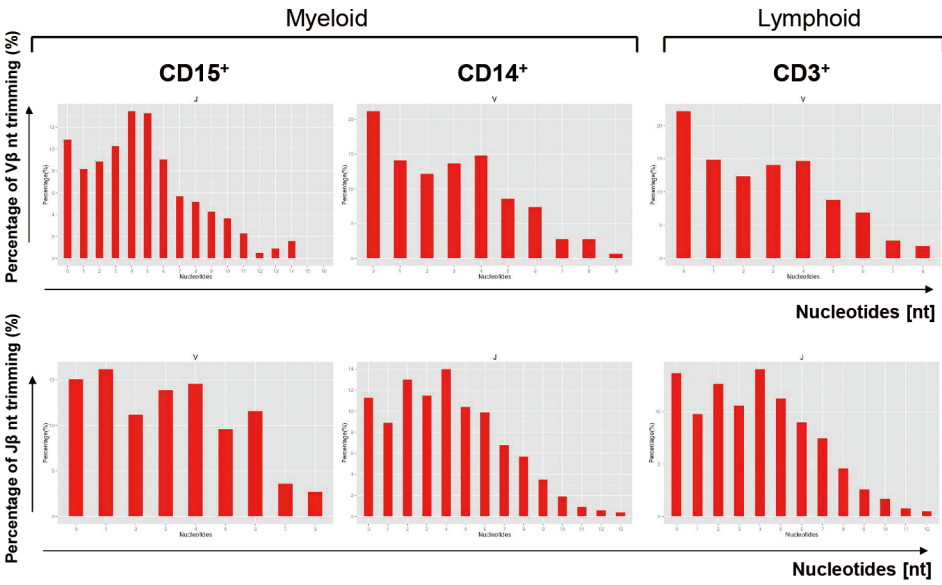

Supplement: Supplementary Figure S6 — Vβ/Jβ trimming Vβ and Jβ nucleotide trimming of the CDR3 sequences expressed by human peripheral blood neutrophils (CD15), monocytes (CD14) and T cells (CD3). Bar graphs show the relative distribution of nucleotide trimming at the coding ends of the germline Vβ (top) and Jβ gene segments (bottom, individuals I–V). The results are normalized to eliminate copy number biases. X-axis: number of trimmed nucleotides [nt]; y-axis: percentage of CDR3 sequences per number of trimmed nucleotides. [file mmc6.pdf]

**A Individual I**

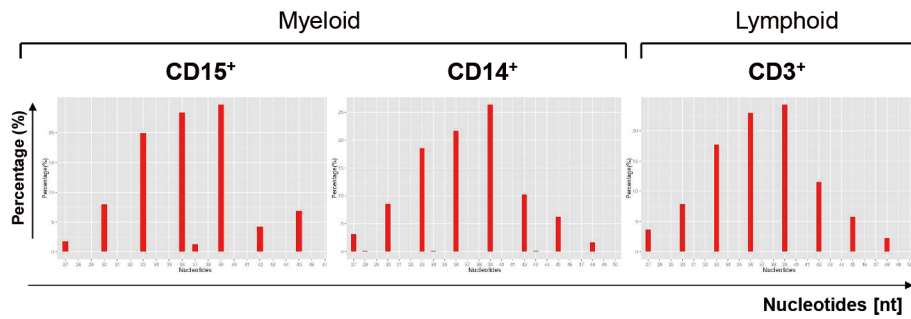

### B Individual II

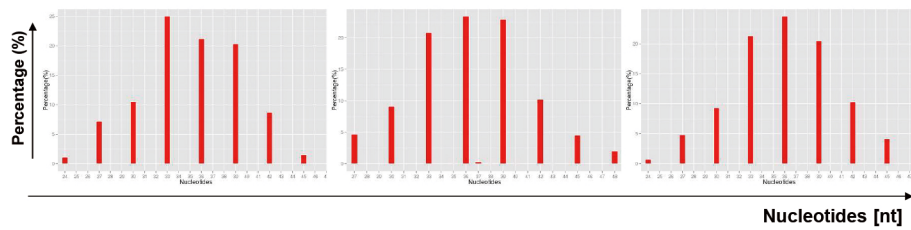

### C Individual III

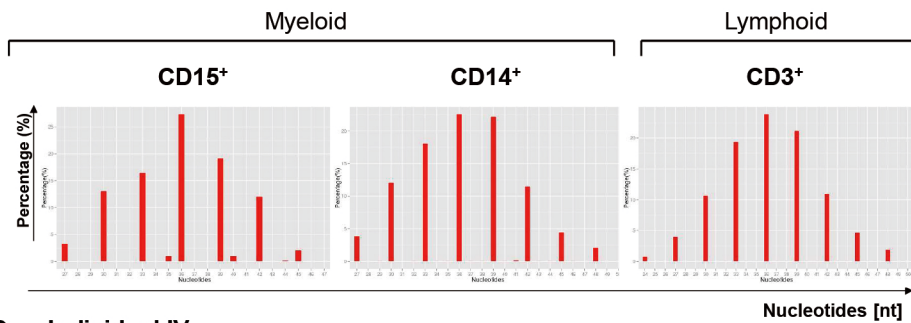

### D Individual IV

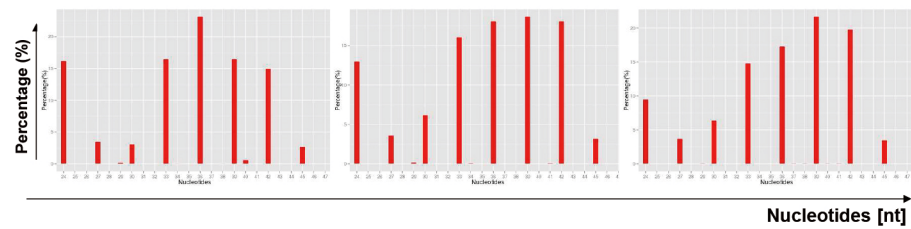

**E Individual V**

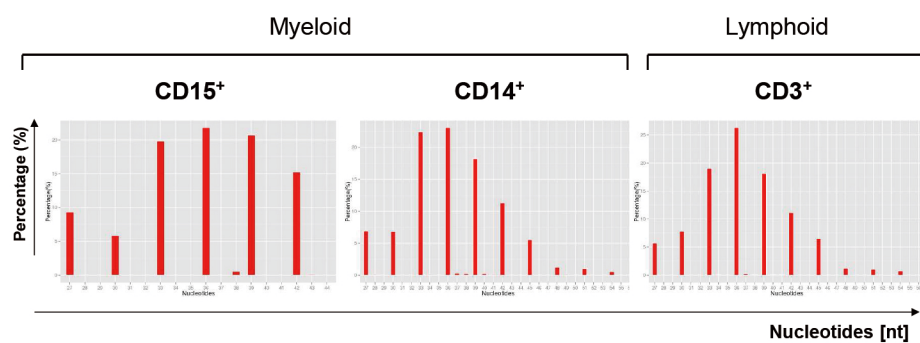

Supplement: Supplementary Figure S7 — Length distribution of expressed TCRβ CDR3 regions Length distribution of the neutrophil (CD15), monocyte (CD14) and T cell (CD3) TCRβ CDR3 regions expressed by individuals I–V. Bar graphs indicate relative percentages of individual CDR3 lengths. Note that most individuals exhibit non-Gaussian length distribution patterns. [file mmc7.pdf]

**A Individual I**

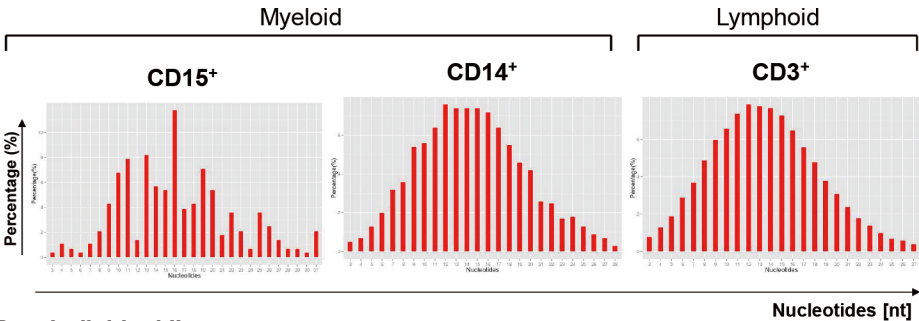

**B Individual II**

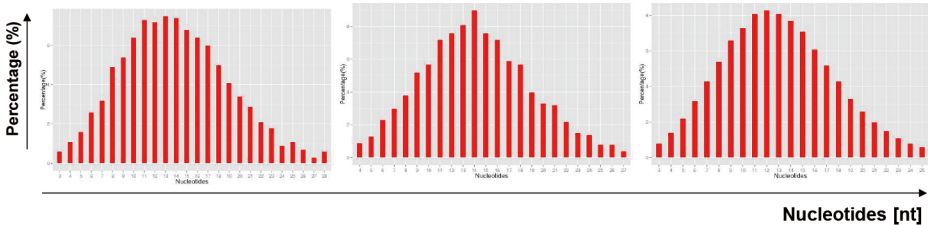

**C Individual III**

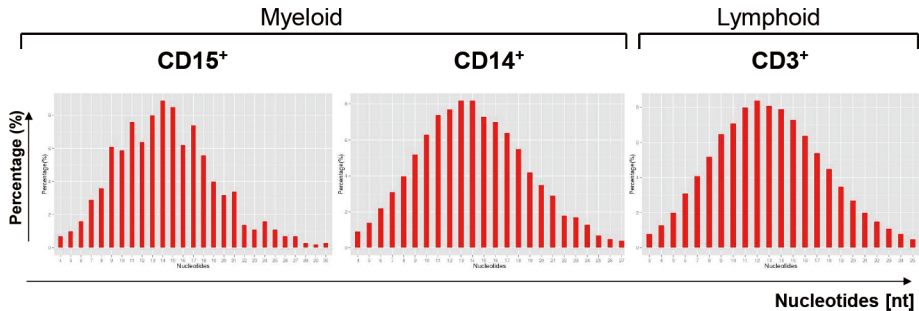

**D Individual IV**

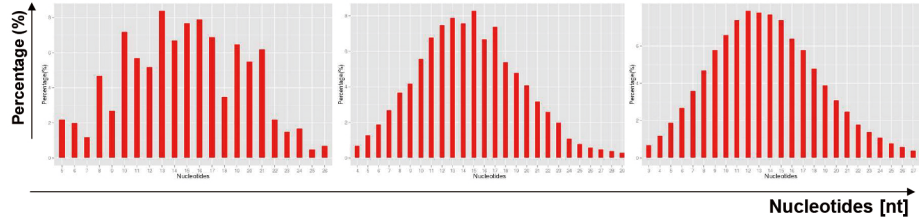

**E Individual V**

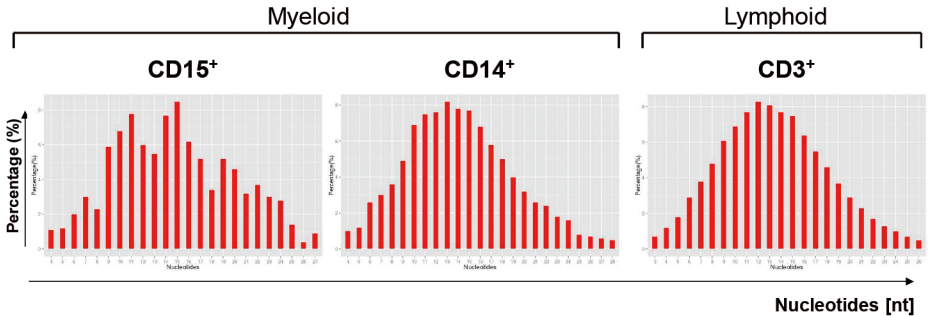

Supplement: Supplementary Figure S8 — Nontemplated nucleotide addition Relative distribution (%) of nontemplated nucleotides added to the CDR3 regions expressed by neutrophils (CD15), monocytes (CD14) and T cells (CD3). Data are corrected for copy number biases. Note the non-Gaussian patterns in neutrophils, whereas Gaussian profiles were observed in most of the monocyte and T cell populations. [file mmc8.pdf]

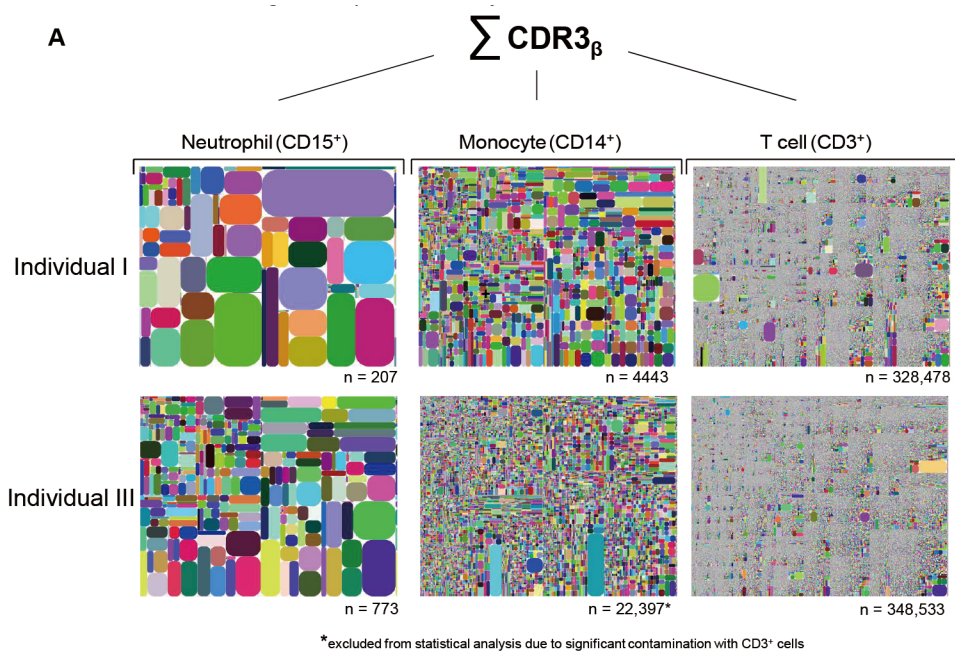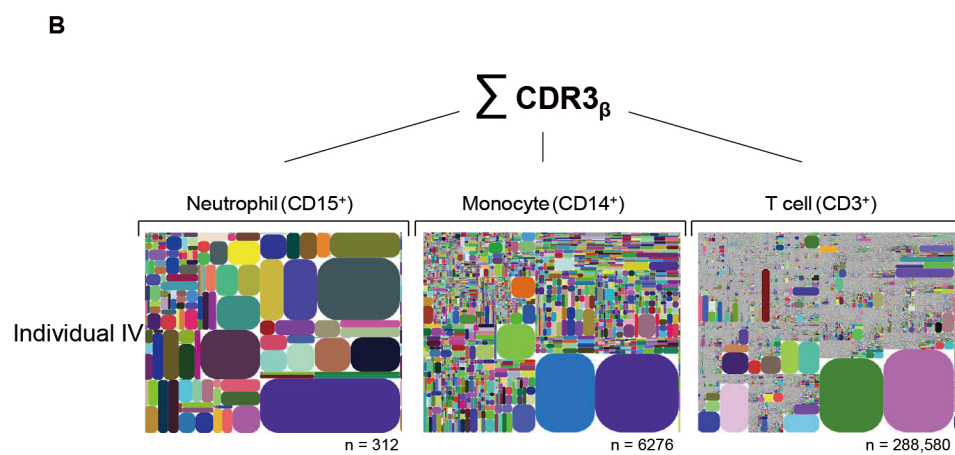

Supplement: Supplementary Figure S9 — Diversity tree plots of TCRβ CDR3 transcript variants expressed by circulating neutrophils, monocytes and T cells from individuals I, III and IV Diversity tree plots visualizing the relative abundance of TCRβ CDR3 transcript variants expressed by circulating CD15+ neutrophils, CD14+ monocytes and CD3+ T cells. Three healthy individuals (I, III and IV) are shown whose leukocyte subpopulations were purified from a single blood draw. Each spot in a plot represents a rearranged TCRβ transcript that encodes a unique TCRβ CDR3 sequence. It is defined by a unique color and its area is proportional to the relative transcript frequency. A CDR3 sequence is considered unique if it represents a nonredundant fragment of amino acids, which is in a stop-codon-free reading frame and contains both translated conserved Vβ and Jβ motifs. The position of each spot within the plot area is defined according to Vβ usage (x-axis: Vβ1→Vβi) and Jβ usage (y-axis: Jβ1→Jβi). Strikingly large spots reflect the bias of the underlying immune repertoire. Each plot has a distinct color code. Total numbers of identified nonredundant TCRβ CDR3 sequence variants are indicated for each diversity tree plot. CDR3, complementarity determining region 3. *Sample contained ∼4% T cells which may (in part) account for the strikingly high number of TCRβ variants [file mmc9.pdf]

**A**

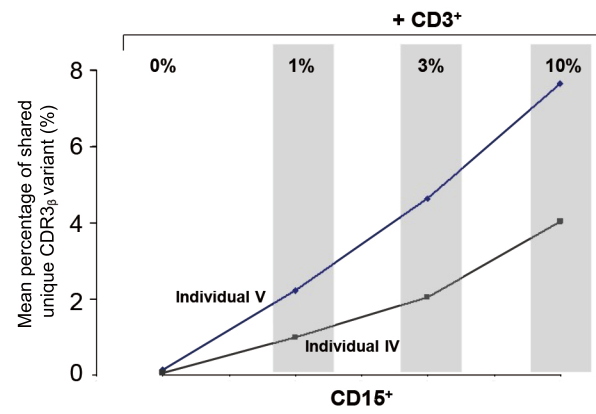

**B**

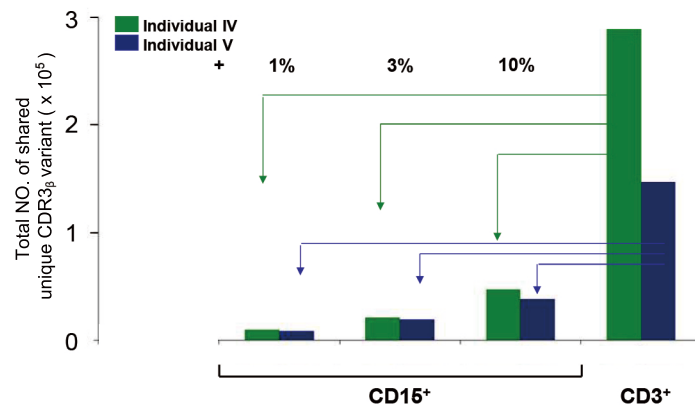

Supplement: Supplementary Figure S11 — Quantitative effect of interlineage cross-contamination on interlineage repertoire sharing Proportional relationship between interlineage cross-contamination and interlineage repertoire sharing. To quantitate the impact of interlineage cross-contamination on our immune repertoire sequencing approach, CD15+ cells from two individuals [donor IV (black), donor V (blue)] were mixed with known numbers of CD3+ input cells (1%, 3% and 10%) and subjected to ARM-PCR based high-throughput sequencing. Shown are the resulting mean percentages (A) and total numbers (B) of shared unique TCRβ CDR3 sequences in the CD3→CD15 cell mixtures. The results of the two independent experiments reveal a linear relationship between the frequency of shared CDR3 variants and the number of contaminating cells. Note that the quantitative effect of CD3+ cell input cells on the neutrophil TCRβ transcriptome is detectable when the proportion of CD3+ input cells is ≥1%. A detailed synopsis of the 50 most frequently expressed CDR3 variants identified in this experiment is shown in Figure S14A and B. [file mmc11.pdf]

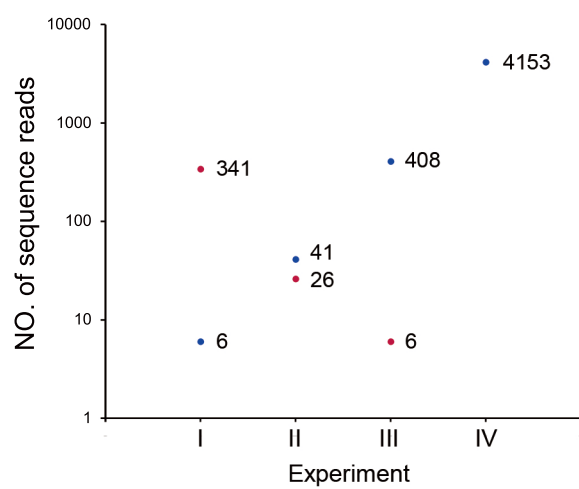

Supplement: Supplementary Figure S12 — Proportional relationship between template copy numbers and the number of sequence reads Proportional relationship between template copy numbers and the number of sequence reads. To test whether ARM-PCR based high-throughput sequencing maintains quantitative proportions in transcriptome analyses, T cell cDNA from transgenic OT II.Rag1−/− and CBir1.Rag1−/− mice that express defined dominant TCRβ clonotypes was obtained. cDNAs were diluted in ten-fold steps into T cell cDNA from normal C57BL/6 mice to create four distinct cDNA pools (I–IV). TCRβ CDR3 regions of the cDNA mixes were then subjected to ARM-PCR based high-throughput sequencing. Amplicons of each pool were tagged with a unique barcode sequence. The transcript frequencies that were determined by sequencing for the TCRβ clonotype ASSLGGESQNTLY (expressed by OT-II.Rag−/− mice) were 341, 26, 6, and 0, respectively (red). The value 0 is not shown in the log-scale plot. The copy numbers assessed for the CBir1.Rag−/−-specific TCRβ clonotype (ASTSGGLSYEQY) were 4153, 408, 41 and 6, respectively (blue). Note the consistent proportional relationship between the template copy number and the number of obtained sequence reads in both independent experiments. [file mmc12.pdf]

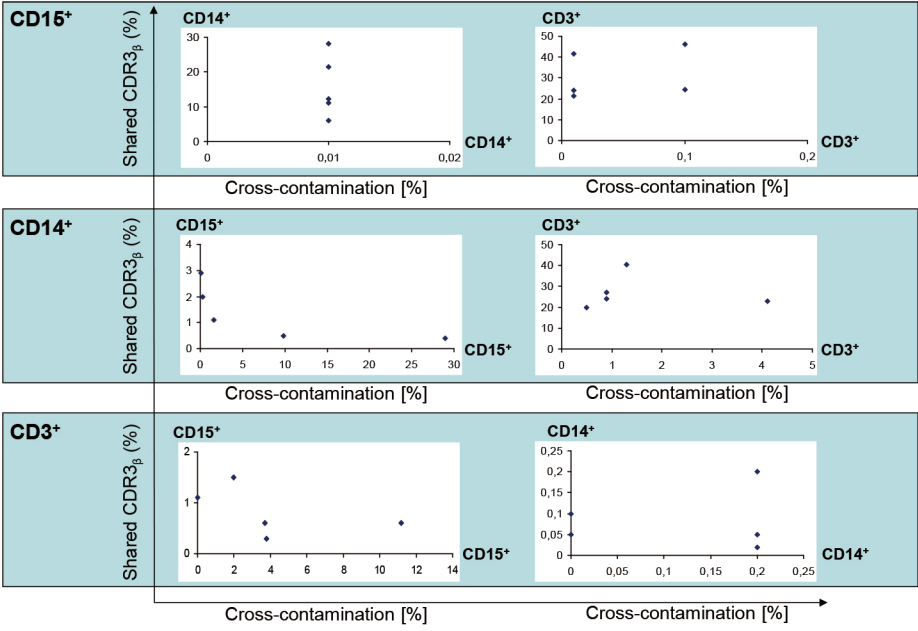

Supplement: Supplementary Figure S13 — No correlation between interlineage cross-contamination and interlineage CDR3 repertoire sharing No correlation between interlineage cross-contamination and the percentage of TCRβ CDR3 variants that are shared by any two leukocyte lineages. Shown are pairwise comparisons of interlineage cross-contamination (%) and CDR3 repertoire sharing (%) for the CD15+, CD14+ and CD3+ populations, respectively, purified from individuals I–V. [file mmc13.pdf]

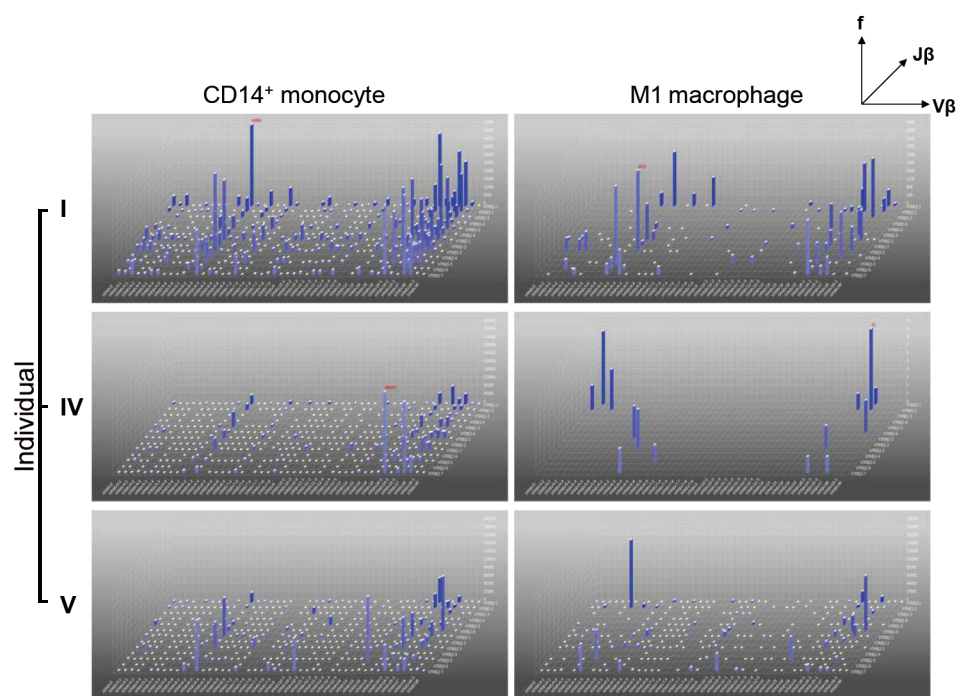

Supplement: Supplementary Figure S16 — Vβ/Jβ gene usage (3D-Maps) of the TCRβ repertoires expressed by human monocytes and IFNγ-polarized M1 macrophages Vβ/Jβ gene usage of the TCRβ CDR3 repertoires expressed by CD14+ monocytes and IFNγ-polarized M1 macrophages from individuals I, IV and V, respectively. 3D-Maps as in Figure S4). hTRBVx and hTRBJy denote individual human TCR variable Vβ-genes and Jβ-genes, respectively, according to the nomenclature used by the IMGT/GENE database (http://www.imgt.org/IMGTindex/IMGTgene-db.html). x-axis: Vβ gene; y- axis: Jβ gene; z-axis: expression frequency. [file mmc16.pdf]

# **A Individual IV**

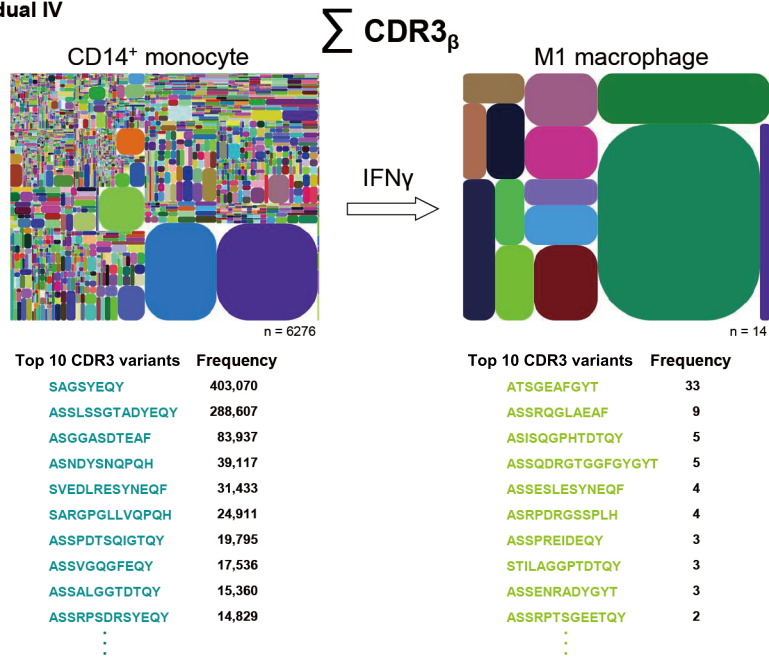

# **B Individual V**

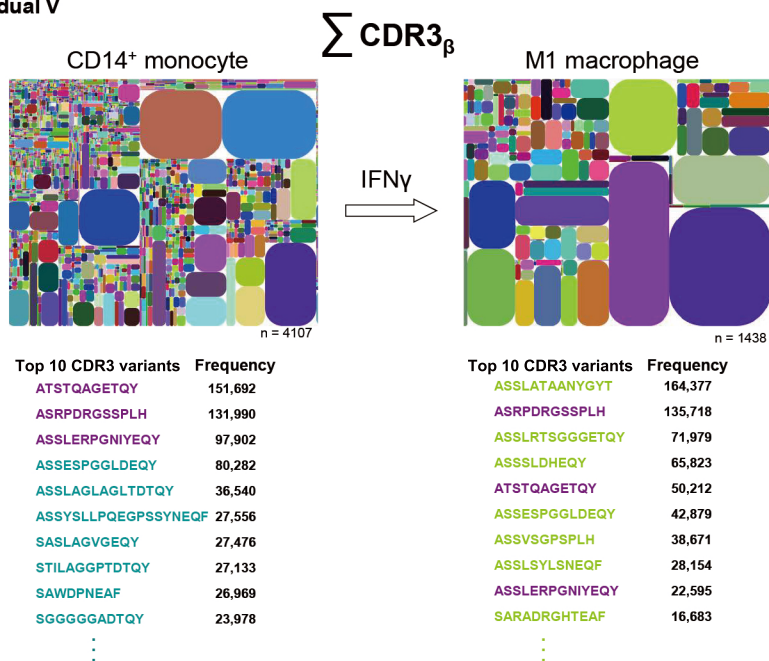

Supplement: Supplementary Figure S17 — Monocyte vs. M1-macrophage TCRβ trancriptomes (donors IV and V) Comparison of the complete TCRβ transcriptomes of peripheral blood CD14+ monocytes and IFNγ-polarized M1 macrophages from individuals IV (A) and V (B) Total numbers of distinct TCRβ CDR3 sequence variants that are expressed before and after monocyte-to-macrophage differentiation are indicated. CD14+ monocytes were purified from peripheral blood and an aliquot was differentiated into M1 polarized macrophages in the presence of IFNγ for 6 days. The diversity plots provide a global view of the repertoire reprogramming that occurs during monocyte differentiation into proinflammatory M1 macrophages. The 10 most frequently expressed CDR3 variants in each cell population (bottom) are shown in detail. Monocyte-specific and macrophage-specific CDR3 sequences are indicated in distinct colors. Purple sequences represent shared CDR3 variants. [file mmc17.pdf]

**A V-J combination**

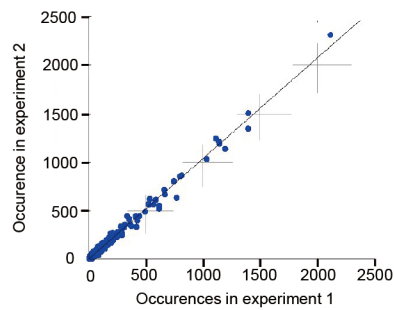

**B 10,000 most frequent CDR3<sub>β</sub> variant**

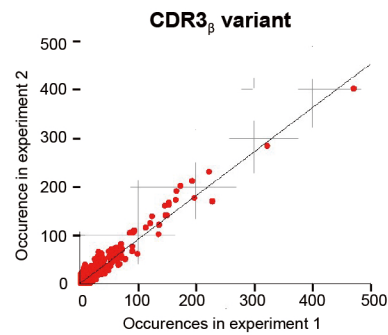

Supplement: Supplementary Figure S18 — Test-retest reliability of ARM-PCR based TCR transcriptome sequencing High test-retest reliability of ARM-PCR based TCR transcriptome sequencing. Two identical aliquots of the same input sample were subjected to ARM-PCR reactions using V/J primers specific for all human TCR chains and then sequenced in one and the same sequencing run. The correlation coefficients between both input samples were 0.99 for distinct V-J combinations (left) and 0.93 for the 10.000 most abundant CDR3 sequences identified (right). [file mmc18.pdf]
