# Supplementary material for: Trilineage Sequencing Reveals Complex TCRβ Transcriptomes in Neutrophils and Monocytes Alongside T Cells
Source: Genomics Proteomics Bioinformatics. 2021 Mar 2;19(6):926–36. doi: 10.1016/j.gpb.2019.02.004 (PMC9402791; doi:10.1016/j.gpb.2019.02.004)

A Individual I

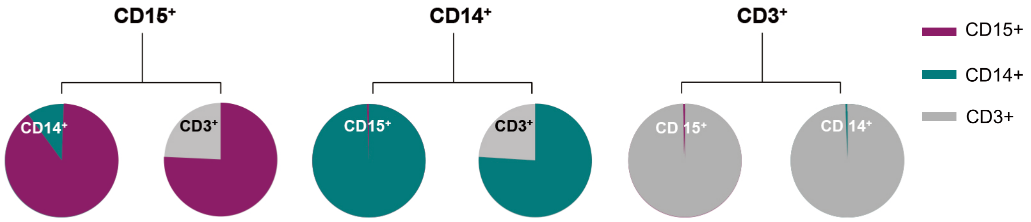

B Individual II

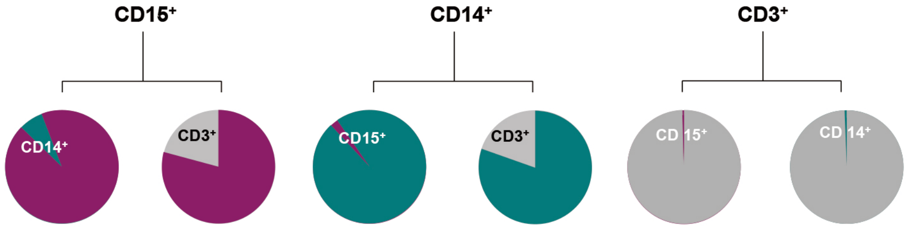

C Individual III

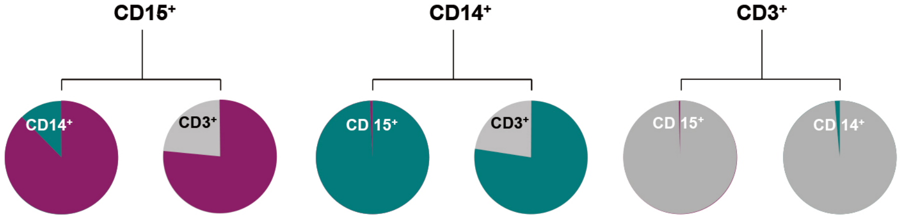

D Individual V

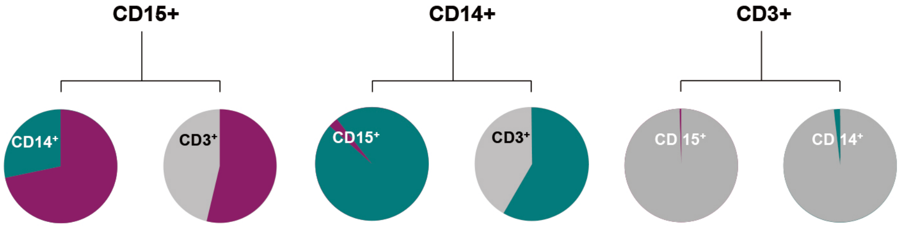

Supplement: Supplementary Figure S10 — Pairwise interlineage comparisons of the TCRβ repertoire transcriptomes identified for individuals I, II, III and V, respectively Pairwise interlineage comparisons of the TCRβ repertoire transcriptomes of human peripheral blood neutrophils (CD15, purple), monocytes (CD14, green) and T cells (CD3, grey), respectively, shown for the healthy individuals I, II, III and V. For each lineage the relative proportions of unique TCRβ CDR3 variants that are also expressed by the respective other lineages are shown in the circle graphs. [file mmc10.pdf]
