# Supplementary material for: Trilineage Sequencing Reveals Complex TCRβ Transcriptomes in Neutrophils and Monocytes Alongside T Cells
Source: Genomics Proteomics Bioinformatics. 2021 Mar 2;19(6):926–36. doi: 10.1016/j.gpb.2019.02.004 (PMC9402791; doi:10.1016/j.gpb.2019.02.004)
Supplement: Supplementary Figure S14 — Quantitative effect of CD3+→CD15+and CD3+→CD3+cell crosscontamination on TCRβ CDR3 repertoire profiles Quantitative effect of CD3+→CD15+ (A and B) and CD3+→CD3+ (C) cross-contamination on TCRβ CDR3 repertoire profiles. CD3+→CD15+ cross-contamination. CD15+ cells from two individuals (donor IV, donor V) were mixed with known numbers of their own CD3+ cells (1%, 3% and 10%) and subjected to ARM-PCR based high-throughput sequencing. The 50 most frequently expressed CDR3 variants expressed by donor IV (A) and V (B) are listed for all samples in descending order. Numbers designate TCRβ CDR3 transcript frequencies. Red lines track the positions of representative CD3+ specific CDR3 sequences in the cell mixes. Note that the transcript frequencies are proportional to the amount of input CD3+ cells and that CD3+ specific CDR3 sequences are already detectable in CD15+ populations at a cross-contamination rate of 1%. C. CD3+→ CD3+ cross-contamination. CD3+ cells from donor III were mixed with CD3+ cells from donor II at the indicated percentages. Blue shaded CDR3 sequences are only expressed by donor II, orange sequences are specifically expressed by donor III. The positions of the most frequently expressed CDR3 sequences in donor II (blue) are are shown in the frequency lists of the mixed cell populations. Arrows demonstrate that the emergence of donor II-specific CDR3 sequences in the mixed cell populations is proportional to the percentage of cross-contamination (for clarity only shown for selected CDR3 variants). The designated color codes are shown in the individual figure panels. [file mmc14.pdf]

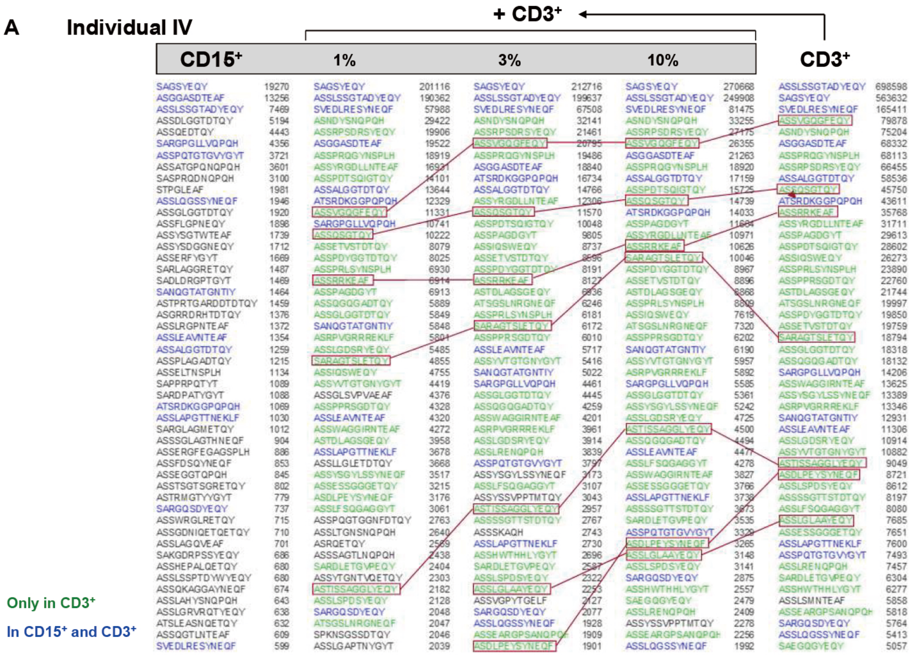
A
Individual IV
+ CD3+
CD15+
1%
3%
10%
CD3+
Only in CD3+
In CD15+ and CD3+

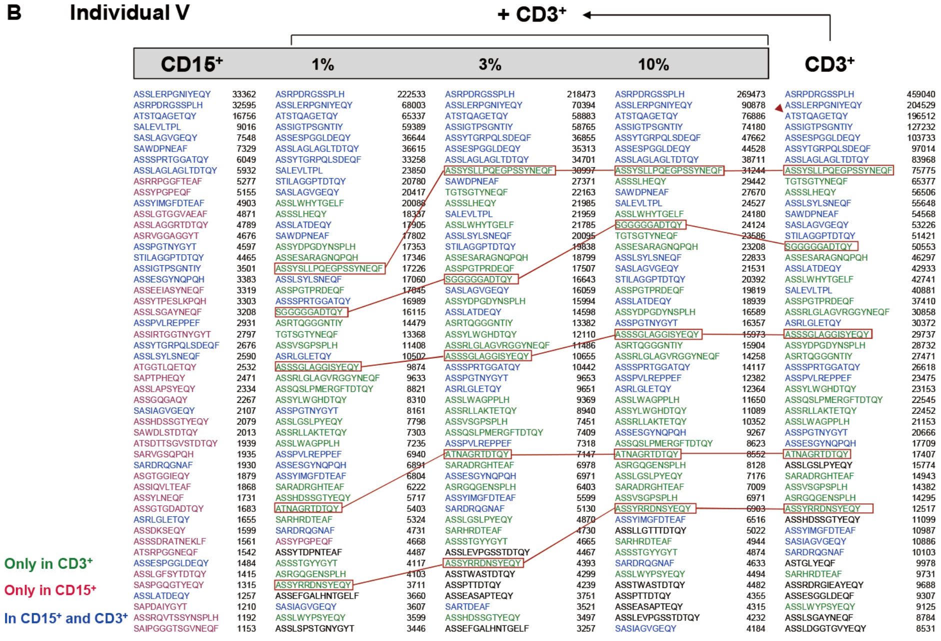
B
Individual V
+ CD3+
CD15+
1%
3%
10%
CD3+
Only in CD3+
Only in CD15+
In CD15+ and CD3+

C

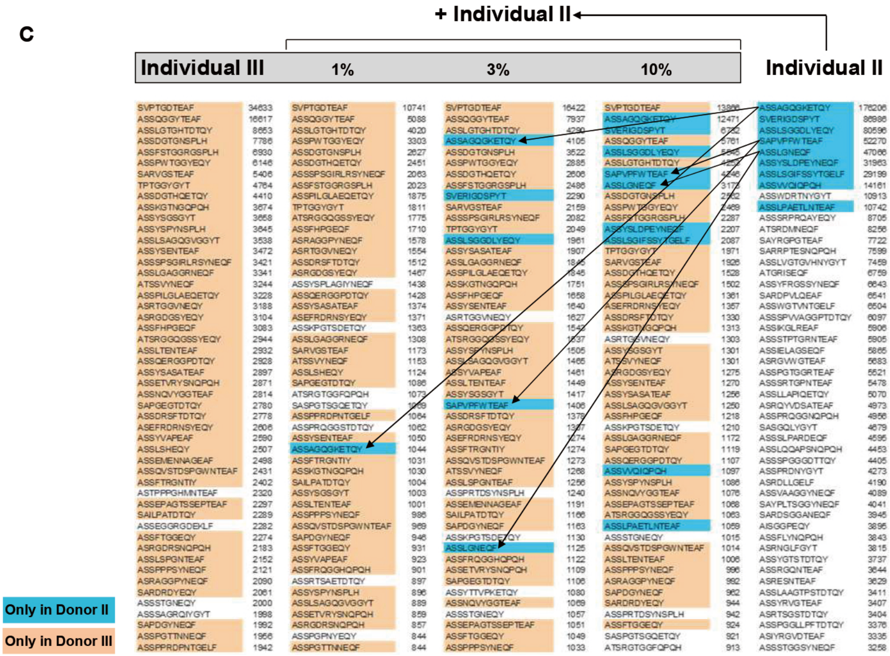

+ Individual II
Individual III
1%
3%
10%
Individual II
Only in Donor II
Only in Donor III
