# Supplementary material for: Trilineage Sequencing Reveals Complex TCRβ Transcriptomes in Neutrophils and Monocytes Alongside T Cells
Source: Genomics Proteomics Bioinformatics. 2021 Mar 2;19(6):926–36. doi: 10.1016/j.gpb.2019.02.004 (PMC9402791; doi:10.1016/j.gpb.2019.02.004)
Supplement: Supplementary Figure S15 — Interlineage discrepancies in the order of transcript frequency between shared CDR3 sequences Synopsis of the 50 most frequently expressed TCRβ CDR3 variants in each lineage shown for donors I–V (A–E). Numbers designate transcript frequencies. CDR3 variants that are shared between distinct leukocyte lineages are highlighted by identical colors. Interlineage discrepancies in the order of transcript frequency between shared CDR3 sequences are highlighted by lines (for clarity shown only for a few selected CDR3 variants). The designated color codes are shown in the individual figure panels. F. Synopsis of 10 shared TCRβ CDR3 variants in each lineage (CD15+, CD14+, M1 macrophages and CD3+) from donor V. TRBC1 and TRBC2 denote the constant chain use of each cell lineage for the respective CDR3 sequence. The respective 10 sequences of donor V are designated with an asterisk (*) in Figure S15E. [file mmc15.pdf]

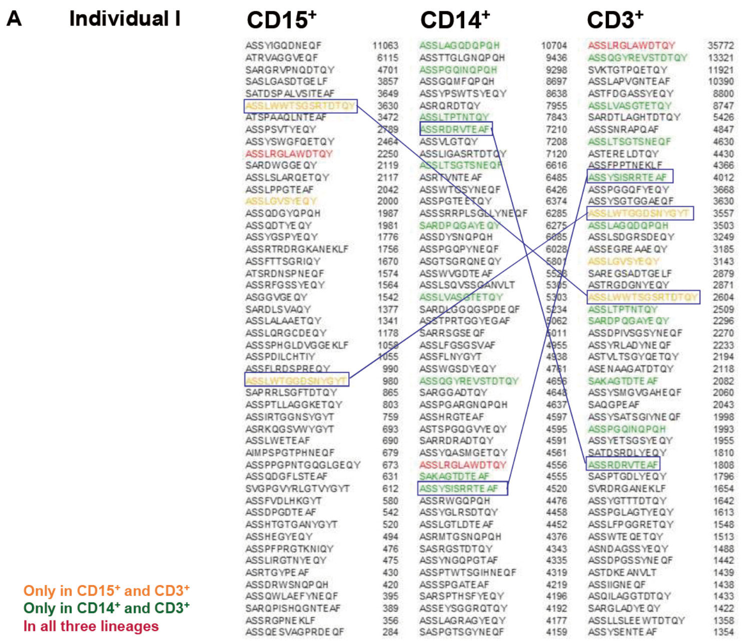
A
Individual I
CD15+
CD14+
CD3+
Only in CD15+ and CD3+
Only in CD14+ and CD3+
In all three lineages

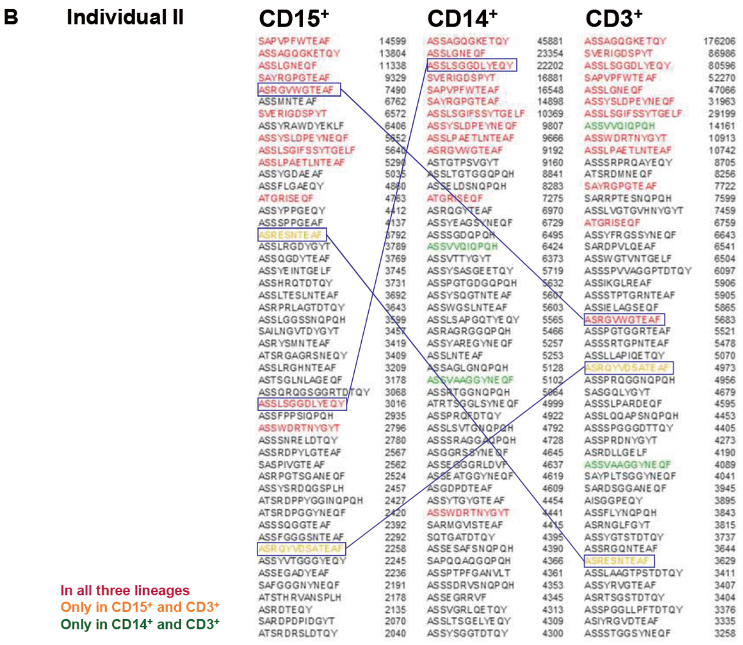
B
Individual II
CD15+
CD14+
CD3+
In all three lineages
Only in CD15+ and CD3+
Only in CD14+ and CD3+

C **Individual III**

CD15⁺		CD14⁺		CD3⁺	
ASNRDGNQPQH	20949	SVPTGDTEAF	86274	SVPTGDTEAF	34633
ASRRSQETQY	18041	ASSLGAGGRNEQF	49852	ASSQGGYTEAF	16617
SVPTGDTEAF	12201	ASSPWTGGYEQY	19226	ASSLGTGHTDTQY	8653
ASSELRTGQGMVSEAF	10899	ASSYSPYNSPLH	13233	ASSDGTGNSPLH	7786
ASSQGNEQF	9772	ASSFSTGGRGSPLH	12209	ASSFSTGGRGSPLH	6930
ASSPIDTVDTQY	9687	ASRTGGVNEQY	11755	ASSPWTGGYEQY	6146
ASQHVGTGRAYEQY	9075	ASSLGTGHTDTQY	8715	SARVGSTEAF	5406
ASSDRRGRYQETQY	8859	ASSPRQGGSTDTQY	8238	TPTGGYGYT	4764
ASSPGLTYNEQF	8397	ASSYSPDSNTEAF	7358	ASSDGTHQETQY	4410
ASRLTGVGYEQY	8232	ASSPPPSYNEQF	7205	ASSKGTNGQPQH	3674
ASSYSRNNEQF	8137	SASPGTSGQETQY	6624	ASSYSGSGYT	3658
ASKGTGPTYEQY	7877	ASSQDMGQNQPQH	6364	ASSYSPYNSPLH	3645
ASSLSLPSGRTEAF	7104	ASSPNSSNQPQH	6315	ASSLSAGQGVGGYT	3538
ASARTGSTDTQY	7091	ASSYSGSGYT	6106	ASSYSENTEAF	3472
SASGGAPDTQY	6559	ASTPPPGHMNTEAF	6005	ASSSPSGIRLRSYNEQF	3421
ASSPRQGGNQPQH	6300	ATSSVYNEQF	5657	ASSLGAGGRNEQF	3341
ASSFNDRSTDTQY	6261	ASSPTGAAAF	5542	ATSSVYNEQF	3244
ASSRTGGINQPQH	6205	SARVGSTEAF	5475	ASSPILGLAEQETQY	3228
SAPLRGGGLYEQY	6148	ASSETVRYSNQPQH	5461	ASRTGGVNEQY	3188
SAPHSGYSYEQY	5988	ASSYSASATEAF	5387	ASRGDGSYEQY	3104
ASSQSDRGKTQY	5865	ASSSTGNEQY	5053	ASSFHPGEQF	3083
ASSSDRGAPYEQY	5360	SARDRDYEQY	4671	ATSRGGQGSSYEQY	2944
ASSHQGDEQY	5294	ASSRIGPTDTQY	4632	ASSLTENTEAF	2932
ATSRIGTSGTDTQY	5272	ASSDSGRDRGTSYEQY	4559	ASSQERGGPDTQY	2928
ASSLWMGANTEAF	5146	ASSELGQPQH	4556	ASSYSASATEAF	2897
ASSELGQPQH	5054	ASSLQGWSGNTEAF	4476	ASSETVRYSNQPQH	2871
ASSPSGAQETQY	4839	ASSPGQGPQPQH	4325	ASSNQVYGGTEAF	2814
ASSLGVLGETQY	4727	ASSFRQGGHQPQH	4310	SAPGEGTDTQY	2780
ASQRQGLYHEQY	4598	ASNVGPYEQY	4101	ASSDRSFTDTQY	2778
ASSENGYGYT	4409	ASSMDRVRAKLF	4028	ASEFRDRNSYEQY	2606
ASSLYPGAYGYT	4399	ASSKPGTSDETQY	4018	ASSYVAPEAF	2590
ATSRDSYEAF	4356	ASSDPGPSGANVLT	3898	ASSLSHEQY	2507
ASSYDQRGQGSYNEQF	4340	ASTSKGSYYNEQF	3868	ASSEMENNAGEAF	2498
SSTGGARAF	4290	ASSKGTNGQPQH	3732	ASSQVSTDSPGWNTEAF	2431
ASSPFPTRQGLDTEAF	4289	ASQPTSGRTDTQY	3723	ASSFTRGNTIY	2402
ASSFPGTGAGYT	4269	ASSLGGSEQY	3681	ASTPPPGHMNTEAF	2320
SARTATTDTQY	4149	ASSQERGGPDTQY	3616	ASSEPAGTSSEPTEAF	2297
ATHSGGRNTEAF	4146	ASSDRDEHNQPQH	3546	SAILPATDTQY	2289
ASSAGTSGADTQY	4105	ASSNVGGNTEAF	3495	ASSEGGRGDEKLF	2282
ASSSSGILNTEAF	4064	ASSDLVNYEQY	3480	ASSFTGGEQY	2274
ASSVGFSDTEAF	3983	ASSYKIETPLH	3444	ASRGDRSNQPQH	2183
ASRLTAQTPNTEAF	3933	ASSSRGLSKKF	3425	ASSLSPGNTEAF	2152
ASTEGPGLAGAAQY	3884	ATSRGGQGSSYEQY	3342	ASSPPPSYNEQF	2121
ASSYSEGANTIY	3813	ASSFRGLTQY	3296	ASRAGGPYNEQF	2090
SAKGAGGAYEQY	3659	ASSFLDRFS	3224	SARDRDYEQY	2061
ASSGSEPPWSYEQY	3585	ASSKGSGNSPLH	3187	ASSSTGNEQY	2000
ASSYSGGAGNTIY	3559	ASSPSGAQETQY	3177	ASSSAGRQIYGYT	1998
SAPPTGTSNYEQY	3464	ASTSGQGDTGELF	3139	SAPDGYNEQF	1992
ATSALGGETQY	3382	ASSLRTSGGANEQF	3126	ASSPGTTNNEQF	1956
ASSEYNQPQH	3264	ASSEQGAPGNTIY	3107	ASSPPRDPNTGELF	1942

In all three lineages
Only in CD15⁺ and CD14⁺
Only in CD14⁺ and CD3⁺

D **Individual IV**

CD15⁺		CD14⁺		CD3⁺	
SAGSYEQY	19270	SAGSYEQY	403070	ASSLSSGTADYEQY	698598
ASGGASDTEAF	13256	ASSLSSGTADYEQY	288607	SAGSYEQY	563632
ASSLSSGTADYEQY	7469	ASGGASDTEAF	83937	SVEDLRESYNEQF	165411
ASSDLGGTDTQY	5194	ASNDYSNQPQH	39117	ASSVGQGFEQY	79878
ASSQEDTQY	4443	SVEDLRESYNEQF	31433	ASNDYSNQPQH	75204
SARGPGLLVQPQH	4356	SARGPGLLVQPQH	24911	ASGGASDTEAF	68332
ASSPQTGTGVYGYT	3721	ASSPDTSQIGTQY	19795	ASSPRQGYNSPLH	68113
ASSATGPQNQPQH	3601	ASSVGQGFEQY	17536	ASSRPSDRSYEQY	66455
SASPRQDNQPQH	3100	ASSALGGTDTQY	15360	ASSALGGTDTQY	58536
STPGLEAF	1981	ASSRPSDRSYEQY	14829	ASSQSGTQY	45750
ASSLQGSSYNEQF	1946	ASSLRENQPQH	14578	ATSRDKGGPQPQH	43611
ASSGLGGTDTQY	1920	ASSPDYGGTDTQY	13302	ASSRRKEAF	35768
ASSFLGPNEQY	1898	ASTDLAGSGEQY	12882	ASSYRGDLLNTEAF	31711
ASSYSGTWTEAF	1739	ASSRRKEAF	12374	ASSPAGDGYT	29613
ASSYSDGGNEQY	1712	ASSPRQGYNSPLH	11529	ASSPDTSQIGTQY	28602
ASSERFYGYT	1669	ASSPQTGTGVYGYT	10456	ASSIQSWEQY	26273
SARLAGGRETQY	1487	ASSLQGSSYNEQF	10101	ASSPRLSYNSPLH	23890
SADLDRGPTGYT	1469	ASSQSGTQY	8981	ASSPPRSGDTQY	22760
SANQGTATGNTIY	1464	SARAGTSLETQY	8946	ASTDLAGSGEQY	21744
ASTPRTGARDDTDTQY	1459	ATSRDKGGPQPQH	8944	ATSGSLNRGNEQF	19997
ASGRRDRHTDTQY	1376	ASSGLGGTDTQY	8433	ASSPDYGGTDTQY	19850
ASSLRGPNTEAF	1372	ASSSAGTLNQPQH	8419	ASSETVSTDTQY	19759
ASSLEAVNTEAF	1354	ASSQGQGADTQY	7847	SARAGTSLETQY	18794
ASSALGGTDTQY	1259	SANQGTATGNTIY	7538	ASSGLGGTDTQY	18318
ASSPLAGADTQY	1215	ASSFGLDTQY	7411	ASSQGQGADTQY	18132
ASSELTNSPLH	1134	ASSPTSGAYNEQF	7156	SARGPGLLVQPQH	14206
SAPPRPQTYT	1089	ASSLEAVNTEAF	6634	ASSWAGGIRNTEAF	13625
SARDPATYGYT	1088	ASSWGTDTQY	6566	ASSYSGYLSSYNEQF	13389
ATSRDKGGPQPQH	1069	ASSPDLVTQH	6173	ASRPVGRRREKLF	13346
ASSLAPGTTNEKLF	1030	SAEGQGYEQY	6171	SANQGTATGNTIY	12931
SARGLAGMETQY	1012	ASSYRGDLLNTEAF	5963	ASSLEAVNTEAF	11306
ASSSGLAGTHNEQF	904	ASSLTSPVDTQY	5911	ASSLGDSRYEQY	10914
ASSERGFEGAGSPLH	886	ASGDEQY	5901	ASSYVTGTGNYGYT	10882
ASSFDSQYNEQF	853	ASSETGGSYNEQF	5823	ASTISSAGGLYEQY	9049
ASSEGGTQPQH	845	ASSETVSTDTQY	5805	ASDLPEYSYNEQF	8721
ASSTSGTSGRETQY	802	ASSELTNSPLH	5615	ASSLSPDSYEQY	8612
ASTRMGTYYGYT	779	ASLWSGARGEQY	5424	ASSSSGTTSTDTQY	8197
SARGQSDYEQY	737	ATSTKDRRTDTQY	5399	ASSLFSQGAGGYT	8080
ASSWRGLRETQY	715	ASSPPRSGDTQY	5332	ASSLGLAAYEQY	7685
ASSGDNIQETQETQY	710	ASSFGTDQNTEAF	5293	ASSESSGGGETQY	7651
ASSLAGQVEAF	701	ATSLADTQY	5271	ASSLAPGTTNEKLF	7600
SAKGDRPSSYEQY	686	ASSESSGGGETQY	5267	ASSPQTGTGVYGYT	7493
ASSHEPALQETQY	680	SAPNPSGRLDTQY	5217	ASSLRENQPQH	7457
ASSLSSPTDYWYEQY	680	ASRQGNQPQH	5212	SARDLETGVPEQY	6304
ASSQKAGGAYNEQF	674	ASSDISGGPDTQY	5200	ASSHWTHHLYGYT	6277
ASSLAHYSNQPQH	643	ASRWTGSTDTQY	5191	ASSLSMNTEAF	5858
ASSLGRVRQTYEQY	638	ASSRDRFNQPQH	5189	ASSEARGPSANQPQH	5818
ATSLEASNQETQY	632	ASSEGRGTDTQY	5135	SARGQSDYEQY	5764
ASSQGTLNTEAF	609	ASSETGTGGYEQY	5112	ASSLQGSSYNEQF	5413
SVEDLRESYNEQF	599	SVGDYPYNEQF	5004	SAEGQGYEQY	5057

In all three lineages
Only in CD15⁺ and CD14⁺
Only in CD15⁺ and CD3⁺
Only in CD14⁺ and CD3⁺

E **Individual V**

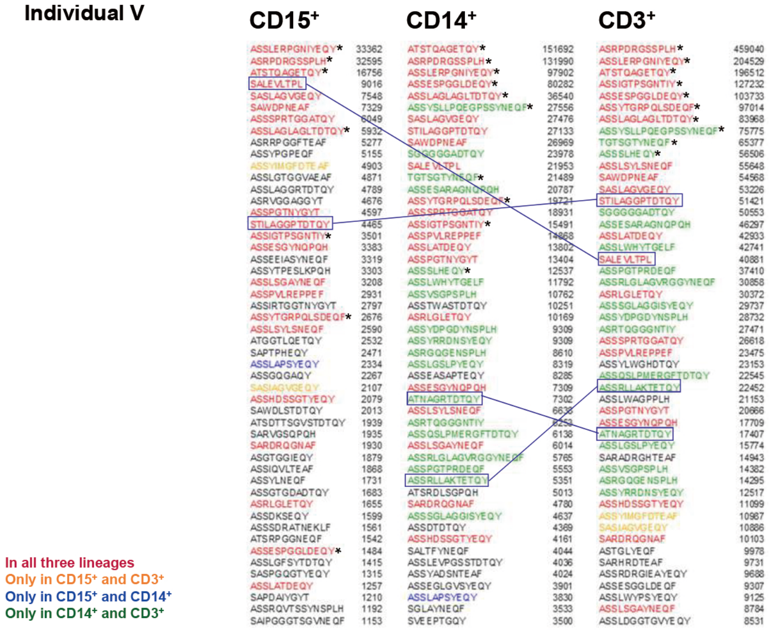

F **Individual V**

	CD15+	CD14+	M1 Mac	CD3+
ASRPDRGSSPLH	TRBC2	TRBC2	TRBC2	TRBC2
ASSLERPGNIYEQY	TRBC2	TRBC1	TRBC2	TRBC2
ATSTQAGETQY	TRBC2	TRBC1	TRBC1	TRBC2
ASSIGTPSGNTIY	TRBC2	TRBC2	TRBC2	TRBC2
ASSESPGGLDEQY	TRBC1	TRBC2	TRBC2	TRBC2
ASSYTGRPQLSDEQF	TRBC2	TRBC1	TRBC2	TRBC1
ASSLAGLAGLTDTQY	TRBC2	TRBC2	TRBC1	TRBC2
ASSYSLLPQEGPSSYNEQF	TRBC2	TRBC2	TRBC2	TRBC2
TGTSGTYNEQF	TRBC2	TRBC2	TRBC2	TRBC2
ASSSLHEQY	TRBC2	TRBC1	TRBC1	TRBC1
