# Supplementary material for: Trilineage Sequencing Reveals Complex TCRβ Transcriptomes in Neutrophils and Monocytes Alongside T Cells
Source: Genomics Proteomics Bioinformatics. 2021 Mar 2;19(6):926–36. doi: 10.1016/j.gpb.2019.02.004 (PMC9402791; doi:10.1016/j.gpb.2019.02.004)
Supplement: Supplementary Table S1 — CDR3 sequence reads for human circulating neutrophils, monocytes, T cells and monocyte-derived M1 macrophages [file mmc19.rtf]

Table S1  CDR3 sequence reads for human circulating neutrophils, monocytes, T cells and monocyte-derived M1 macrophages

 	 	 	 	conventional data analysis	SMART-filtered3	
donor	cell type	effective read1	total CDR3	unique CDR32	unique CDR32	
I	CD15+	92,300	90,827	441	207	
II	CD15+	445,239	444,296	4289	2488	
III	CD15+	579,164	566,220	1532	773	
IV	CD15+	133,132	130,462	691	312	
V	CD15+	286,992	285,005	914	430	
						
I	CD14+	1,666,948	1,641,685	7483	4443	
II	CD14+	2,785,325	2,758,123	10,030	7634	
III	CD14+	4,296,329	4,239,650	29,444*	22,397*	
IV	CD14+	3,182,266	3,159,272	9232	6276	
V	CD14+	1,806,180	1,783,243	5895	4107	
						
I	CD3+	2,491,260	2.458.873	565,116	328,478	
II	CD3+	5,802,976	5.749.922	364,274	268,271	
III	CD3+	5,184,813	5.126.537	487,313	348,533	
IV	CD3+	6,211,806	6.159.195	417,338	288,580	
V	CD3+	5,662,591	5.613.301	210,033	146,890	
						
I	M1 MФ	48,028	47,396	660	201	
IV	M1 MФ	79	79	122	14	
V	M1 MФ	1,062,505	1,050,132	6409	1438	
						
∑		41,737,933	41,304,218	2,121,216	1,431,472	

1 An effective read is a read that can be mapped to both V and J germline segments
2 A unique CDR3 sequence is defined as a nonredundant fragment of amino acids which is derived from a stop-codon-free reading frame containing both translated conserved V and J motifs 					
3 The comprehensive SMART filtering strategy eliminates erroneous CDR3 sequences at the following five quality control levels: Sequencing errors, Mosaic recognition, PCR Amplification errors, Reference sequence information and frequency Threshold 
CD15+, neutrophils, CD14+ monocytes, CD3+ T cells, M1 MФ, IFNγ polarized macrophages			
* ~4% T cell content in CD14 sample					
					
