# Supplementary material for: Trilineage Sequencing Reveals Complex TCRβ Transcriptomes in Neutrophils and Monocytes Alongside T Cells
Source: Genomics Proteomics Bioinformatics. 2021 Mar 2;19(6):926–36. doi: 10.1016/j.gpb.2019.02.004 (PMC9402791; doi:10.1016/j.gpb.2019.02.004)
Supplement: Supplementary Table S2 — Unique TCRβ CDR3 variants shared between neutrophils, monocytes and T cells in each individual [file mmc20.rtf]

Table S2  Unique TCRâ CDR3 variants shared between neutrophils, monocytes and T cells in each individual
	
CD15+ neutrophils	I	II	III	IV	V	
unique CDR3 in CD15	207	2488	773	312	430	
shared with CD14	23	150	95	67	121	
CD15 minus (CD15 shared with CD14)	184	2338	678	245	309	
shared with CD3	51	533	186	130	199	
CD15 minus (CD15 shared with CD3)	156	1.955	587	182	231	
exclusive CDR3	133	1.805	492	115	110	
						
						
CD14+ monocytes	I	II	III	IV	V	
unique CDR3 in CD14	4443	7634	22,397*	6276	4107	
shared with CD15	23	150	95	67	121	
CD14 minus (CD14 shared with CD15)	4420	7484	22,302*	6209	3986	
shared with CD3	1073	1528	5126	1710	1662	
CD14 minus (CD14 shared with CD3)	3370	6106	17,271*	4566	2445	
exclusive CDR3	3347	.956	17,176*	4499	2324	
						
						
CD3+ T cells	I	II	III	IV	V	
unique CDR3 in CD3	328,478	268,k271	348,533	288,580	146,890	
shared with CD14	1073	1528	5126	1710	1662	
CD3 minus (CD3 shared with CD14)	327,405	266,743	343,407	286,870	145,228	
shared with CD15	51	533	186	130	199	
CD3 minus (CD3 shared with CD15)	328,054	267,847	348,109	288,156	146,466	
exclusive CDR3	327,354	266,210	343,221	286,740	145,029	

* ~4% T cell content in CD14 sample
