# Supplementary material for: Trilineage Sequencing Reveals Complex TCRβ Transcriptomes in Neutrophils and Monocytes Alongside T Cells
Source: Genomics Proteomics Bioinformatics. 2021 Mar 2;19(6):926–36. doi: 10.1016/j.gpb.2019.02.004 (PMC9402791; doi:10.1016/j.gpb.2019.02.004)
Supplement: Supplementary Table S3 — Shared CDR3 and interlineage cross-contamination [file mmc21.rtf]

Table S3  Shared CDR3 and interlineage cross-contamination
	
CD15+ neutrophils	I	II	III	IV	V	mean	
							
CDR3 shared with CD14	11.1	6.0	12.3	21.5	28.1	15.8	
CDR3 shared with CD3	24.6	21.4	24.1	41.7	46.3	31.6	
							
CD14+ content 	0.0	0.0	0.0	0.0	0.0	0.0	
CD3+ content 	0.1	0.0	0.0	0.0	0.1	0.0	
							
							
CD14+ monocytes	I	II	III	IV	V	mean	
							
CDR3 shared with CD15	0.5	2.0	0.4	1.1	2.9	1.4	
CDR3 shared with CD3	24.2	20.0	22.9	27.2	40.5	27.0	
							
CD15+ content 	9.8	0.3	29.0	1.6	0.1	8.2	
CD3+ content 	0.9	0.5	4.1*	0.9	0.9	0.8	
							
							
CD3+ T cells	I	II	III	IV	V	mean	
							
CDR3 shared with CD15	0.3	0.6	1.5	0.6	1.1	0.8	
CDR3 shared with CD14	0.0	0.2	0.1	0.0	0.1	0.1	
							
CD15+ content 	3.8	11.2	2.0	3.7	0.0	4.1	
CD14+ content 	0.2	0.2	0.0	0.2	0.0	0.1	

Note: Shown are the percentages of CDR3 sequences that are shared between two lineages in each individual and the cross-contamination rates (%) for each lineage as assessed by flow cytometry.
* significant T cell contamination
