# Supplementary material for: Trilineage Sequencing Reveals Complex TCRβ Transcriptomes in Neutrophils and Monocytes Alongside T Cells
Source: Genomics Proteomics Bioinformatics. 2021 Mar 2;19(6):926–36. doi: 10.1016/j.gpb.2019.02.004 (PMC9402791; doi:10.1016/j.gpb.2019.02.004)
Supplement: Supplementary Table S4 — Sharing of TCRβ CDR3 sequences expressed by all three leukocyte lineages (“trilineage expression”) [file mmc22.rtf]

Table S4  Sharing of TCRâ CDR3 sequences expressed by all three leukocyte lineages ("trilineage expression") in each individual

A
Individual	I	II	III	IV	V	
Total number of CDR3 sequences expressed by all three lineages	333,128	278,393	371,703	295,168	151,427	
Total number of shared trilineage CDR3 sequences	21	130	79	62	116	
Shared trilineage CDR3 sequences (% of total)	0.0063	0.0467	0.0213	0.0210	0.0766	

B
Pairwise comparison of trilineage shared CDR3 (% of shared trilineage CDR3 sequences)
					
 	II	III	IV	V	
I	0.0033	0.0022	0.0022	0.0044	
II	/	0.0048	0.0035	0.0	
III	/	/	0.0026	0.0046	
IV	/	/	/	0.0045	
V	/	/	/	/	
