# Supplementary material for: Trilineage Sequencing Reveals Complex TCRβ Transcriptomes in Neutrophils and Monocytes Alongside T Cells
Source: Genomics Proteomics Bioinformatics. 2021 Mar 2;19(6):926–36. doi: 10.1016/j.gpb.2019.02.004 (PMC9402791; doi:10.1016/j.gpb.2019.02.004)
Supplement: Supplementary Table S5 — TCRβ CDR3 sequences exclusive to one lineage and simultaneously shared between all donors (“lineage-specific AND public”) [file mmc23.rtf]

Table S5  TCRâ CDR3 sequences exclusive to one lineage and simultaneously shared between all donors ("lineage-specific AND public")

CD15+ neutrophils	CD14+ monocytes	CD3+ T cells (n=3447)	CD3+ T cells	
(n= 0)	ASGGASDTEAF 	AISESYEQY	ASRGNEKLF	
	SAPVPFWTEAF 	ASGGNEQF	ASRGNTEAF	
	SVEDLRESYNEQF 	ASGGNTEAF	ASRGQETQY	
	SVERIGDSPYT 	ASGGSYEQY	ASRGQGDTEAF	
	(n= 4)	ASGGTDTQY	ASRGQGNQPQH	
		ASGGYTDTQY	ASRGQGNTEAF	
		ASGLGNQPQH	ASRGQGSYEQY	
		ASGLNTEAF	ASRGQGYGYT	
		ASGNQPQH	ASRGQPNTEAF	
		ASGPYEQY	ASRGSNQPQH	
		ASGPYNEQF	ASRGTDTQY	
		ASGQETQY	ASRGTGSNQPQH	
		ASGRNTEAF	ASRGYGYT	
		ASGRTDTQY	ASRGYTEAF	
		ASGSPLH	ASRIQETQY	
		ASGSSYEQY	ASRKETQY	
		ASGSYEQY	ASRKNTEAF	
		ASGSYNEQF	ASRLADTQY	
		ASKGNTEAF	ASRLAGGPYNEQF	
		ASKGSTDTQY	ASRLAGGTDTQY	
		ASKGSYEQY	ASRLNTEAF	
		ASKGTDTQY	ASRLNTGELF	
		ASMGGTEAF	ASRLQGNTEAF	
		ASRAGGSYEQY	ASRPDSSYEQY	
		ASRAGNTEAF	ASRPSGANVLT	
		ASRAQETQY	ASRQGDYGYT	
		ASRDRAYEQY	ASRQGNTEAF	
		ASRDRGNQPQH	ASRQGPYEQY	
		ASRDRGNTEAF	ASRQGSNQPQH	
		ASRDRGTDTQY	ASRQNTEAF	
		ASRDRGYEQY	ASRQQGNTEAF	
		ASRDRGYGYT	ASRRDRTDTQY	
		ASRDRNTEAF	ASRRDSSYEQY	
		ASRDRSYEQY	ASRRDTEAF	
		ASRDSNQPQH	ASRRETQY	
		ASRDSSGNTIY	ASRRGLNTEAF	
		ASRDSSYEQY	ASRRGNTEAF	
CD3+ T cells	CD3+ T cells	CD3+ T cells	CD3+ T cells	
ASRDSYNEQF	ASRRGQETQY	ASSESPGGLDEQY	ASSFGGSNQPQH	
ASRDTGELF	ASRRGSYEQY	ASSESPYEQY	ASSFGGSPLH	
ASREGNTEAF	ASRRGTDTQY	ASSESSYEQY	ASSFGGSSYEQY	
ASRENTEAF	ASRRNTEAF	ASSESTDTQY	ASSFGGSTDTQY	
ASRENTGELF	ASRRSTDTQY	ASSESYEQY	ASSFGGSYEQY	
ASREVNTEAF	ASRRTENTEAF	ASSETGNQPQH	ASSFGGSYNEQF	
ASRGDSSYEQY	ASRRTVNTEAF	ASSETGSNQPQH	ASSFGGTDTQY	
ASRGETQY	ASRSGGSSYEQY	ASSETYEQY	ASSFGGTEAF	
ASRGGGSNQPQH	ASRSGTYEQY	ASSEYYEQY	ASSFGGYEQY	
ASRGGGTDTQY	ASRSNTEAF	ASSFAETQY	ASSFGLSSYNEQF	
ASRGGNQPQH	ASRSQGNTEAF	ASSFAGGPTDTQY	ASSFGLYNEQF	
ASRGGNTEAF	ASRSSGNTIY	ASSFAGNQPQH	ASSFGMNTEAF	
ASRGGSYEQY	ASRTANTEAF	ASSFAGNTEAF	ASSFGNEQF	
ASRGGTDTQY	ASRTGAYEQY	ASSFAGSYEQY	ASSFGNQPQH	
ASRGGTEAF	ASRTGGNTEAF	ASSFAGTEAF	ASSFGNSPLH	
ASRGLTDTQY	ASRTGGTDTQY	ASSFATDTQY	ASSFGNSYEQY	
ASRTGNTEAF	ASSDPYEQY	ASSFAYEQY	ASSFGNTEAF	
ASRTGSYEQY	ASSDRASYEQY	ASSFDETQY	ASSFGNTIY	
ASRTGYTEAF	ASSDRDTDTQY	ASSFDGNTEAF	ASSFGPDTQY	
ASRTSGSTDTQY	ASSDRGETQY	ASSFDGSNQPQH	ASSFGPNEQF	
ASRTSGSYEQY	ASSDRGGNQPQH	ASSFDRGSYEQY	ASSFGPNTEAF	
ASRTSTDTQY	ASSDRGGTDTQY	ASSFDRNTEAF	ASSFGPQETQY	
ASRTYNEQF	ASSDRGNQPQH	ASSFDRSNQPQH	ASSFGPSYEQY	
ASRVSSGNTIY	ASSDRGQPQH	ASSFDSYEQY	ASSFGQANTEAF	
ASRVTDTQY	ASSDRGSNQPQH	ASSFEETQY	ASSFGQAYEQY	
ASRWGTEAF	ASSDRLNTEAF	ASSFEGNQPQH	ASSFGQETQY	
ASS	ASSDRNQPQH	ASSFEGTEAF	ASSFGQGAYEQY	
ASSADRNTEAF	ASSDRNTGELF	ASSFEVNTEAF	ASSFGQGNTEAF	
ASSADSNQPQH	ASSDRNYGYT	ASSFFNQPQH	ASSFGQGYEQY	
ASSADSSYEQY	ASSDRSSYNEQF	ASSFGADTQY	ASSFGQLNTEAF	
ASSADSYGYT	ASSDRYEQY	ASSFGATDTQY	ASSFGQNTEAF	
ASSAETQY	ASSDSAGGTDTQY	ASSFGDGNTEAF	ASSFGQNYGYT	
ASSAGANTEAF	ASSDSNQPQH	ASSFGDQETQY	ASSFGQTYEQY	
ASSAGGNTEAF	ASSDSNTGELF	ASSFGDQPQH	ASSFGRNTEAF	
ASSAGGTDTQY	ASSDSSGGTDTQY	ASSFGDSSYEQY	ASSFGRSYEQY	
ASSAGGYGYT	ASSDSSGSTDTQY	ASSFGDTEAF	ASSFGSEQY	
ASSAGNQPQH	ASSDSSTDTQY	ASSFGDTQY	ASSFGSNQPQH	
ASSAGNTEAF	ASSDSYEQY	ASSFGEKLF	ASSFGSNYGYT	
ASSAGSNQPQH	ASSDSYNEQF	ASSFGETQY	ASSFGSSYEQY	
ASSAGSSYEQY	ASSDTDTQY	ASSFGGADTQY	ASSFGSTDTQY	
ASSAGSYNEQF	ASSDYNEQF	ASSFGGAYEQY	ASSFGSYEQY	
ASSAGTASYEQY	ASSEAENTEAF	ASSFGGAYNEQF	ASSFGTDTQY	
ASSAGTDSNQPQH	ASSEAGGTDTQY	ASSFGGEQY	ASSFGTEAF	
ASSAGTEAF	ASSEDSSGANVLT	ASSFGGGNQPQH	ASSFGTGAYEQY	
ASSAGTGAYEQY	ASSEGDTQY	ASSFGGGSTDTQY	ASSFGTGELF	
ASSAGTGPYEQY	ASSEGETQY	ASSFGGMNTEAF	ASSFGTGNTEAF	
ASSAGTGSYEQY	ASSEGGEQF	ASSFGGNEQF	ASSFGTTYEQY	
ASSAGYEQY	ASSEGGETQY	ASSFGGNQPQH	ASSFGTVNTEAF	
ASSAHNEQF	ASSEGGNTEAF	ASSFGGNSNQPQH	ASSFGTYEQY	
ASSALGGTDTQY	ASSEGGTDTQY	ASSFGGNTEAF	ASSFGVNTEAF	
ASSANTEAF	ASSEGGYEQY	ASSFGGNYGYT	ASSFGYEQY	
ASSAPGTDTQY	ASSEGLNQPQH	ASSFGGPYEQY	ASSFGYGYT	
ASSAQETQY	ASSEGLNTEAF	ASSFGGQETQY	ASSFITDTQY	
ASSAQGSYEQY	ASSEGLYNEQF	ASSFGGQPQH	ASSFKDTQY	
ASSAQGYEQY	ASSEGNQPQH	ASSFGGRETQY	ASSFKNTEAF	
ASSARGNTEAF	ASSEGPNTEAF	ASSFGGSGNTIY	ASSFLDTEAF	
ASSARGTDTQY	ASSEGQGYEQY	ASSGGNTEAF	ASSIDRGGNQPQH	
ASSASGGTDTQY	ASSEGSSGNTIY	ASSGGSSYNEQF	ASSIGGNQPQH	
ASSASGSSYNEQF	ASSEGSYEQY	ASSGGSTDTQY	ASSIGGNTEAF	
ASSASTDTQY	ASSEGTDTQY	ASSGGSYEQY	ASSIGLNTEAF	
ASSASYEQY	ASSEGYEQY	ASSGGYEQY	ASSIGNTEAF	
ASSATDTQY	ASSEGYSNQPQH	ASSGGYGYT	ASSIGQNTEAF	
ASSATGGNQPQH	ASSELAGGPDTQY	ASSGLAGYNEQF	ASSIGSNQPQH	
ASSATGNTEAF	ASSELAGGQETQY	ASSGLDTQY	ASSIGTDTQY	
ASSATYEQY	ASSELAGGTDTQY	ASSGLNTEAF	ASSIGTGSNQPQH	
ASSDGLNTEAF	ASSELAGGYNEQF	ASSGLYEQY	ASSIGYEQY	
ASSDGMNTEAF	ASSELNEQF	ASSGNEQF	ASSIMNTEAF	
ASSDGTSGTDTQY	ASSEQETQY	ASSGNSNQPQH	ASSINQPQH	
ASSDGYNEQF	ASSEQGAYEQY	ASSGNTEAF	ASSINTEAF	
ASSDLYEQY	ASSEQGNTEAF	ASSGQETQY	ASSIRNTEAF	
ASSDNSPLH	ASSEQGYEQY	ASSGQGAEAF	ASSISANTEAF	
ASSDPNQPQH	ASSERGNTEAF	ASSGQGAYEQY	ASSISGNTEAF	
ASSFLETQY	ASSFSGANVLT	ASSGQGGQPQH	ASSISTDTQY	
ASSFLGETQY	ASSFSGDQPQH	ASSGQGNQPQH	ASSISYEQY	
ASSFLGNEQF	ASSFSGEETQY	ASSGQGNSPLH	ASSITDTQY	
ASSFLNTEAF	ASSFSGELF	ASSGQGNTGELF	ASSITGSNQPQH	
ASSFNQPQH	ASSFSGGNQPQH	ASSGQGTYEQY	ASSIVGTDTQY	
ASSFPGEETQY	ASSFSGGPYNEQF	ASSGQGYEQY	ASSIYNEQF	
ASSFPGNEQF	ASSFSGGQETQY	ASSGQGYGYT	ASSKDRAYEQY	
ASSFPGNTEAF	ASSFSGGSYEQY	ASSGQMNTEAF	ASSKETQY	
ASSFPGSSYNEQF	ASSFSGGTDTQY	ASSGQNYGYT	ASSKTGGTEAF	
ASSFPGTDTQY	ASSFSGGYNEQF	ASSGQVNTEAF	ASSLAADTQY	
ASSFPGTEAF	ASSFSGNQPQH	ASSGSDTQY	ASSLAAEAF	
ASSFPNTEAF	ASSFSGNTEAF	ASSGSGNTIY	ASSLAAGMNTEAF	
ASSFPSTDTQY	ASSFSGNTIY	ASSGSNQPQH	ASSLAANEQF	
ASSFPYNEQF	ASSFSGQETQY	ASSGSSTDTQY	ASSLAANQPQH	
ASSFQENTEAF	ASSFSGSTDTQY	ASSGSSYEQY	ASSLAANTEAF	
ASSFQETQY	ASSFSGSYEQY	ASSGTANTEAF	ASSLAASTDTQY	
ASSFQGATEAF	ASSFSGSYNEQF	ASSGTDTQY	ASSLAASYEQY	
ASSFQGGTEAF	ASSFSGYEQY	ASSGTEETQY	ASSLADNEQF	
ASSFQGNTEAF	ASSFSLNTEAF	ASSGTGGNQPQH	ASSLADSYEQY	
ASSFQGSYEQY	ASSFSMNTEAF	ASSGTGGYEQY	ASSLADTQY	
ASSFQGYNEQF	ASSFSNQPQH	ASSGTGNQPQH	ASSLAENQPQH	
ASSFQMNTEAF	ASSFSQETQY	ASSGTGPYEQY	ASSLAENTEAF	
ASSFQNEQF	ASSFSRDTEAF	ASSGTSGSTDTQY	ASSLAETQY	
ASSFQSSYEQY	ASSFSSGNTIY	ASSGTSGTDTQY	ASSLAFQETQY	
ASSFRETQY	ASSFSSNQPQH	ASSGTSYEQY	ASSLAGADTQY	
ASSFRGAEAF	ASSFSSQETQY	ASSGTTYEQY	ASSLAGAGTDTQY	
ASSFRGDTQY	ASSFSSSYEQY	ASSGTVNTEAF	ASSLAGANVLT	
ASSFRGETQY	ASSFSSYEQY	ASSGTYNEQF	ASSLAGAPYEQY	
ASSFRGGNQPQH	ASSFSTEAF	ASSGYTDTQY	ASSLAGAQETQY	
ASSFRGGNTEAF	ASSFSYEQY	ASSHDSYEQY	ASSLAGASTDTQY	
ASSFRGGQETQY	ASSFSYNEQF	ASSHGQGYEQY	ASSLAGAYEQY	
ASSFRGGQPQH	ASSFTANTEAF	ASSHGYEQY	ASSLAGAYNEQF	
ASSFRGGTEAF	ASSFTDTQY	ASSHLYEQY	ASSLAGDEQF	
ASSFRGNQPQH	ASSFTGGSYEQY	ASSHRDTEAF	ASSLAGDNEQF	
ASSFRGQETQY	ASSFTGGYGYT	ASSHRGSYEQY	ASSLAGDQPQH	
ASSFRGSGNTIY	ASSFTGNEQF	ASSHRGYEQY	ASSLAGDTDTQY	
ASSFRGSNTEAF	ASSFTGNTEAF	ASSHSYEQY	ASSLAGDTQY	
ASSFRGSYEQY	ASSFTGSSYEQY	ASSHSYNEQF	ASSLAGDYGYT	
ASSFRGSYNEQF	ASSFTGSTDTQY	ASSHTDTQY	ASSLAGEAF	
ASSFRGTEAF	ASSFTTEAF	ASSHYQETQY	ASSLAGEETQY	
ASSFRGYEQY	ASSFTYEQY	ASSLAQSSYEQY	ASSLDRAYEQY	
ASSFRGYNEQF	ASSFTYNEQF	ASSLARETQY	ASSLDRDQPQH	
ASSFRLNTEAF	ASSFVNEQF	ASSLARGNEQF	ASSLDRETQY	
ASSFRNEQF	ASSFYTDTQY	ASSLARNTEAF	ASSLDRGADTQY	
ASSFRNTEAF	ASSGADTQY	ASSLARTEAF	ASSLDRGGQPQH	
ASSFRNTGELF	ASSGAYEQY	ASSLASDTQY	ASSLDRGGTDTQY	
ASSFRNYGYT	ASSGDSNQPQH	ASSLASGYEQY	ASSLDRGLTEAF	
ASSFRQNTEAF	ASSGDSSYEQY	ASSLASNQPQH	ASSLDRGLYEQY	
ASSFRSSGNTIY	ASSGDSYEQY	ASSLASNTEAF	ASSLDRGNEQF	
ASSFRVNTEAF	ASSGGDTQY	ASSLASSGNTIY	ASSLDRGNQPQH	
ASSFSADTQY	ASSGGGETQY	ASSLASSYEQY	ASSLDRGNTEAF	
ASSFSANYGYT	ASSGGGNQPQH	ASSLASSYNEQF	ASSLDRGPYEQY	
ASSFSDTQY	ASSGGGTDTQY	ASSLASTDTQY	ASSLDRGQPQH	
ASSFSETQY	ASSGGGTEAF	ASSLASYEQY	ASSLDRGRTEAF	
ASSFSGADTQY	ASSGGNEQY	ASSLASYGYT	ASSLDRGSGNTIY	
ASSFSGANTEAF	ASSGGNQPQH	ASSLASYNEQF	ASSLDRGSNQPQH	
ASSLAGELF	ASSLAGPSTDTQY	ASSLATDEQY	ASSLDRGSTDTQY	
ASSLAGENTEAF	ASSLAGPTDTQY	ASSLATDTQY	ASSLDRGSYEQY	
ASSLAGEQY	ASSLAGPYEQY	ASSLATEAF	ASSLDRGSYNEQF	
ASSLAGETQY	ASSLAGQETQY	ASSLATGELF	ASSLDRGTDTQY	
ASSLAGGADTQY	ASSLAGQGYEQY	ASSLATGNQPQH	ASSLDRGTYNEQF	
ASSLAGGAYEQY	ASSLAGQNTEAF	ASSLAVNQPQH	ASSLDRGYEQY	
ASSLAGGAYNEQF	ASSLAGQPQH	ASSLAVNTEAF	ASSLDRGYGYT	
ASSLAGGEETQY	ASSLAGRETQY	ASSLAVSSYEQY	ASSLDRLNTEAF	
ASSLAGGEQY	ASSLAGRNTEAF	ASSLAVSYEQY	ASSLDRNQPQH	
ASSLAGGGETQY	ASSLAGRQETQY	ASSLAVTDTQY	ASSLDRNTEAF	
ASSLAGGGNQPQH	ASSLAGRTDTQY	ASSLAVYNEQF	ASSLDRNTIY	
ASSLAGGGTDTQY	ASSLAGSGNTIY	ASSLAYEQF	ASSLDRNYGYT	
ASSLAGGGTEAF	ASSLAGSNQPQH	ASSLDADTQY	ASSLDRSSGNTIY	
ASSLAGGHNEQF	ASSLAGSNTEAF	ASSLDDTQY	ASSLDRSSYEQY	
ASSLAGGIYEQY	ASSLAGSSYEQY	ASSLDFYEQY	ASSLDRSSYNEQF	
ASSLAGGKETQY	ASSLAGSSYNEQF	ASSLDGDTQY	ASSLDRSYEQY	
ASSLAGGLYEQY	ASSLAGSTDTQY	ASSLDGETQY	ASSLDRSYGYT	
ASSLAGGLYNEQF	ASSLAGSYEQY	ASSLDGGGTDTQY	ASSLDRTEAF	
ASSLAGGNEQF	ASSLAGSYGYT	ASSLDGGTEAF	ASSLDRVNTEAF	
ASSLAGGNQPQH	ASSLAGSYNEQF	ASSLDGNQPQH	ASSLDRVSYEQY	
ASSLAGGNTEAF	ASSLAGTDTQY	ASSLDGNSNQPQH	ASSLDRVYEQY	
ASSLAGGNTIY	ASSLAGTDYGYT	ASSLDGNTEAF	ASSLDRYEQY	
ASSLAGGPDTQY	ASSLAGTEAF	ASSLDGSSYEQY	ASSLDSGNTIY	
ASSLAGGPETQY	ASSLAGTGNQPQH	ASSLDGSSYNEQF	ASSLDSLNTEAF	
ASSLAGGPGDTQY	ASSLAGTGPYEQY	ASSLDGSYEQY	ASSLDSNEQF	
ASSLAGGPNEQF	ASSLAGTQETQY	ASSLDGTDTQY	ASSLDSNQPQH	
ASSLAGGPTDTQY	ASSLAGTSTDTQY	ASSLDGYEQY	ASSLDSNTEAF	
ASSLAGGPYEQY	ASSLAGTYEQY	ASSLDGYGYT	ASSLDSNYGYT	
ASSLAGGQETQY	ASSLAGTYNEQF	ASSLDGYSNQPQH	ASSLDSPLH	
ASSLAGGQPQH	ASSLAGVGTDTQY	ASSLDHNEQF	ASSLDSQETQY	
ASSLAGGRETQY	ASSLAGVNTEAF	ASSLDLNTEAF	ASSLDSQPQH	
ASSLAGGRTDTQY	ASSLAGVTDTQY	ASSLDLSYEQY	ASSLDSSGNTIY	
ASSLAGGSNQPQH	ASSLAGYEQY	ASSLDLYEQY	ASSLDSSNQPQH	
ASSLAGGSSYEQY	ASSLAGYGYT	ASSLDNEQF	ASSLDSSSYEQY	
ASSLAGGSSYNEQF	ASSLAGYNEQF	ASSLDNQPQH	ASSLDSSYEQY	
ASSLAGGSTDTQY	ASSLAGYSNQPQH	ASSLDNYEQY	ASSLDSSYNEQF	
ASSLAGGSYEQY	ASSLAITDTQY	ASSLDPNTEAF	ASSLDSTDTQY	
ASSLAGGSYNEQF	ASSLALDTQY	ASSLDQETQY	ASSLDSYEQY	
ASSLAGGTDTQY	ASSLALGNEQF	ASSLDRADTQY	ASSLDSYGYT	
ASSLAGGTEAF	ASSLALGTDTQY	ASSLDRANTEAF	ASSLDSYNEQF	
ASSLAGGTGELF	ASSLALNEQF	ASSLGDYGYT	ASSLGGNYGYT	
ASSLAGGTYEQY	ASSLALNTEAF	ASSLGDYNEQF	ASSLGGPDTQY	
ASSLAGGYEQY	ASSLALQETQY	ASSLGENTEAF	ASSLGGPGNTIY	
ASSLAGGYGYT	ASSLALSTDTQY	ASSLGESYEQY	ASSLGGPQETQY	
ASSLAGGYNEQF	ASSLALTDTQY	ASSLGETQY	ASSLGGPTDTQY	
ASSLAGGYT	ASSLALYEQY	ASSLGFSYEQY	ASSLGGPYEQY	
ASSLAGIQETQY	ASSLALYNEQF	ASSLGFYEQY	ASSLGGQETQY	
ASSLAGLQETQY	ASSLAMNTEAF	ASSLGGADTQY	ASSLGGQNTEAF	
ASSLAGLSTDTQY	ASSLANEQF	ASSLGGAEAF	ASSLGGQPQH	
ASSLAGLSYEQY	ASSLANQETQY	ASSLGGAGQPQH	ASSLGGQQETQY	
ASSLAGNQPQH	ASSLAPDTQY	ASSLGGASTDTQY	ASSLGGQSYEQY	
ASSLAGNSYNEQF	ASSLAPSYEQY	ASSLGGASYEQY	ASSLGGRETQY	
ASSLAGNTEAF	ASSLAPYEQY	ASSLGGATDTQY	ASSLGGRSYEQY	
ASSLAGNTGELF	ASSLAQETQY	ASSLGGATEAF	ASSLGGRSYNEQF	
ASSLAGNYGYT	ASSLAQGADTQY	ASSLGGATNEKLF	ASSLGGRTDTQY	
ASSLAGPNTEAF	ASSLAQNTEAF	ASSLGGAYEQY	ASSLGGRTEAF	
ASSLDTDTQY	ASSLERGSYNEQF	ASSLGGDQPQH	ASSLGGRYEQY	
ASSLDTGELF	ASSLESNQPQH	ASSLGGDSNQPQH	ASSLGGSGNTIY	
ASSLDTNTEAF	ASSLESNTEAF	ASSLGGDTEAF	ASSLGGSNEQF	
ASSLDTNYGYT	ASSLESTDTQY	ASSLGGDTQY	ASSLGGSNQPQH	
ASSLDTSYEQY	ASSLESTEAF	ASSLGGDYGYT	ASSLGGSNTEAF	
ASSLDTYEQY	ASSLESYEQY	ASSLGGELF	ASSLGGSSNQPQH	
ASSLDWNTEAF	ASSLETNTEAF	ASSLGGEQF	ASSLGGSSTDTQY	
ASSLDYEQY	ASSLETSTDTQY	ASSLGGEQY	ASSLGGSSYEQY	
ASSLDYQETQY	ASSLEVDQPQH	ASSLGGETQY	ASSLGGSSYNEQF	
ASSLEADTQY	ASSLEVDTQY	ASSLGGGDTQY	ASSLGGSTDTQY	
ASSLEAGNTEAF	ASSLEVNTEAF	ASSLGGGELF	ASSLGGSTEAF	
ASSLEANTEAF	ASSLEYEQY	ASSLGGGGNQPQH	ASSLGGSTGELF	
ASSLEANYGYT	ASSLEYNEQF	ASSLGGGGTDTQY	ASSLGGSYEQY	
ASSLEASYEQY	ASSLFGDTQY	ASSLGGGGTEAF	ASSLGGSYGYT	
ASSLEDSNQPQH	ASSLFGEQY	ASSLGGGNEQF	ASSLGGSYNEQF	
ASSLEDTQY	ASSLFGTEAF	ASSLGGGNQPQH	ASSLGGTDTQY	
ASSLEEETQY	ASSLFGYEQY	ASSLGGGNTEAF	ASSLGGTEAF	
ASSLEENTEAF	ASSLFLNTEAF	ASSLGGGNTIY	ASSLGGTGAYEQY	
ASSLEETQY	ASSLFNQPQH	ASSLGGGQETQY	ASSLGGTGELF	
ASSLEGANTEAF	ASSLFTEAF	ASSLGGGQPQH	ASSLGGTGNTIY	
ASSLEGDQPQH	ASSLGAANTEAF	ASSLGGGSNQPQH	ASSLGGTQETQY	
ASSLEGDTEAF	ASSLGADTEAF	ASSLGGGTDTQY	ASSLGGTQY	
ASSLEGDTQY	ASSLGADTQY	ASSLGGGTEAF	ASSLGGTYEQY	
ASSLEGEETQY	ASSLGAEAF	ASSLGGGTYEQY	ASSLGGTYGYT	
ASSLEGETQY	ASSLGAEQY	ASSLGGGYEQY	ASSLGGVGNQPQH	
ASSLEGGQPQH	ASSLGAGELF	ASSLGGGYNEQF	ASSLGGVNQPQH	
ASSLEGGSYEQY	ASSLGAGGTDTQY	ASSLGGKETQY	ASSLGGVNTEAF	
ASSLEGGTEAF	ASSLGAGNTEAF	ASSLGGLGTEAF	ASSLGGVSYEQY	
ASSLEGGYEQY	ASSLGAGQETQY	ASSLGGLNEQF	ASSLGGVTEAF	
ASSLEGLNTEAF	ASSLGAMNTEAF	ASSLGGLNTEAF	ASSLGGYEQY	
ASSLEGNEQF	ASSLGANTEAF	ASSLGGLQETQY	ASSLGGYGYT	
ASSLEGNQPQH	ASSLGANYGYT	ASSLGGLSTDTQY	ASSLGGYNEQF	
ASSLEGNTEAF	ASSLGAPNQPQH	ASSLGGMNTEAF	ASSLGGYQPQH	
ASSLEGNYGYT	ASSLGAQPQH	ASSLGGNEQF	ASSLGGYTDTQY	
ASSLEGPYEQY	ASSLGASGNTIY	ASSLGGNEQY	ASSLGGYTEAF	
ASSLEGQPQH	ASSLGASNQPQH	ASSLGGNNEQF	ASSLGHEQY	
ASSLEGRNTEAF	ASSLGASTDTQY	ASSLGGNQPQH	ASSLGHNEQF	
ASSLEGSGNTIY	ASSLGATDTQY	ASSLGGNSPLH	ASSLGHQNTEAF	
ASSLEGSNQPQH	ASSLGATEAF	ASSLGGNTEAF	ASSLGHTDTQY	
ASSLEGSNYGYT	ASSLGAYEQY	ASSLGGNTIY	ASSLGIDTQY	
ASSLEGSSYEQY	ASSLGAYNEQF	ASSLGQGSYEQY	ASSLGRMNTEAF	
ASSLEGSSYNEQF	ASSLGDEQF	ASSLGQGSYNEQF	ASSLGRNEQF	
ASSLEGSYEQY	ASSLGDEQY	ASSLGQGTDTQY	ASSLGRNQPQH	
ASSLEGSYNEQF	ASSLGDGSYEQY	ASSLGQGTEAF	ASSLGRNTEAF	
ASSLEGTDTQY	ASSLGDQPQH	ASSLGQGTYEQY	ASSLGRNYGYT	
ASSLEGTEAF	ASSLGDRAYEQY	ASSLGQGVQPQH	ASSLGRPYEQY	
ASSLEGTQY	ASSLGDREAF	ASSLGQGYEQY	ASSLGRQETQY	
ASSLEGYEQY	ASSLGDSGNTIY	ASSLGQGYGYT	ASSLGRSNQPQH	
ASSLEGYSNQPQH	ASSLGDSNQPQH	ASSLGQGYQPQH	ASSLGRSSYEQY	
ASSLEGYTEAF	ASSLGDSQETQY	ASSLGQGYTEAF	ASSLGRSSYNEQF	
ASSLELNTEAF	ASSLGDSSYEQY	ASSLGQKETQY	ASSLGRSTDTQY	
ASSLENEQF	ASSLGDSTDTQY	ASSLGQKNTEAF	ASSLGRSYEQY	
ASSLEPGELF	ASSLGDSYEQY	ASSLGQLNQPQH	ASSLGRTDTQY	
ASSLEPNTEAF	ASSLGDTEAF	ASSLGQLNSPLH	ASSLGRTYEQY	
ASSLEPSNQPQH	ASSLGDTQY	ASSLGQLNTEAF	ASSLGRYEQY	
ASSLEQNTEAF	ASSLGDYEQY	ASSLGQLQETQY	ASSLGRYNEQF	
ASSLGINQPQH	ASSLGNYGYT	ASSLGQLYEQY	ASSLGSDTQY	
ASSLGIQETQY	ASSLGPAYEQY	ASSLGQMNTEAF	ASSLGSEAF	
ASSLGISNQPQH	ASSLGPDTQY	ASSLGQNQPQH	ASSLGSEQF	
ASSLGISTDTQY	ASSLGPEQY	ASSLGQNTEAF	ASSLGSETQY	
ASSLGKNTEAF	ASSLGPETQY	ASSLGQNTGELF	ASSLGSGANVLT	
ASSLGLAADTQY	ASSLGPGELF	ASSLGQNYEQY	ASSLGSGNTIY	
ASSLGLAAYEQY	ASSLGPGTDTQY	ASSLGQNYGYT	ASSLGSGSTDTQY	
ASSLGLADNEQF	ASSLGPMNTEAF	ASSLGQPNQPQH	ASSLGSGTEAF	
ASSLGLADTQY	ASSLGPNNEQF	ASSLGQPNTEAF	ASSLGSNQPQH	
ASSLGLAEETQY	ASSLGPNQPQH	ASSLGQQETQY	ASSLGSNSNQPQH	
ASSLGLAETQY	ASSLGPNTEAF	ASSLGQSNQPQH	ASSLGSNTEAF	
ASSLGLAGANEQF	ASSLGPNYGYT	ASSLGQSSGNTIY	ASSLGSNTGELF	
ASSLGLAGATDTQY	ASSLGPQETQY	ASSLGQSSYEQY	ASSLGSPDTQY	
ASSLGLAGAYEQY	ASSLGPSNQPQH	ASSLGQSSYNEQF	ASSLGSQETQY	
ASSLGLAGGQETQY	ASSLGPSSYEQY	ASSLGQSSYNSPLH	ASSLGSQPQH	
ASSLGLAGGTDTQY	ASSLGPSSYNEQF	ASSLGQSTDTQY	ASSLGSSGNTIY	
ASSLGLAGNEQF	ASSLGPSTDTQY	ASSLGQSYEQY	ASSLGSSTDTQY	
ASSLGLAGTDTQY	ASSLGPTDTQY	ASSLGQSYGYT	ASSLGSSYEQY	
ASSLGLAGTTDTQY	ASSLGPTYEQY	ASSLGQSYNEQF	ASSLGSSYNSPLH	
ASSLGLAGVDTQY	ASSLGPYEQY	ASSLGQTNTEAF	ASSLGSTDTQY	
ASSLGLATDTQY	ASSLGPYGYT	ASSLGQTTEAF	ASSLGSTEAF	
ASSLGLAYEQY	ASSLGPYNEQF	ASSLGQTYEQY	ASSLGSTGELF	
ASSLGLDTDTQY	ASSLGQANTEAF	ASSLGQVNTEAF	ASSLGSTYEQY	
ASSLGLDTQY	ASSLGQASYEQY	ASSLGQYEQY	ASSLGSYEQY	
ASSLGLEAF	ASSLGQAYEQY	ASSLGQYNEQF	ASSLGSYNEQF	
ASSLGLEETQY	ASSLGQDYEQY	ASSLGQYNSPLH	ASSLGTAGNTIY	
ASSLGLEQY	ASSLGQEQF	ASSLGRAYEQY	ASSLGTANTEAF	
ASSLGLETQY	ASSLGQETQY	ASSLGRDNEQF	ASSLGTANYGYT	
ASSLGLGNQPQH	ASSLGQFNQPQH	ASSLGRDQPQH	ASSLGTAQETQY	
ASSLGLGSYEQY	ASSLGQGADTQY	ASSLGRDTQY	ASSLGTASNQPQH	
ASSLGLGYEQY	ASSLGQGAYEQY	ASSLGREAF	ASSLGTASYEQY	
ASSLGLLNTEAF	ASSLGQGAYNEQF	ASSLGREQF	ASSLGTATDTQY	
ASSLGLNNEQF	ASSLGQGGNQPQH	ASSLGRETQY	ASSLGTATYEQY	
ASSLGLNQPQH	ASSLGQGGQPQH	ASSLGRGLNTEAF	ASSLGTAYEQY	
ASSLGLNTEAF	ASSLGQGGSYEQY	ASSLGRGNQPQH	ASSLGTDTEAF	
ASSLGLNTGELF	ASSLGQGGTDTQY	ASSLGRGQETQY	ASSLGTDTQY	
ASSLGLNYGYT	ASSLGQGGTEAF	ASSLGRGQPQH	ASSLGTEAF	
ASSLGLQETQY	ASSLGQGGYEQY	ASSLGRGTDTQY	ASSLGTGAYEQY	
ASSLGLSNQPQH	ASSLGQGGYNEQF	ASSLGRGTEAF	ASSLGTGEETQY	
ASSLGLSSYNEQF	ASSLGQGLNTEAF	ASSLGRLNTEAF	ASSLGTGELF	
ASSLGLSTDTQY	ASSLGQGLYEQY	ASSLLGQPQH	ASSLPNEKLF	
ASSLGLTDTQY	ASSLGQGNEQF	ASSLLGSGNTIY	ASSLPNQPQH	
ASSLGLTYEQY	ASSLGQGNQPQH	ASSLLGSYEQY	ASSLPNSPLH	
ASSLGLVTDTQY	ASSLGQGNSPLH	ASSLLGTDTQY	ASSLPNTEAF	
ASSLGLYEQY	ASSLGQGNTEAF	ASSLLGTEAF	ASSLPPNTEAF	
ASSLGLYGYT	ASSLGQGNYGYT	ASSLLGYEQY	ASSLPPYEQY	
ASSLGLYNEQF	ASSLGQGPYEQY	ASSLLMNTEAF	ASSLPSSYEQY	
ASSLGMNTEAF	ASSLGQGQETQY	ASSLLNEQF	ASSLPSSYNEQF	
ASSLGNEQF	ASSLGQGQPQH	ASSLLNQPQH	ASSLPSTDTQY	
ASSLGNEQY	ASSLGQGRGYT	ASSLLNTEAF	ASSLPSYEQY	
ASSLGNNEQF	ASSLGQGRTEAF	ASSLLNTGELF	ASSLPTDTQY	
ASSLGNQPQH	ASSLGQGRYEQY	ASSLLPSYEQY	ASSLPYNEQF	
ASSLGNSPLH	ASSLGQGSNQPQH	ASSLLPYEQY	ASSLQENTEAF	
ASSLGNTEAF	ASSLGQGSQPQH	ASSLLQGNTEAF	ASSLQETQY	
ASSLGNTIY	ASSLGQGSSYEQY	ASSLLRETQY	ASSLQGAEAF	
ASSLGNYEQY	ASSLGQGSTDTQY	ASSLLSNQPQH	ASSLQGAGELF	
ASSLGTGETDTQY	ASSLGYSNQPQH	ASSLLSQETQY	ASSLQGANYGYT	
ASSLGTGGNQPQH	ASSLGYSTDTQY	ASSLLSSYNEQF	ASSLQGDEQY	
ASSLGTGGTEAF	ASSLGYTDTQY	ASSLLTDTQY	ASSLQGDQPQH	
ASSLGTGGYEQY	ASSLGYTEAF	ASSLLTGELF	ASSLQGDTEAF	
ASSLGTGGYNEQF	ASSLHSNQPQH	ASSLLVNTEAF	ASSLQGDYGYT	
ASSLGTGLYEQY	ASSLHYEQY	ASSLLYNEQF	ASSLQGEAF	
ASSLGTGNQPQH	ASSLIGDTQY	ASSLMGGTEAF	ASSLQGGGYT	
ASSLGTGNTEAF	ASSLIGGNQPQH	ASSLMGNQPQH	ASSLQGGNQPQH	
ASSLGTGQETQY	ASSLIGNEQF	ASSLMGNTEAF	ASSLQGGQPQH	
ASSLGTGSNQPQH	ASSLIGNQPQH	ASSLMGSNQPQH	ASSLQGGTDTQY	
ASSLGTGSQPQH	ASSLIGYEQY	ASSLNEKLF	ASSLQGGTEAF	
ASSLGTGTYEQY	ASSLINEQF	ASSLNGNQPQH	ASSLQGLNTEAF	
ASSLGTGYEQY	ASSLISTDTQY	ASSLNGYEQY	ASSLQGMNTEAF	
ASSLGTLNTEAF	ASSLISYEQY	ASSLNLNTEAF	ASSLQGNEQF	
ASSLGTMNTEAF	ASSLITDTQY	ASSLNNEQF	ASSLQGNQPQH	
ASSLGTNEQF	ASSLIVNTEAF	ASSLNNSPLH	ASSLQGNTEAF	
ASSLGTNTEAF	ASSLKDTQY	ASSLNPYEQY	ASSLQGNYGYT	
ASSLGTQETQY	ASSLKETQY	ASSLNQETQY	ASSLQGPNTEAF	
ASSLGTRTDTQY	ASSLKGMNTEAF	ASSLNQPQH	ASSLQGPYEQY	
ASSLGTSGGTDTQY	ASSLKGNTEAF	ASSLNRGNTEAF	ASSLQGSNQPQH	
ASSLGTSGNTIY	ASSLKGSYEQY	ASSLNRGQETQY	ASSLQGSSYEQY	
ASSLGTSGQETQY	ASSLKGTDTQY	ASSLNRNTEAF	ASSLQGSSYNEQF	
ASSLGTSGSYEQY	ASSLKGTEAF	ASSLNSNQPQH	ASSLQGSTDTQY	
ASSLGTSGYNEQF	ASSLKMNTEAF	ASSLNSYEQY	ASSLQGSTEAF	
ASSLGTSSYEQY	ASSLLADTQY	ASSLNTDTQY	ASSLQGSYEQY	
ASSLGTSSYNEQF	ASSLLAGADTQY	ASSLNTEAF	ASSLQGTDTQY	
ASSLGTSYEQY	ASSLLAGAYEQY	ASSLNTGELF	ASSLQGTGELF	
ASSLGTSYNEQF	ASSLLAGAYNEQF	ASSLNYEQY	ASSLQGTTEAF	
ASSLGTTDTQY	ASSLLAGDTDTQY	ASSLNYNEQF	ASSLQGTYEQY	
ASSLGTTEAF	ASSLLAGGSYEQY	ASSLPDTQY	ASSLQGVNTEAF	
ASSLGTTYEQY	ASSLLAGGTDTQY	ASSLPGGETQY	ASSLQGVSGNTIY	
ASSLGTVNTEAF	ASSLLAGQETQY	ASSLPGGTEAF	ASSLQGYEQY	
ASSLGTVQETQY	ASSLLAGSYNEQF	ASSLPGNTEAF	ASSLQGYNSPLH	
ASSLGTVSNQPQH	ASSLLAGTDTQY	ASSLPGQGYEQY	ASSLQGYTEAF	
ASSLGTVYEQY	ASSLLAGVYNEQF	ASSLPGSYEQY	ASSLQGYTGELF	
ASSLGTYEQY	ASSLLAQETQY	ASSLPGTDTQY	ASSLQLNTEAF	
ASSLGTYNEQF	ASSLLAVQETQY	ASSLPGTEAF	ASSLQNEQF	
ASSLGTYSNQPQH	ASSLLDTEAF	ASSLPGYEQY	ASSLQNTEAF	
ASSLGVDTQY	ASSLLDTQY	ASSLPGYGYT	ASSLQQETQY	
ASSLGVLNTEAF	ASSLLETQY	ASSLPMNTEAF	ASSLQSSYEQY	
ASSLGVNEQF	ASSLLGDEQF	ASSLSGANVLT	ASSLSGYNEQF	
ASSLGVNTEAF	ASSLLGDTEAF	ASSLSGAQETQY	ASSLSGYSNQPQH	
ASSLGVQETQY	ASSLLGDTQY	ASSLSGASTDTQY	ASSLSGYTEAF	
ASSLGVQPQH	ASSLLGEQF	ASSLSGATDTQY	ASSLSLDTEAF	
ASSLGVSGNTIY	ASSLLGETQY	ASSLSGAYEQY	ASSLSLGYEQY	
ASSLGVSNQPQH	ASSLLGGETQY	ASSLSGDTEAF	ASSLSLNTEAF	
ASSLGVSSYNEQF	ASSLLGGNTEAF	ASSLSGDYGYT	ASSLSLNYGYT	
ASSLGVSTDTQY	ASSLLGGQETQY	ASSLSGEAF	ASSLSLTDTQY	
ASSLGVSTEAF	ASSLLGGTDTQY	ASSLSGELF	ASSLSMNTEAF	
ASSLGVTEAF	ASSLLGGTEAF	ASSLSGEQY	ASSLSNEQF	
ASSLGVYEQY	ASSLLGLNTEAF	ASSLSGETQY	ASSLSNQPQH	
ASSLGWNTEAF	ASSLLGNEQF	ASSLSGFTDTQY	ASSLSNSPLH	
ASSLGYEQY	ASSLLGNQPQH	ASSLSGGAYEQY	ASSLSNTEAF	
ASSLGYGYT	ASSLLGNTEAF	ASSLSGGGGYT	ASSLSNYGYT	
ASSLGYNEQF	ASSLLGNTIY	ASSLSGGGNQPQH	ASSLSPGNTEAF	
ASSLGYQETQY	ASSLLGQETQY	ASSLSGGGTEAF	ASSLSPNTEAF	
ASSLQTNQPQH	ASSLRLAGQETQY	ASSLSGGNEKLF	ASSLSPTDTQY	
ASSLQVNTEAF	ASSLRLAGTDTQY	ASSLSGGNQPQH	ASSLSQETQY	
ASSLRADTQY	ASSLRLTDTQY	ASSLSGGNYGYT	ASSLSQGNQPQH	
ASSLRADYGYT	ASSLRNQPQH	ASSLSGGRTDTQY	ASSLSQGPYEQY	
ASSLRAEAF	ASSLRNYGYT	ASSLSGGSTDTQY	ASSLSQGSNQPQH	
ASSLRANTEAF	ASSLRPTDTQY	ASSLSGGSYEQY	ASSLSQGYEQY	
ASSLRASYEQY	ASSLRPYEQY	ASSLSGGSYNEQF	ASSLSQNTEAF	
ASSLRAYEQY	ASSLRQETQY	ASSLSGGTEAF	ASSLSRDTQY	
ASSLRDRAYEQY	ASSLRQGGTEAF	ASSLSGGTYNEQF	ASSLSRETQY	
ASSLRDRDYEQY	ASSLRQGNQPQH	ASSLSGGYEQY	ASSLSRGPYEQY	
ASSLRDSQETQY	ASSLRQGPYEQY	ASSLSGGYGYT	ASSLSRGSNQPQH	
ASSLRDSYEQY	ASSLRQGSYEQY	ASSLSGLNTEAF	ASSLSRGTEAF	
ASSLRDSYNEQF	ASSLRQGSYNEQF	ASSLSGMNTEAF	ASSLSRNEQF	
ASSLRDTDTQY	ASSLRQGTDTQY	ASSLSGNQPQH	ASSLSSEAF	
ASSLRDTEAF	ASSLRQGTEAF	ASSLSGNTEAF	ASSLSSGNTIY	
ASSLRDTQY	ASSLRQGVNTEAF	ASSLSGNTGELF	ASSLSSGQETQY	
ASSLRENQPQH	ASSLRQGYEQY	ASSLSGNTIY	ASSLSSGSYNEQF	
ASSLRENTEAF	ASSLRQGYGYT	ASSLSGNYEQY	ASSLSSNQPQH	
ASSLRETQY	ASSLRQNYGYT	ASSLSGNYGYT	ASSLSSNTEAF	
ASSLRGDEQF	ASSLRQSNQPQH	ASSLSGPYEQY	ASSLSSQETQY	
ASSLRGDTEAF	ASSLRQSYEQY	ASSLSGPYNEQF	ASSLSSSNQPQH	
ASSLRGDTQY	ASSLRQTYEQY	ASSLSGQETQY	ASSLSSSYEQY	
ASSLRGEQY	ASSLRRDTEAF	ASSLSGRNTEAF	ASSLSSSYNEQF	
ASSLRGETQY	ASSLRRNTEAF	ASSLSGSGANVLT	ASSLSSTDTQY	
ASSLRGGEQY	ASSLRRSYNEQF	ASSLSGSGNTIY	ASSLSSYEQY	
ASSLRGGETQY	ASSLRSGNTIY	ASSLSGSSYEQY	ASSLSSYNEQF	
ASSLRGGGTDTQY	ASSLRSNQPQH	ASSLSGSSYNEQF	ASSLSSYNSPLH	
ASSLRGGNEQF	ASSLRSQETQY	ASSLSGSTDTQY	ASSLSTDTQY	
ASSLRGGNTEAF	ASSLRSSYEQY	ASSLSGSYEQY	ASSLSTGELF	
ASSLRGGNTIY	ASSLRSTDTQY	ASSLSGSYNEQF	ASSLSTGNQPQH	
ASSLRGLNTEAF	ASSLRSYEQY	ASSLSGTDTQY	ASSLSVAYEQY	
ASSLRGMNTEAF	ASSLRSYGYT	ASSLSGTGNTEAF	ASSLSVNTEAF	
ASSLRGNEQY	ASSLRSYNEQF	ASSLSGTNTGELF	ASSLSVSGNTIY	
ASSLRGNQPQH	ASSLRTDTQY	ASSLSGTSTDTQY	ASSLSVSYEQY	
ASSLRGNTEAF	ASSLRTDYGYT	ASSLSGTSYEQY	ASSLSYEQY	
ASSLRGNTIY	ASSLRTEAF	ASSLSGTYEQY	ASSLSYQETQY	
ASSLRGNYEQY	ASSLRTGELF	ASSLSGTYNEQF	ASSLTAMNTEAF	
ASSLRGNYGYT	ASSLRTGTYEQY	ASSLSGVEQF	ASSLTANTEAF	
ASSLRGPDTQY	ASSLRTVNTEAF	ASSLSGYEQY	ASSLTANTGELF	
ASSLRGPTDTQY	ASSLRVNEQF	ASSLSGYGYT	ASSLTANYGYT	
ASSLRGQPQH	ASSLRVNTEAF	ASSLVGAGNTIY	ASSLVSNEQF	
ASSLRGRNQPQH	ASSLRVSYEQY	ASSLVGATDTQY	ASSLVSNTEAF	
ASSLRGRTDTQY	ASSLRYEQY	ASSLVGDGYT	ASSLVSTDTQY	
ASSLRGSGNTIY	ASSLSADTQY	ASSLVGDTQY	ASSLVSTEAF	
ASSLRGSSYEQY	ASSLSAGELF	ASSLVGEQF	ASSLVSYEQY	
ASSLRGSTDTQY	ASSLSAGGNQPQH	ASSLVGETQY	ASSLVSYNEQF	
ASSLRGSYEQY	ASSLSAGNYGYT	ASSLVGGEQY	ASSLVTDTQY	
ASSLRGSYNEQF	ASSLSAQETQY	ASSLVGGGTDTQY	ASSLVTEAF	
ASSLRGTDTQY	ASSLSAYEQY	ASSLVGGGTEAF	ASSLVTGNEQF	
ASSLRGTEAF	ASSLSAYNEQF	ASSLVGGNYGYT	ASSLVTNTEAF	
ASSLRGVNTEAF	ASSLSDSYNEQF	ASSLVGGQETQY	ASSLWGADTQY	
ASSLRGYEQF	ASSLSDTQY	ASSLVGGSTDTQY	ASSLWGDTEAF	
ASSLRGYEQY	ASSLSENTEAF	ASSLVGGSYEQY	ASSLWGNTEAF	
ASSLRGYGYT	ASSLSETQY	ASSLVGGTDTQY	ASSLWGQPQH	
ASSLRGYTEAF	ASSLSFNQPQH	ASSLVGGTEAF	ASSLWGTEAF	
ASSLRLAGGTDTQY	ASSLSGADTQY	ASSLVGGYEQY	ASSLWGYEQY	
ASSLTASYEQY	ASSLTGVTEAF	ASSLVGMNTEAF	ASSLWVNTEAF	
ASSLTAYEQY	ASSLTGYEQY	ASSLVGNEQF	ASSLYGETQY	
ASSLTDNEQF	ASSLTGYNEQF	ASSLVGNEQY	ASSLYGGNTEAF	
ASSLTDTQY	ASSLTGYQETQY	ASSLVGNTEAF	ASSLYGQPQH	
ASSLTENTEAF	ASSLTLNTEAF	ASSLVGNYGYT	ASSLYLNTEAF	
ASSLTENYGYT	ASSLTLNYGYT	ASSLVGQGYEQY	ASSLYNEQF	
ASSLTETQY	ASSLTLQETQY	ASSLVGQNTEAF	ASSLYNQPQH	
ASSLTGANTEAF	ASSLTMNTEAF	ASSLVGQQETQY	ASSLYQETQY	
ASSLTGAQETQY	ASSLTNNEQF	ASSLVGSDTQY	ASSLYRNTEAF	
ASSLTGDEQY	ASSLTNQPQH	ASSLVGSGNTIY	ASSLYSNQPQH	
ASSLTGDTEAF	ASSLTNTEAF	ASSLVGSPLH	ASSLYTDTQY	
ASSLTGDYGYT	ASSLTNTGELF	ASSLVGSSYEQY	ASSLYTEAF	
ASSLTGEAF	ASSLTPGNTEAF	ASSLVGSTDTQY	ASSLYTGELF	
ASSLTGELF	ASSLTPNTEAF	ASSLVGSYEQY	ASSLYYEQY	
ASSLTGEQF	ASSLTQETQY	ASSLVGSYNEQF	ASSMTENTEAF	
ASSLTGEQY	ASSLTQNTEAF	ASSLVGTDTQY	ASSMTVNTEAF	
ASSLTGGEQY	ASSLTRNTEAF	ASSLVGYEQY	ASSNQETQY	
ASSLTGGGNTIY	ASSLTSDTQY	ASSLVGYNEQF	ASSNRENTEAF	
ASSLTGGGTEAF	ASSLTSGAYEQY	ASSLVLDTQY	ASSNSYEQY	
ASSLTGGMNTEAF	ASSLTSGGYNEQF	ASSLVLNTEAF	ASSNTDTQY	
ASSLTGGNQPQH	ASSLTSGPYNEQF	ASSLVLTDTQY	ASSNYNEQF	
ASSLTGGNTEAF	ASSLTSGQETQY	ASSLVNEQF	ASSPADTQY	
ASSLTGGNYGYT	ASSLTSGSYEQY	ASSLVNSPLH	ASSPAGDTQY	
ASSLTGGSTDTQY	ASSLTSGSYNEQF	ASSLVPDTQY	ASSPAGGNQPQH	
ASSLTGGSYEQY	ASSLTSGTDTQY	ASSLVPNTEAF	ASSPAGGSYEQY	
ASSLTGGSYNEQF	ASSLTSGYEQY	ASSLVQETQY	ASSPAGNTEAF	
ASSLTGGTDTQY	ASSLTSNQPQH	ASSLVQGAYEQY	ASSPAGNTIY	
ASSLTGGTEAF	ASSLTSSYNEQF	ASSLVQGSYEQY	ASSPAGTDTQY	
ASSLTGGYEQY	ASSLTSTDTQY	ASSLVQGTNTEAF	ASSPASYEQY	
ASSLTGGYGYT	ASSLTSYEQY	ASSLVQGYEQY	ASSPAYEQY	
ASSLTGINQPQH	ASSLTSYNEQF	ASSLVRDTQY	ASSPDFYEQY	
ASSLTGIYGYT	ASSLTTAYEQY	ASSLVRETQY	ASSPDGDTQY	
ASSLTGKETQY	ASSLTTDTQY	ASSLVRGEQY	ASSPDGNTEAF	
ASSLTGLNTEAF	ASSLTTEAF	ASSLVRNEQF	ASSPDGNYGYT	
ASSLTGMNTEAF	ASSLTTGSYEQY	ASSLVRNTEAF	ASSPDGSNQPQH	
ASSLTGNEQF	ASSLTVNQPQH	ASSLVRTDTQY	ASSPDGSSGNTIY	
ASSLTGNQPQH	ASSLTVNTEAF	ASSLVRTEAF	ASSPDGSSYNEQF	
ASSLTGNTEAF	ASSLTVSNQPQH	ASSLVSDTQY	ASSPDGSYEQY	
ASSLTGNTGELF	ASSLTVSYEQY	ASSLVSEAF	ASSPDGYEQY	
ASSLTGNYEQY	ASSLTVTDTQY	ASSLVSGNEQF	ASSPDLNTEAF	
ASSLTGPNYGYT	ASSLTYEQY	ASSPGLAGAYEQY	ASSPGQGGNEQF	
ASSLTGPTDTQY	ASSLTYNEQF	ASSPGLAGDTQY	ASSPGQGGSPLH	
ASSLTGRNQPQH	ASSLVADTQY	ASSPGLAGGTDTQY	ASSPGQGGTDTQY	
ASSLTGSGNTIY	ASSLVADYEQY	ASSPGLAGNNEQF	ASSPGQGGTEAF	
ASSLTGSNQPQH	ASSLVAGELF	ASSPGLAGPYEQY	ASSPGQGGYEQY	
ASSLTGSQETQY	ASSLVAGGTDTQY	ASSPGLAGSTDTQY	ASSPGQGGYNEQF	
ASSLTGSSYNEQF	ASSLVAGGYNEQF	ASSPGLAGTDTQY	ASSPGQGISYEQY	
ASSLTGSTEAF	ASSLVASTDTQY	ASSPGLAGYNEQF	ASSPGQGLNTEAF	
ASSLTGSYEQY	ASSLVAYEQY	ASSPGLAQETQY	ASSPGQGNEKLF	
ASSLTGSYGYT	ASSLVDEQF	ASSPGLASDTQY	ASSPGQGNQPQH	
ASSLTGTEAF	ASSLVDNQPQH	ASSPGLASTDTQY	ASSPGQGNSPLH	
ASSLTGTSYEQY	ASSLVDSSYEQY	ASSPGLATDTQY	ASSPGQGNTEAF	
ASSLTGTYEQY	ASSLVDTGELF	ASSPGLAVYNEQF	ASSPGQGNYGYT	
ASSLTGVETQY	ASSLVDTQY	ASSPGLAYEQY	ASSPGQGPYEQY	
ASSLTGVGQPQH	ASSLVEETQY	ASSPGLEETQY	ASSPGQGRTDTQY	
ASSLTGVQPQH	ASSLVENTEAF	ASSPGLGTDTQY	ASSPGQGRYEQY	
ASSPDNEQF	ASSPGASTDTQY	ASSPGLGYEQY	ASSPGQGSNQPQH	
ASSPDNQPQH	ASSPGAYEQY	ASSPGLMNTEAF	ASSPGQGSTDTQY	
ASSPDRANTEAF	ASSPGDEQY	ASSPGLNTEAF	ASSPGQGSYEQY	
ASSPDRASGNTIY	ASSPGDGYT	ASSPGLQETQY	ASSPGQGSYNEQF	
ASSPDRAYEQY	ASSPGDTDTQY	ASSPGLSNQPQH	ASSPGQGTDTQY	
ASSPDRAYGYT	ASSPGDTEAF	ASSPGLSSYNEQF	ASSPGQGTYEQY	
ASSPDRDEQY	ASSPGDTQY	ASSPGLSTDTQY	ASSPGQGVYEQY	
ASSPDRDQPQH	ASSPGDYGYT	ASSPGLSYEQY	ASSPGQGYEQY	
ASSPDRDYGYT	ASSPGDYNEQF	ASSPGLTYEQY	ASSPGQGYGYT	
ASSPDREETQY	ASSPGEETQY	ASSPGLYEQY	ASSPGQGYNEQF	
ASSPDRGETQY	ASSPGETQY	ASSPGLYNEQF	ASSPGQGYQETQY	
ASSPDRGNQPQH	ASSPGFYEQY	ASSPGMNTEAF	ASSPGQIYEQY	
ASSPDRGPYEQY	ASSPGGAQETQY	ASSPGNEQY	ASSPGQIYGYT	
ASSPDRGQPQH	ASSPGGDTQY	ASSPGNQETQY	ASSPGQKETQY	
ASSPDRGSYNEQF	ASSPGGEETQY	ASSPGNQPQH	ASSPGQLNTEAF	
ASSPDRGTDTQY	ASSPGGEQF	ASSPGNSNQPQH	ASSPGQLYGYT	
ASSPDRGYEQY	ASSPGGEQY	ASSPGNSPLH	ASSPGQMNTEAF	
ASSPDRGYGYT	ASSPGGETQY	ASSPGNSYEQY	ASSPGQNNEQF	
ASSPDRLNTEAF	ASSPGGGEQY	ASSPGNTEAF	ASSPGQNNQPQH	
ASSPDRNQPQH	ASSPGGGGYT	ASSPGNTIY	ASSPGQNNSPLH	
ASSPDRNTEAF	ASSPGGGQPQH	ASSPGPEAF	ASSPGQNQPQH	
ASSPDRNYGYT	ASSPGGGTDTQY	ASSPGPHNEQF	ASSPGQNTEAF	
ASSPDRSSYNEQF	ASSPGGGTEAF	ASSPGPQETQY	ASSPGQNYGYT	
ASSPDRTYEQY	ASSPGGGYEQY	ASSPGPSYEQY	ASSPGQPYEQY	
ASSPDRVNQPQH	ASSPGGGYGYT	ASSPGPTDTQY	ASSPGQQETQY	
ASSPDRVNTEAF	ASSPGGGYNEQF	ASSPGPYEQY	ASSPGQRNTEAF	
ASSPDRVQPQH	ASSPGGGYT	ASSPGPYGYT	ASSPGQSNQPQH	
ASSPDRYEQY	ASSPGGLNTEAF	ASSPGPYNEQF	ASSPGQSSYEQY	
ASSPDRYNEQF	ASSPGGMNTEAF	ASSPGQAYEQY	ASSPGQSTDTQY	
ASSPDSNQPQH	ASSPGGNEQF	ASSPGQDTGELF	ASSPGQSYEQY	
ASSPDSNYGYT	ASSPGGNQPQH	ASSPGQDYGYT	ASSPGQTSTDTQY	
ASSPDSQETQY	ASSPGGNTEAF	ASSPGQETQY	ASSPGQTYEQY	
ASSPDSSGNTIY	ASSPGGNTIY	ASSPGQFYEQY	ASSPGQTYGYT	
ASSPDSSNQPQH	ASSPGGPDTQY	ASSPGQGAGEQY	ASSPGQVNTEAF	
ASSPDSSYNEQF	ASSPGGQETQY	ASSPGQGARTEAF	ASSPGQVSNQPQH	
ASSPDSTDTQY	ASSPGGRETQY	ASSPGQGASYEQY	ASSPGQVSYEQY	
ASSPDSYEQY	ASSPGGSNQPQH	ASSPGQGAYEQY	ASSPGQVYGYT	
ASSPDSYGYT	ASSPGGSNTEAF	ASSPGQGAYGYT	ASSPGQYNEQF	
ASSPDSYNEQF	ASSPGGSSYNEQF	ASSPGQGDGYT	ASSPGQYNSPLH	
ASSPDSYSNQPQH	ASSPGGSTDTQY	ASSPGQGDTEAF	ASSPGQYQETQY	
ASSPDTEAF	ASSPGGSYEQY	ASSPLNEQF	ASSPPSYNEQF	
ASSPDTGELF	ASSPGGSYGYT	ASSPLNTEAF	ASSPPTDTQY	
ASSPDTNTEAF	ASSPGGSYNEQF	ASSPLQETQY	ASSPPTGMNTEAF	
ASSPDTYEQY	ASSPGGTDTQY	ASSPLRDTQY	ASSPPYQETQY	
ASSPDYEQY	ASSPGGTEAF	ASSPLRNTEAF	ASSPQANTEAF	
ASSPEETQY	ASSPGGTGNTIY	ASSPLSNQPQH	ASSPQDTQY	
ASSPEGYEQY	ASSPGGTQY	ASSPLSSYEQY	ASSPQETQY	
ASSPFQETQY	ASSPGGVGTEAF	ASSPLSSYNEQF	ASSPQGAEAF	
ASSPFTDTQY	ASSPGGYEQY	ASSPLSTDTQY	ASSPQGAGTEAF	
ASSPGADTQY	ASSPGGYGYT	ASSPLSYNEQF	ASSPQGAYEQY	
ASSPGAEAF	ASSPGGYNEQF	ASSPLTDTQY	ASSPQGDTEAF	
ASSPGAETQY	ASSPGGYYEQY	ASSPLTGGYGYT	ASSPQGETQY	
ASSPGAGELF	ASSPGLAADTQY	ASSPLYEQY	ASSPQGGEQY	
ASSPGAGNQPQH	ASSPGLAAYNEQF	ASSPMGGTEAF	ASSPQGGNSPLH	
ASSPGANTEAF	ASSPGLAEETQY	ASSPMNTEAF	ASSPQGGNTEAF	
ASSPGANYGYT	ASSPGLAGANEQF	ASSPNLNTEAF	ASSPQGGTEAF	
ASSPGQYYEQY	ASSPGTQETQY	ASSPNQETQY	ASSPQGLNTEAF	
ASSPGRGNTEAF	ASSPGTSGNNEQF	ASSPNSNQPQH	ASSPQGMNTEAF	
ASSPGRNQPQH	ASSPGTSGNTIY	ASSPNSYEQY	ASSPQGNEQF	
ASSPGRSNQPQH	ASSPGTSGQETQY	ASSPNTDTQY	ASSPQGNEQY	
ASSPGRTDTQY	ASSPGTSGSTDTQY	ASSPNTEAF	ASSPQGNQPQH	
ASSPGRYEQY	ASSPGTSGSYEQY	ASSPNTGELF	ASSPQGNTEAF	
ASSPGSDTQY	ASSPGTSGTDTQY	ASSPNYEQY	ASSPQGNYGYT	
ASSPGSGNTIY	ASSPGTSGTYEQY	ASSPNYNEQF	ASSPQGRETQY	
ASSPGSNQPQH	ASSPGTSGYEQY	ASSPPADTQY	ASSPQGSTDTQY	
ASSPGSQETQY	ASSPGTSGYNEQF	ASSPPGADTQY	ASSPQGSTEAF	
ASSPGSSGNTIY	ASSPGTSQETQY	ASSPPGANVLT	ASSPQGSYEQY	
ASSPGSSYEQY	ASSPGTSSYNEQF	ASSPPGAYEQY	ASSPQGTGNTIY	
ASSPGSSYNEQF	ASSPGTSTDTQY	ASSPPGDTQY	ASSPQGTYEQY	
ASSPGSTDTQY	ASSPGTSYEQY	ASSPPGETQY	ASSPQGVSNQPQH	
ASSPGSYEQY	ASSPGTTSTDTQY	ASSPPGGETQY	ASSPQGYEQY	
ASSPGTAEAF	ASSPGTTYNEQF	ASSPPGGNYGYT	ASSPQNTEAF	
ASSPGTAGNTIY	ASSPGTVNQPQH	ASSPPGGSYEQY	ASSPQQETQY	
ASSPGTANTEAF	ASSPGTVNYGYT	ASSPPGGYEQY	ASSPQRGTEAF	
ASSPGTANYGYT	ASSPGTVSYEQY	ASSPPGLAYEQY	ASSPQRNTEAF	
ASSPGTASYEQY	ASSPGTVYGYT	ASSPPGLYEQY	ASSPRADTQY	
ASSPGTATDTQY	ASSPGTYEQY	ASSPPGNEQF	ASSPRAYEQY	
ASSPGTAYEQY	ASSPGTYNEQF	ASSPPGNTIY	ASSPRDRAYEQY	
ASSPGTAYGYT	ASSPGVGYT	ASSPPGQPYEQY	ASSPRDREETQY	
ASSPGTAYNEQF	ASSPGVNTEAF	ASSPPGRQETQY	ASSPRDRGYEQY	
ASSPGTDNQPQH	ASSPGVSYEQY	ASSPPGSSYEQY	ASSPRDRSSYEQY	
ASSPGTDSNQPQH	ASSPGYEQY	ASSPPGSYEQY	ASSPRDSNQPQH	
ASSPGTDTQY	ASSPGYNEQF	ASSPPGTDTQY	ASSPRDSPYEQY	
ASSPGTGAYEQY	ASSPGYQETQY	ASSPPGTEAF	ASSPRDSSYEQY	
ASSPGTGELF	ASSPGYSNQPQH	ASSPPGTYEQY	ASSPRDSSYNEQF	
ASSPGTGFEQY	ASSPHNEQF	ASSPPMNTEAF	ASSPRDSYEQY	
ASSPGTGGADTQY	ASSPHTDTQY	ASSPPNTEAF	ASSPRDTEAF	
ASSPGTGGNQPQH	ASSPHYEQY	ASSPPNTGELF	ASSPREETQY	
ASSPGTGGTDTQY	ASSPKADTQY	ASSPPPSYEQY	ASSPRETQY	
ASSPGTGGTEAF	ASSPKDTQY	ASSPPSDTQY	ASSPRGDTQY	
ASSPGTGGYEQY	ASSPKETQY	ASSPPSGQETQY	ASSPRGETQY	
ASSPGTGGYNEQF	ASSPKYEQY	ASSPPSNTEAF	ASSPRGGGTEAF	
ASSPGTGLNTEAF	ASSPLAGGLDTQY	ASSPPSSTDTQY	ASSPRGGLNTEAF	
ASSPGTGLYNEQF	ASSPLAGGPTDTQY	ASSPPSSYEQY	ASSPRGGQETQY	
ASSPGTGNQPQH	ASSPLAGGTDTQY	ASSPPSTDTQY	ASSPRGNEQF	
ASSPGTGNTEAF	ASSPLAGGYNEQF	ASSPPSYEQY	ASSPRGNTEAF	
ASSPGTGNYGYT	ASSPLAGQETQY	ASSPTGGSYEQY	ASSQAGSSYEQY	
ASSPGTGPYEQY	ASSPLDEQF	ASSPTGGSYNEQF	ASSQAGSTDTQY	
ASSPGTGQETQY	ASSPLDSTDTQY	ASSPTGGTYEQY	ASSQAGTEAF	
ASSPGTGRYEQY	ASSPLETQY	ASSPTGGYEQY	ASSQANEQF	
ASSPGTGSYEQY	ASSPLGETQY	ASSPTGGYGYT	ASSQAVNTEAF	
ASSPGTGSYNEQF	ASSPLGGDTQY	ASSPTGMNTEAF	ASSQAYEQY	
ASSPGTGTDTQY	ASSPLGGNTEAF	ASSPTGNEQF	ASSQDGETQY	
ASSPGTGTYEQY	ASSPLGGQPQH	ASSPTGNEQY	ASSQDGNTEAF	
ASSPGTGTYNEQF	ASSPLGGYEQY	ASSPTGNTEAF	ASSQDGTDTQY	
ASSPGTGYGYT	ASSPLGNEQF	ASSPTGNYGYT	ASSQDGYEQY	
ASSPGTGYNEQF	ASSPLGNQPQH	ASSPTGRETQY	ASSQDLGQPQH	
ASSPGTLNTEAF	ASSPLGNTEAF	ASSPTGSEQY	ASSQDLGSGNTIY	
ASSPGTLNTGELF	ASSPLGSYEQY	ASSPTGSNQPQH	ASSQDLGYEQY	
ASSPGTLYEQY	ASSPLGTDTQY	ASSPTGSSYEQY	ASSQDLNQPQH	
ASSPGTMNTEAF	ASSPLGYEQY	ASSPTGSYEQY	ASSQDLNTEAF	
ASSPGTPNTEAF	ASSPLLNTEAF	ASSPTGTEAF	ASSQDNEQF	
ASSPRGNTIY	ASSPSAYEQY	ASSPTGTGGYEQY	ASSQDNQPQH	
ASSPRGNYGYT	ASSPSDTQY	ASSPTGTYEQY	ASSQDPNQPQH	
ASSPRGQETQY	ASSPSENTEAF	ASSPTGVNQPQH	ASSQDRANQPQH	
ASSPRGQPQH	ASSPSGANVLT	ASSPTGVNTEAF	ASSQDRAYEQY	
ASSPRGRETQY	ASSPSGETQY	ASSPTGVYGYT	ASSQDRDQPQH	
ASSPRGSGNTIY	ASSPSGGQETQY	ASSPTGYEQY	ASSQDRDTQY	
ASSPRGSNQPQH	ASSPSGGSSYEQY	ASSPTGYTEAF	ASSQDRETQY	
ASSPRGSSYEQY	ASSPSGGYEQY	ASSPTLNTEAF	ASSQDRGEQY	
ASSPRGSSYNEQF	ASSPSGNEQF	ASSPTNQPQH	ASSQDRGETQY	
ASSPRGSYNEQF	ASSPSGNQPQH	ASSPTNYEQY	ASSQDRGLNTEAF	
ASSPRGTDTQY	ASSPSGNTIY	ASSPTPSSYNEQF	ASSQDRGNQPQH	
ASSPRGTEAF	ASSPSGQGSYEQY	ASSPTQETQY	ASSQDRGPDTQY	
ASSPRGYEQY	ASSPSGQPQH	ASSPTRNEQF	ASSQDRGSNQPQH	
ASSPRGYNEQF	ASSPSGSSYNEQF	ASSPTSGGQETQY	ASSQDRGSYEQY	
ASSPRHQETQY	ASSPSGSTDTQY	ASSPTSGGTDTQY	ASSQDRGTDTQY	
ASSPRLAGDNEQF	ASSPSGSYEQY	ASSPTSGGYEQY	ASSQDRGYGYT	
ASSPRLAGGTDTQY	ASSPSGTYNEQF	ASSPTSGQETQY	ASSQDRNQPQH	
ASSPRLAGSTDTQY	ASSPSGYEQY	ASSPTSGSYEQY	ASSQDRNSPLH	
ASSPRLNTEAF	ASSPSGYNEQF	ASSPTSGSYNEQF	ASSQDRNTEAF	
ASSPRMNTEAF	ASSPSMNTEAF	ASSPTSGVYNEQF	ASSQDRQETQY	
ASSPRNTEAF	ASSPSNQPQH	ASSPTSTDTQY	ASSQDRRETQY	
ASSPRQETQY	ASSPSNTEAF	ASSPTSYEQY	ASSQDRSSYNEQF	
ASSPRQGGTEAF	ASSPSNYGYT	ASSPTSYNEQF	ASSQDRSYEQY	
ASSPRQGGYEQY	ASSPSRETQY	ASSPTTDTQY	ASSQDRVNTEAF	
ASSPRQGLSYEQY	ASSPSRSYEQY	ASSPTVNTEAF	ASSQDSAYEQY	
ASSPRQGNSPLH	ASSPSSDTQY	ASSPTYEQY	ASSQDSNQPQH	
ASSPRQGNTEAF	ASSPSSSYEQY	ASSPVGGTEAF	ASSQDSNYGYT	
ASSPRQGSYEQY	ASSPSSSYNEQF	ASSPVNQPQH	ASSQDSSNQPQH	
ASSPRQGTEAF	ASSPSSTDTQY	ASSPVNTEAF	ASSQDSSYEQY	
ASSPRQGTYEQY	ASSPSSYEQY	ASSPVSGNTIY	ASSQDSYEQY	
ASSPRQGYGYT	ASSPSSYNEQF	ASSPYQETQY	ASSQDSYNEQF	
ASSPRQNTEAF	ASSPSTDTQY	ASSPYSNQPQH	ASSQDTYEQY	
ASSPRQSTDTQY	ASSPSVTYEQY	ASSPYTDTQY	ASSQEETQY	
ASSPRRDQPQH	ASSPSYEQY	ASSQAGEQY	ASSQEGSDTQY	
ASSPRRDTEAF	ASSPSYNEQF	ASSQAGETQY	ASSQETGSYEQY	
ASSPRRGETQY	ASSPTANTEAF	ASSQAGGAYNEQF	ASSQEVNYGYT	
ASSPRSNQPQH	ASSPTANYGYT	ASSQAGGSYEQY	ASSQGAEAF	
ASSPRSTDTQY	ASSPTAYEQY	ASSQAGGTDTQY	ASSQGDEQF	
ASSPRTDTQY	ASSPTDTQY	ASSQAGGYT	ASSQGDEQY	
ASSPRTEAF	ASSPTENTEAF	ASSQAGNEQF	ASSQGDTQY	
ASSPRTGAYEQY	ASSPTGADTQY	ASSQTVNTEAF	ASSRDSYEQY	
ASSPRTGGTDTQY	ASSPTGAEAF	ASSQTYEQY	ASSRDSYNEQF	
ASSPRTGGTEAF	ASSPTGANTEAF	ASSQVEETQY	ASSRDTEAF	
ASSPRTGGYEQY	ASSPTGAYEQY	ASSQVGETQY	ASSRDTSYEQY	
ASSPRTGLNTEAF	ASSPTGDEQY	ASSQVGGTGELF	ASSRDTYNEQF	
ASSPRTGNTEAF	ASSPTGDQPQH	ASSQVGNQPQH	ASSRDYGYT	
ASSPRTGSSYEQY	ASSPTGDSNQPQH	ASSQVGQETQY	ASSREETQY	
ASSPRTGSYEQY	ASSPTGDTEAF	ASSQVGSNQPQH	ASSRETQY	
ASSPRTGTDTQY	ASSPTGELF	ASSQVGSTDTQY	ASSRGADTQY	
ASSPRTQETQY	ASSPTGEQY	ASSQVGYEQY	ASSRGDTQY	
ASSPRTSGTDTQY	ASSPTGETQY	ASSQVGYGYT	ASSRGDYGYT	
ASSPRTVQETQY	ASSPTGFYGYT	ASSQVNTGELF	ASSRGETQY	
ASSPRTVSYEQY	ASSPTGGDGYT	ASSQVPDTQY	ASSRGGNTEAF	
ASSPRTYEQY	ASSPTGGGQPQH	ASSQVSDTQY	ASSRGGSNQPQH	
ASSPRVNTEAF	ASSPTGGGTEAF	ASSQVSNTEAF	ASSRGGTEAF	
ASSPRYNEQF	ASSPTGGNQPQH	ASSQVSYEQY	ASSRGLAGTDTQY	
ASSQGENTEAF	ASSQGRRNTEAF	ASSQVTDTQY	ASSRGLNTEAF	
ASSQGETQY	ASSQGRSYEQY	ASSQVVNTEAF	ASSRGLQETQY	
ASSQGGAYNEQF	ASSQGRYNEQF	ASSQVYEQY	ASSRGLYNEQF	
ASSQGGDTQY	ASSQGSEAF	ASSQYNEQF	ASSRGNEQF	
ASSQGGELF	ASSQGSEQY	ASSRADTQY	ASSRGNQPQH	
ASSQGGEQY	ASSQGSGNTIY	ASSRAGNTEAF	ASSRGPDTQY	
ASSQGGGNQPQH	ASSQGSNQPQH	ASSRDGNQPQH	ASSRGPNTEAF	
ASSQGGGTEAF	ASSQGSSGNTIY	ASSRDNEQF	ASSRGPYNEQF	
ASSQGGNEQF	ASSQGSSYEQY	ASSRDNSPLH	ASSRGQETQY	
ASSQGGNQPQH	ASSQGSTDTQY	ASSRDQETQY	ASSRGQGDTEAF	
ASSQGGNTEAF	ASSQGSYEQY	ASSRDRANTEAF	ASSRGQGNQPQH	
ASSQGGNTIY	ASSQGSYNEQF	ASSRDRASYEQY	ASSRGQGNTEAF	
ASSQGGNYGYT	ASSQGTAEAF	ASSRDRAYEQY	ASSRGQGSYEQY	
ASSQGGRTEAF	ASSQGTAYEQY	ASSRDRDQPQH	ASSRGQGYEQY	
ASSQGGSGNTIY	ASSQGTEAF	ASSRDRDSPLH	ASSRGQLNTEAF	
ASSQGGSNQPQH	ASSQGTGELF	ASSRDRDTEAF	ASSRGQNTEAF	
ASSQGGSYEQY	ASSQGTGGYEQY	ASSRDRGGTEAF	ASSRGSNQPQH	
ASSQGGTDTQY	ASSQGTGNTEAF	ASSRDRGNTEAF	ASSRGSSYNEQF	
ASSQGGTEAF	ASSQGTSYEQY	ASSRDRGSNQPQH	ASSRGTDTQY	
ASSQGGTGELF	ASSQGTYEQY	ASSRDRGSYEQY	ASSRGTEAF	
ASSQGGVNTEAF	ASSQGTYGYT	ASSRDRGSYNEQF	ASSRGTSYEQY	
ASSQGGYEQY	ASSQGVNTEAF	ASSRDRGTDTQY	ASSRGYEQY	
ASSQGGYGYT	ASSQGWNTEAF	ASSRDRGTEAF	ASSRIQETQY	
ASSQGLAGTDTQY	ASSQGYEQF	ASSRDRGYEQY	ASSRLAGAYNEQF	
ASSQGLAGYEQY	ASSQGYEQY	ASSRDRGYGYT	ASSRLAGGSYNEQF	
ASSQGLAYEQY	ASSQGYGYT	ASSRDRGYNEQF	ASSRLAGGTDTQY	
ASSQGLNTEAF	ASSQGYQETQY	ASSRDRNQPQH	ASSRLAGGYNEQF	
ASSQGLSNQPQH	ASSQGYSNQPQH	ASSRDRNTEAF	ASSRLETQY	
ASSQGLSYEQY	ASSQGYTEAF	ASSRDRNYEQY	ASSRLNTEAF	
ASSQGLYNEQF	ASSQLYEQY	ASSRDRQETQY	ASSRNEQF	
ASSQGMNTEAF	ASSQLYNEQF	ASSRDRSYEQY	ASSRNNEQF	
ASSQGNEQF	ASSQNTEAF	ASSRDRTDTQY	ASSRNSNQPQH	
ASSQGNQPQH	ASSQNTGELF	ASSRDSNQPQH	ASSRNSYEQY	
ASSQGNSPLH	ASSQNYEQY	ASSRDSPYEQY	ASSRNYEQY	
ASSQGNTEAF	ASSQNYGYT	ASSRDSQETQY	ASSRNYGYT	
ASSQGNYEQY	ASSQPNQPQH	ASSRDSSSYNEQF	ASSRPNTEAF	
ASSQGPNQPQH	ASSQQENTEAF	ASSRDSSYEQY	ASSRPQETQY	
ASSQGPNTEAF	ASSQQETQY	ASSRDSSYNEQF	ASSRQENTEAF	
ASSQGPQETQY	ASSQQGGNQPQH	ASSRDSTDTQY	ASSRQETQY	
ASSQGPYGYT	ASSQQGSTEAF	ASSRDSTYEQY	ASSRQGADTQY	
ASSQGQGADTQY	ASSQQYEQY	ASSSGGNQPQH 	ASSSGQYSNQPQH 	
ASSQGQGGTEAF	ASSQRADTQY	ASSSGGNTEAF 	ASSSGRNQPQH 	
ASSQGQGNTEAF	ASSQRGSTDTQY	ASSSGGPYEQY 	ASSSGSGNTIY 	
ASSQGQGSYEQY	ASSQRSTDTQY	ASSSGGSNQPQH 	ASSSGSNQPQH 	
ASSQGQGTYEQY	ASSQRTGSYEQY	ASSSGGSSYNEQF 	ASSSGSNTEAF 	
ASSQGQGYEQY	ASSQSNEQF	ASSSGGSTDTQY 	ASSSGSSYEQY 	
ASSQGQGYNEQF	ASSQSNTEAF	ASSSGGSYEQY 	ASSSGSSYNEQF 	
ASSQGQLNTEAF	ASSQSSYEQY	ASSSGGYEQY 	ASSSGSTDTQY 	
ASSQGQNTEAF	ASSQSSYNEQF	ASSSGLADYNEQF 	ASSSGSYEQY 	
ASSQGQSYEQY	ASSQSYEQY	ASSSGLAEETQY 	ASSSGTASYEQY 	
ASSQGQTYEQY	ASSQTGGNTEAF	ASSSGLAETQY 	ASSSGTDTQY 	
ASSQGREQY	ASSQTGSYEQY	ASSSGLAGGTDTQY 	ASSSGTEAF 	
ASSQGRGYT	ASSQTGYEQY	ASSSGLAGSTDTQY 	ASSSGTGAYEQY 	
ASSQGRLNTEAF	ASSQTGYGYT	ASSSGLAGTDTQY 	ASSSGTGGTDTQY 	
ASSQGRNQPQH	ASSQTGYYGYT	ASSSGLAGVNEQF 	ASSSGTGNTEAF 	
ASSQGRNTEAF	ASSQTNTEAF	ASSSGLNTEAF 	ASSSGTGPYEQY 	
ASSRQGDQPQH	ASSRTGNYGYT	ASSSGLQETQY 	ASSSGTGSYEQY 	
ASSRQGGNQPQH	ASSRTGPYEQY	ASSSGLSNQPQH 	ASSSGTGYEQY 	
ASSRQGGNTEAF	ASSRTGSTDTQY	ASSSGLSSYEQY 	ASSSGTSDYNEQF 	
ASSRQGGTEAF	ASSRTGSTEAF	ASSSGLSYEQY 	ASSSGTSYEQY 	
ASSRQGLNTEAF	ASSRTGSYEQY	ASSSGLYNEQF 	ASSSGTTDTQY 	
ASSRQGNQPQH	ASSRTGTYEQY	ASSSGNEQF 	ASSSGTYEQY 	
ASSRQGNTEAF	ASSRTGVNTEAF	ASSSGNSPLH 	ASSSGTYNEQF 	
ASSRQGPNTEAF	ASSRTGVYEQY	ASSSGNTEAF 	ASSSGVNQPQH 	
ASSRQGQETQY	ASSRTGYEQY	ASSSGNTIY 	ASSSGVSNQPQH 	
ASSRQGSGNTIY	ASSRTIQETQY	ASSSGNYGYT 	ASSSGVYNEQF 	
ASSRQGSNQPQH	ASSRTNEQF	ASSSGPYEQY 	ASSSGYEQY 	
ASSRQGSTEAF	ASSRTSDEQF	ASSSGPYGYT 	ASSSGYGYT 	
ASSRQGSYEQY	ASSRTSGGTDTQY	ASSSGPYNEQF 	ASSSGYSNQPQH 	
ASSRQGSYNEQF	ASSRTSSYEQY	ASSSGQETQY 	ASSSHNEQF 	
ASSRQGTEAF	ASSRTSTDTQY	ASSSGQGADTQY 	ASSSHYEQY 	
ASSRQGVNTEAF	ASSRTSYEQY	ASSSGQGAYEQY 	ASSSITDTQY 	
ASSRQLNTEAF	ASSRTVNTEAF	ASSSGQGDYEQY 	ASSSKETQY 	
ASSRQMNTEAF	ASSRTVQETQY	ASSSGQGGTDTQY 	ASSSLAGASTDTQY 	
ASSRQNTEAF	ASSRTYEQY	ASSSGQGGTEAF 	ASSSLAGAYNEQF 	
ASSRQPQETQY	ASSRVGNQPQH	ASSSGQGLSYEQY 	ASSSLAGGTDTQY 	
ASSRQVNTEAF	ASSRVSSYEQY	ASSSGQGMNTEAF 	ASSSLAGSYNEQF 	
ASSRRGEQY	ASSRVSYEQY	ASSSGQGNQPQH 	ASSSLDTQY 	
ASSRRGNTEAF	ASSRWNTEAF	ASSSGQGPYEQY 	ASSSLGDTQY 	
ASSRRGTEAF	ASSRYQETQY	ASSSGQGQETQY 	ASSSLGEQY 	
ASSRRNTEAF	ASSRYSNQPQH	ASSSGQGRTDTQY 	ASSSLGYEQY 	
ASSRRTDTQY	ASSSADTQY	ASSSGQGSNQPQH 	ASSSLNQPQH 	
ASSRSDTQY	ASSSAGGSYNEQF	ASSSGQGSTDTQY 	ASSSLNTEAF 	
ASSRSGANVLT	ASSSAGPYEQY	ASSSGQGSYNEQF 	ASSSLQETQY 	
ASSRSGSYNEQF	ASSSAYEQY	ASSSGQGTYEQY 	ASSSLSTDTQY 	
ASSRSNQPQH	ASSSDGSNQPQH	ASSSGQGYEQY 	ASSSLTDTQY 	
ASSRSNTEAF	ASSSDRAYEQY	ASSSGQLNTEAF 	ASSSLYEQY 	
ASSRSSGNTIY	ASSSDRGSYNEQF	ASSSGQMNTEAF 	ASSSMNTEAF 	
ASSRSSTDTQY	ASSSDRGYEQY	ASSSGQNTEAF 	ASSSNNEQF 	
ASSRSSYEQY	ASSSDRNQPQH	ASSSGQNTGELF 	ASSSNNQPQH 	
ASSRSTDTQY	ASSSDRNTEAF	ASSSGQNYEQY 	ASSSNQETQY 	
ASSRSYEQY	ASSSDRNYGYT	ASSSGQNYGYT 	ASSSNSYEQY 	
ASSRSYNEQF	ASSSDRSSYNEQF	ASSSGQPNTEAF 	ASSSNTDTQY 	
ASSRTANTEAF	ASSSDRYEQY	ASSSGQSSYNEQF 	ASSSNTEAF 	
ASSRTASYEQY	ASSSDSNQPQH	ASSSGQTYEQY 	ASSSNTGELF 	
ASSRTATYEQY	ASSSDSQETQY	ASSSGQYNEQF 	ASSSNYEQY 	
ASSRTAYEQY	ASSSDSTDTQY	ASSSTTEAF 	ASSVAGGTDTQY 	
ASSRTDSNQPQH	ASSSDSYEQY	ASSSTTQETQY 	ASSVDSNQPQH 	
ASSRTDSYEQY	ASSSDSYGYT	ASSSTVNTEAF 	ASSVDSNYGYT 	
ASSRTDTQY	ASSSDSYNEQF	ASSSTVSYEQY 	ASSVDSYEQY 	
ASSRTEETQY	ASSSDYEQY	ASSSTYEQY 	ASSVEGNQPQH 	
ASSRTENTEAF	ASSSEETQY	ASSSVDTQY 	ASSVGANTEAF 	
ASSRTGDNQPQH	ASSSGANVLT 	ASSSVYEQY 	ASSVGDTQY 	
ASSRTGELF	ASSSGDEQY 	ASSSWNTEAF 	ASSVGETQY 	
ASSRTGGGNQPQH	ASSSGDSNQPQH 	ASSSYEQY 	ASSVGGGTDTQY 	
ASSRTGGGQPQH	ASSSGDTEAF 	ASSSYGYT 	ASSVGGGYEQY 	
ASSRTGGNQPQH	ASSSGDTQY 	ASSSYMNTEAF 	ASSVGGNQPQH 	
ASSRTGGTDTQY	ASSSGETQY 	ASSSYNEQF 	ASSVGGSSYEQY 	
ASSRTGGTYEQY	ASSSGGAEAF 	ASSSYNSPLH 	ASSVGGSYEQY 	
ASSRTGINQPQH	ASSSGGASTDTQY 	ASSSYQETQY 	ASSVGGTEAF 	
ASSRTGNQPQH	ASSSGGDTQY 	ASSSYSYEQY 	ASSVGLNTEAF 	
ASSRTGNTEAF	ASSSGGEQY 	ASSSYTDTQY 	ASSVGNEQF 	
ASSSNYGYT 	ASSSRSNQPQH 	ASSSYYEQY 	ASSVGNTEAF 	
ASSSPAYEQY 	ASSSRSSYEQY 	ASSTADTQY 	ASSVGQLNTEAF 	
ASSSPETQY 	ASSSRTDTQY 	ASSTDRNTEAF 	ASSVGTDTQY 	
ASSSPGAYEQY 	ASSSRTEAF 	ASSTGETQY 	ASSVGTGGYEQY 	
ASSSPGEQY 	ASSSRTGNQPQH 	ASSTGGNTEAF 	ASSVGYEQY 	
ASSSPGNTEAF 	ASSSRTGSYEQY 	ASSTGGTEAF 	ASSVLAGGTDTQY 	
ASSSPGQGSYEQY 	ASSSRYEQY 	ASSTGLNTEAF 	ASSVNTGELF 	
ASSSPGQGTYEQY 	ASSSSDTQY 	ASSTGMNTEAF 	ASSVQGNQPQH 	
ASSSPGQGYEQY 	ASSSSGADTQY 	ASSTGNEQF 	ASSVQVNTEAF 	
ASSSPGQGYNEQF 	ASSSSGANVLT 	ASSTGNTEAF 	ASSVRGNQPQH 	
ASSSPGQSYEQY 	ASSSSGGNQPQH 	ASSTGPNTEAF 	ASSVRGTEAF 	
ASSSPGTAYEQY 	ASSSSGGQETQY 	ASSTGQGYEQY 	ASSVSNQPQH 	
ASSSPGTEAF 	ASSSSGGTDTQY 	ASSTGSGNTIY 	ASSVSSNQPQH 	
ASSSPQETQY 	ASSSSGGTEAF 	ASSTGSYEQY 	ASSVSTDTQY 	
ASSSPSGANVLT 	ASSSSGGYEQY 	ASSTGTANTEAF 	ASSVSYEQY 	
ASSSPSYEQY 	ASSSSGNQPQH 	ASSTGTDTQY 	ASSVTDTQY 	
ASSSPTDTQY 	ASSSSGNTIY 	ASSTGTSGTDTQY 	ASSVTGGYGYT 	
ASSSPYEQY 	ASSSSGSSYEQY 	ASSTGTSGYNEQF 	ASSVTGLNTEAF 	
ASSSPYNEQF 	ASSSSGYEQY 	ASSTGTVNTEAF 	ASSVTGNTEAF 	
ASSSQETQY 	ASSSSNQPQH 	ASSTGVNQPQH 	ASSVYNEQF 	
ASSSQGADTQY 	ASSSSSTDTQY 	ASSTGVNTEAF 	ASSWDGNQPQH 	
ASSSQGAGTEAF 	ASSSSSYEQY 	ASSTGYGYT 	ASSWDRGMNTEAF 	
ASSSQGETQY 	ASSSSSYNEQF 	ASSTGYTEAF 	ASSWDRGTDTQY 	
ASSSQGGNTEAF 	ASSSSTDTQY 	ASSTMNTEAF 	ASSWDRGYEQY 	
ASSSQGGTEAF 	ASSSSTEAF 	ASSTNEQF 	ASSWDRNTEAF 	
ASSSQGLNTEAF 	ASSSSYEQY 	ASSTQETQY 	ASSWDSNQPQH 	
ASSSQGNEQF 	ASSSSYNEQF 	ASSTQGNTEAF 	ASSWDSSYEQY 	
ASSSQGNQPQH 	ASSSTANTEAF 	ASSTQGYEQY 	ASSWGANTEAF 	
ASSSQGSQPQH 	ASSSTDTQY 	ASSTRETQY 	ASSWGDTQY 	
ASSSQGYEQY 	ASSSTENTEAF 	ASSTSGYNEQF 	ASSWGPNTEAF 	
ASSSQGYGYT 	ASSSTGDEQY 	ASSTSNQPQH 	ASSWGQGNQPQH 	
ASSSQLNTEAF 	ASSSTGDTEAF 	ASSTSTDTQY 	ASSWGQGNTEAF 	
ASSSQMNTEAF 	ASSSTGDYGYT 	ASSTSYEQY 	ASSWGQGSYEQY 	
ASSSQSNQPQH 	ASSSTGELF 	ASSTTGGTEAF 	ASSWGQGYEQY 	
ASSSRAYEQY 	ASSSTGGNQPQH 	ASSTTGNTEAF 	ASSWGQNTEAF 	
ASSSRDRYYEQY 	ASSSTGGSYEQY 	ASSTTGSNQPQH 	ASSWGRNTEAF 	
ASSSRETQY 	ASSSTGGTDTQY 	ASSTTSYEQY 	ASSWGTDTQY 	
ASSSRGGNQPQH 	ASSSTGGYEQY 	ASSTTVNTEAF 	ASSWGTEAF 	
ASSSRGGTDTQY 	ASSSTGIYGYT 	ASSTVNTEAF 	ASSWGYEQY 	
ASSSRGNEQF 	ASSSTGNEQF 	ASSTYSNQPQH 	ASSWTAYEQY 	
ASSSRGQPQH 	ASSSTGNTEAF 	ASSYSGTEAF 	SARGGDTQY 	
ASSSRGTDTQY 	ASSSTGSEQY 	ASSYSGYEQY 	SARGGGETQY 	
ASSSRGTEAF 	ASSSTGSGNTIY 	ASSYSKNTEAF 	SARGGGSYEQY 	
ASSSRGYEQY 	ASSSTGSNQPQH 	ASSYSLLPQEGPSSYNEQF 	SARGGNTEAF 	
ASSSRISNQPQH 	ASSSTGSSYNEQF 	ASSYSLNTEAF 	SARGGNTIY 	
ASSSRLAGGYNEQF 	ASSSTGTDTQY 	ASSYSMNTEAF 	SARGGSYEQY 	
ASSSRLAGSTDTQY 	ASSSTGTNTEAF 	ASSYSNQPQH 	SARGGYEQY 	
ASSSRMNTEAF 	ASSSTGYEQY 	ASSYSNSPLH 	SARGQLNTEAF 	
ASSSRNTEAF 	ASSSTGYGYT 	ASSYSPNEQF 	SARGTGGTEAF 	
ASSSRPMNTEAF 	ASSSTNQPQH 	ASSYSQGYEQY 	SARGTVNTEAF 	
ASSSRQGGTDTQY 	ASSSTNTEAF 	ASSYSRDTQY 	SARITDTQY 	
ASSSRQGGTEAF 	ASSSTNYGYT 	ASSYSREQY 	SARLAGGQETQY 	
ASSSRQGNQPQH 	ASSSTPYEQY 	ASSYSRGSYEQY 	SARQGDTEAF 	
ASSSRQGNTEAF 	ASSSTSGGYEQY 	ASSYSRGTYEQY 	SARQGNTEAF 	
ASSSRQGSTDTQY 	ASSSTSYEQY 	ASSYSRGYEQY 	SARRGNQPQH 	
ASSSRQNTEAF 	ASSSTTDTQY 	ASSYSRLNTEAF 	SARTGDYGYT 	
ASSWTGGNQPQH 	ASSYLGDTQY 	ASSYSSGANVLT 	SARTGGTEAF 	
ASSWTGGNTEAF 	ASSYLGEQY 	ASSYSSLNTEAF 	SARTGNQPQH 	
ASSWTGGSYEQY 	ASSYLGNTEAF 	ASSYSSSYEQY 	SARTGSSYEQY 	
ASSWTGGTEAF 	ASSYLNTEAF 	ASSYSSVYEQY 	SARTGSYEQY 	
ASSWTGNQPQH 	ASSYLQGYEQY 	ASSYSSYEQY 	SARTGYEQY 	
ASSWTGNTEAF 	ASSYLYEQY 	ASSYSTDTQY 	SARTSGGYNEQF 	
ASSWTGQETQY 	ASSYNNEQF 	ASSYSTGNTIY 	SASGDSNQPQH 	
ASSWTGSNQPQH 	ASSYNTEAF 	ASSYSTGYEQY 	SASGGAYEQY 	
ASSWTGSYEQY 	ASSYPDEQF 	ASSYSTYNEQF 	SASRETQY 	
ASSWTSGSTDTQY 	ASSYPGNTEAF 	ASSYSVNTEAF 	SATGNTEAF 	
ASSWTSGSYEQY 	ASSYPGTDTQY 	ASSYSYEQY 	SAWDPNEAF 	
ASSWTVNTEAF 	ASSYQGGTEAF 	ASSYTGNTEAF 	SVDRGNTEAF 	
ASSYDGNTEAF 	ASSYQGSYEQY 	ASSYTGSSYEQY 	SVGEETQY 	
ASSYDSQETQY 	ASSYQNTEAF 	ASSYTSGSTDTQY 	SVGENTEAF 	
ASSYDSSYEQY 	ASSYRDNQPQH 	ASSYVETQY 	SVGGAYEQY 	
ASSYDSTDTQY 	ASSYRDNTEAF 	ASSYYNEQF 	SVGGSNQPQH 	
ASSYEGYEQY 	ASSYRDQPQH 	ASTDTDTQY 	SVGQGNQPQH 	
ASSYGAEAF 	ASSYRDRGSYEQY 	ASTGNTEAF 	SVGQGTYEQY 	
ASSYGANYGYT 	ASSYRDRGYEQY 	ASTGPYEQY 	SVGQNTEAF 	
ASSYGDEQY 	ASSYRENTEAF 	ASTLGETQY 	SVGRNTEAF 	
ASSYGDTQY 	ASSYRGDTQY 	ASTLNTEAF 	SVVRGNTEAF 	
ASSYGGETQY 	ASSYRGETQY 	ASTNTGELF 		
ASSYGGGQPQH 	ASSYRGGETQY 	ASTPDRNTEAF 		
ASSYGGGSTDTQY 	ASSYRGGNQPQH 	ASTPGMNTEAF 		
ASSYGGGTDTQY 	ASSYRGGTEAF 	ASTRGNQPQH 		
ASSYGGGTEAF 	ASSYRGNQPQH 	ASTRGTEAF 		
ASSYGGMNTEAF 	ASSYRGNTEAF 	ASTSSSYEQY 		
ASSYGGNTEAF 	ASSYRGQNTEAF 	ATRTGNTEAF 		
ASSYGGRYNEQF 	ASSYRGSSYEQY 	ATSDFGETQY 		
ASSYGGSGNTIY 	ASSYRGSYEQY 	ATSDLGYEQY 		
ASSYGGSNQPQH 	ASSYRGTDTQY 	ATSDPPTDTQY 		
ASSYGGSTDTQY 	ASSYRNTGELF 	ATSDPSTDTQY 		
ASSYGGSYEQY 	ASSYRSSYEQY 	ATSRDRGSGNTIY 		
ASSYGLSYEQY 	ASSYRSTDTQY 	SAGQGGTEAF 		
ASSYGMNTEAF 	ASSYRYEQY 	SARDRDSSYEQY 		
ASSYGNEQF 	ASSYSASYEQY 	SARDRGYEQY 		
ASSYGNTEAF 	ASSYSENTEAF 	SARDSNQPQH 		
ASSYGQGPYEQY 	ASSYSGANVLT 	SARDSSYEQY 		
ASSYGQGSYEQY 	ASSYSGDTQY 	SAREGGNQPQH 		
ASSYGQMNTEAF 	ASSYSGEQY 	SAREGNQPQH 	 	
ASSYGRNNEQF 	ASSYSGFYEQY 			
ASSYGSNTEAF 	ASSYSGGAYEQY 			
ASSYGSSYNEQF 	ASSYSGGNQPQH 			
ASSYGSTDTQY 	ASSYSGGQETQY 			
ASSYGSYEQY 	ASSYSGGRETQY 			
ASSYGTEAF 	ASSYSGGSGNTIY 			
ASSYGTGNQPQH 	ASSYSGGSYEQY 			
ASSYGTGNTEAF 	ASSYSGGTDTQY 			
ASSYGTGYEQY 	ASSYSGNQPQH 			
ASSYGTNTEAF 	ASSYSGNTEAF 			
ASSYGTSSYEQY 	ASSYSGNTIY 			
ASSYGTSTDTQY 	ASSYSGSQETQY 			
ASSYGTVNTEAF 	ASSYSGSSYEQY 			
ASSYGVNQPQH 	ASSYSGSTDTQY 			
ASSYLAGSYNEQF 	ASSYSGSYEQY 			
ASSYLDTQY 	ASSYSGTASYEQY 			
