# Supplementary material for: Trilineage Sequencing Reveals Complex TCRβ Transcriptomes in Neutrophils and Monocytes Alongside T Cells
Source: Genomics Proteomics Bioinformatics. 2021 Mar 2;19(6):926–36. doi: 10.1016/j.gpb.2019.02.004 (PMC9402791; doi:10.1016/j.gpb.2019.02.004)
Supplement: Supplementary Table S6 — Shared CDR3 sequences between CD14 monocytes and M1 macrophages [file mmc24.rtf]

Table S6 Shared CDR3 sequences between CD14 monocytes and M1 macrophages (individuals I, IV and V)

 	I	IV	V	
 	CD14	M1	CD14	M1	CD14	M1	
unique CDR3	4443	201	6276	14	4107	1438	
shared CDR3	41	41	5	5	303	303	
exclusive CDR3	4402	160	6271	9	3804	1135	
shared CDR3 in %	0.92	20.40	0.08	35.71	7.38	21.07	
+
